# The Polytope Formalism: application to molecular constitution and the prospect of a complete description of Chemical Space

**DOI:** 10.1039/d5sc08813e

**Published:** 2026-01-08

**Authors:** Peter J. Canfield, Maxwell J. Crossley

**Affiliations:** a School of Chemistry, The University of Sydney NSW 2006 Australia maxwell.crossley@sydney.edu.au peter.canfield@Sydney.edu.au

## Abstract

The principles of the Polytope Formalism – first developed for stereoisomerism – are here extended to molecular constitution (including constitutional isomerism), highlighting a deep connection between these two aspects of structure and opening the way toward a unified description of all isomerism. A key feature of this development is that it is based solely on atom connectivity, with explicit inclusion of subvalent and hypervalent species. The resulting complete sets of possible species include traditional isomers and their interconversion intermediates (transition states, higher-order saddle points, *etc.*), providing a powerful tool for elucidating isomerisation mechanisms. This is demonstrated through increasingly complex examples of H-tautomerism. The corresponding networks of species and interconversion pathways map directly onto their associated potential-energy surfaces and thus function as a “discretised encoding” of them. Because this framework accommodates a multidimensional implementation of transition-state theory, it describes and organises the behaviour of the chemical system. Beyond stereoisomerism and molecular constitution, the same mathematical and conceptual principles may be applied to the quantum chemical aspects (nuclear, electronic, and rovibrational states), yielding a fully discretised and physically grounded representation of molecular systems. In doing so, the Polytope Formalism provides a universal framework for the automated exploration of chemical behaviour and Chemical Space – integrating rigorous theory, digital representation, and data-driven discovery within a single coherent framework.

## Abstract 2

The Polytope Formalism establishes a single unifying framework to represent chemical structure, isomerism, and reactivity giving a mathematically rigorous and complete description of Chemical Space.

## Graphical abstract

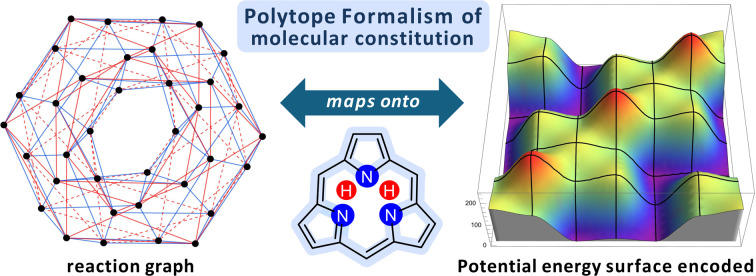

## Introduction

Modern Chemistry evolved with the discovery of a growing list of known elements with seemingly idiosyncratic properties – useful and insightful but lacking any overarching order. Mendeleev's introduction of the periodic table revealed underlying trends and relational patterns among the elements. Only later did the quantum structure of atomic matter expose the deeper foundations of chemical periodicity.

We now face a closely analogous challenge in thinking about chemical isomerism and the quantum descriptors of molecular entities, such as electronic configuration and rovibrational state. Although detailed knowledge of each individual phenomenon is extensive,^1–16^ they are, for the most part, treated within largely separate conceptual frameworks, resulting in an accumulated ad hoc collection of rules, terminology, and nomenclature. No single conceptual framework has been presented that could organise and unify these disparate aspects of molecular identity.

Is such a framework possible? What might it look like, and what practical advantages would it offer? Over the past decade,^17–21^ we have developed a set of formal principles and a mathematical framework – which we name the Polytope Formalism – that addresses these questions and provides a general unifying system capable of describing and analysing any and all chemical possibility.

In earlier work, the polytopal-rearrangement model of stereoisomerism and stereoisomerisation was proposed as a foundation for a systematic, general approach to all forms of stereoisomerism.^18^ We subsequently developed this idea into a rigorous mathematical formalism^19^ and demonstrated its utility through a canonical example: the tetracoordinate centre.^20^ Although that system is relatively simple, its systematic treatment revealed unexpected phenomena, motivating broader investigation and generalisation. That study concluded with a discussion of how stereoisomerism, viewed through polytopal rearrangement could fit within the broader Polytope Formalism.

From this application to stereoisomerism, it is clear that the Polytope Formalism provides a number of important benefits. The first is that, within an application, it systematically generates a mathematically complete and explicitly bounded set of possibilities (configurations), such that questions about what related phenomena remain to be discovered can be answered in a principled way rather than heuristically. The formalism exhaustively describes every possibility. This addresses the “what” produced by the formalism.

The second point is that the formalism organises these configurations into highly structured configuration spaces that recapitulate the underlying physical mechanisms; the formalism describes the “how” by which configurations interrelate and interconvert.

The Polytope Formalism employs Graph Theory to manage the structure of the configuration spaces, which, when combined with energetic information, can encode the associated potential energy surface (PES) and facilitate a direct, mechanistically transparent model for the chemical behaviour under study. Finally, the formalism – through its deliberate focus on minimal abstract descriptors, its mathematical completeness, and the explicit structure of its configuration spaces – all suggest an expanded system of terminology and chemical nomenclature that goes well beyond the scope of current conventions.

In the present work, we apply the Polytope Formalism in detail to molecular constitution (or “constitutional isomerism” in its broadest sense), demonstrating a deep structural and conceptual continuity between this and stereoisomerism – two categories usually regarded as distinct and unrelated.

Historically, the study of molecular constitution has focussed on enumerating isomers and exploring their interrelationships, for example, the *ortho*-, *meta*-, and *para*-positional isomers of substituted benzenes. An early and significant advance in aromatic chemistry came from studies of the products formed during successive chlorination of benzene, whose logical analysis revealed the cyclic structure of the parent compound.^22^

Superexponential scaling is well-known in isomer enumeration,^23–31^ and molecular constitution analysis is no exception. Traditionally, more complex examples have been analysed through combinatorial enumeration methods based on Burnside's Lemma and the Redfield–Pólya theorem.^5–14^ Although powerful, these techniques do not, in themselves, readily convey explicit relational or mechanistic information; resulting lists of isomers are essentially unstructured and emphasise counting rather than isomer interrelationships.

Application of the Polytope Formalism to molecular constitution generates traditional isomers together with interconversion intermediates, thereby mapping the mechanistic landscape in a structured, relational way. When directed toward the subset of “atoms that matter” – an implementation we term “
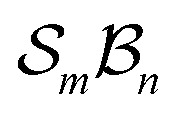
-partitioning” – the method becomes a tractable and chemically interpretable tool for analysing chemical systems and reactions. We illustrate this approach through several progressively complex examples of H-tautomerism.

The strengths of the Polytope Formalism and its broader implications are examined, including its high-level conceptual structure and its potential application to all forms of isomerism.

Finally, we outline future challenges and opportunities, describing how the Polytope Formalism can be extended to encompass the other descriptors of chemical entities and their molecular quantum-state characteristics. In doing so, the formalism establishes a single, internally consistent conceptual basis for describing all chemical properties and processes. The Polytope Formalism thus offers a principled framework for rigorously defining, comprehensively mapping, and systematically exploring Chemical Space itself.

## Foundational requirements and proposals for application of the Polytope Formalism to molecular constitution

### Atom connectivity in place of canonical form

The prevailing approach to molecular constitution – and to constitutional isomerism in particular – is founded on the use of canonical forms.^32,33^ In these representations, localised bond multiplicities (integer bond orders) are assigned to each atom–atom connection, and valence requirements are satisfied to yield what is commonly called a valence structure. This paradigm has proved both powerful and foundational, underpinning modern digital encodings of molecular structure. For discussion of bond order, see the SI, Section SI6.

The constraints intrinsic to canonical forms, however, restrict their generality, especially for processes involving bond formation and cleavage. In our implementation of the Polytope Formalism for molecular constitution, we therefore focus exclusively on atom connectivity, independent of bond order – an approach consistent with the IUPAC definition of connectivity.^32,33^

**Fig. 1:**
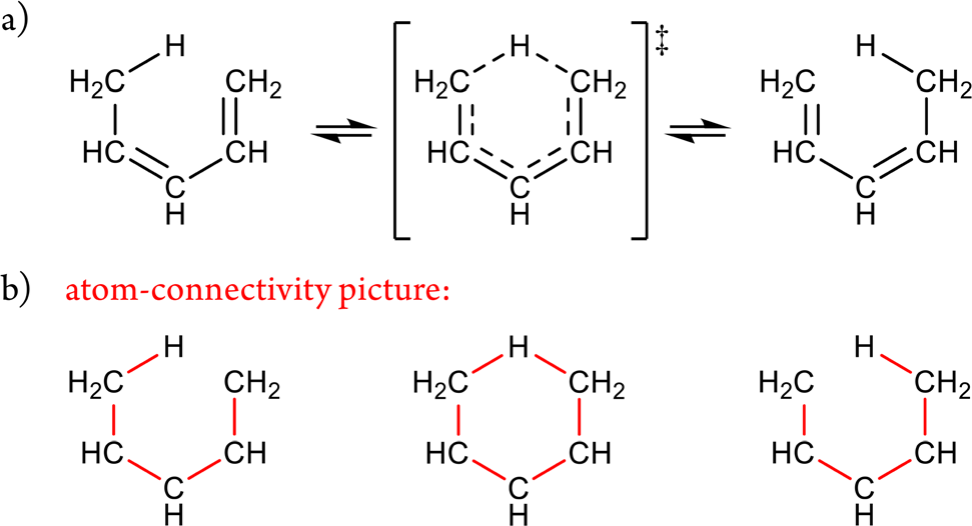
(a) [1,5]-Sigmatropic shift depicted using standard representation. (b) The atom-connectivity picture corresponding to (a) including explicit H-atoms.

The limitations of canonical representations are illustrated by a [1,5]-sigmatropic rearrangement (Fig. 1). In the conventional canonical form (Fig. 1a), both the reactant and product can be depicted using formal single and double bonds, while the transition-state (TS) structure is drawn to reflect the evolving bonding pattern. Crucially, this TS cannot itself cannot be represented canonically and therefore lies outside the standard framework.

In contrast, Fig. 1b presents these same three structures using explicit atom-connectivity representations. Within this scheme, the reactant, transition state, and product each possess distinct and well-defined atom–atom connectivity patterns and are thus treated as separate “isomers” – in the broadest sense – on equal conceptual footing. While this atom-connectivity formulation enables a broader and more inclusive description of chemical transformations, certain reaction classes – such as cycloadditions and chelotropic reactions – may, in their simplest treatment, be under-sampled within the configuration space. This apparent limitation is examined further in the Discussion section.

We define an interatomic connection strictly as a relationship between pairs of atoms. This excludes multi-centre bonding representations, such as three-centre bonds^34^ or the side-on bonding conventions used in polyhapto coordination systems.^32,33^ Such bonding types, however, can be reformulated into pairwise atom-connectivity patterns, as detailed in the SI, Section SI7.

Within the conceptual scope of molecular constitution, our concern is not with the physical nature of the bond (*e.g.*, covalent *versus* ionic) but simply with the existence of a connection between two atoms that satisfies the formal definition of a chemical bond.^32,33^ Although the mathematics of the formalism is developed entirely in terms of atom connectivity, for clarity and familiarity we depict structures in the conventional canonical (valence-bond) style throughout this work.

### Desmotropic processes

Any process that changes atom connectivity can, in the broadest sense, be described as desmotropic.^35–37^ Since molecular constitution is defined by differences in atom connectivity within a fixed atomic set, a desmotropic process refers to transformation between such chemical entities, which we loosely describe as isomers.

This general term encompasses more specific categories such as pericyclic and sigmatropic rearrangements, providing a unified vocabulary for all connectivity-altering reactions.

For concerted processes, we introduce the symbol R^c^_de_ to the literature denoting rearrangement, concerted, desmotropic. The molecularity can be specified as R^c^_de_1, R^c^_de_2, …R^c^_de_*k*, for unimolecular, bimolecular, and higher order cases, respectively.^37^

Mechanistically, every R^c^_de_ process involves a bond-stretching vibrational mode, – ideally, a local mode – distinguishing it from unimolecular stereoisomerisation processes, which are dominated by angle-changing vibrational modes.^18–20^

## The Polytope Formalism applied to molecular constitution

The use of atom connectivity enables an exceptionally simple yet comprehensive mathematical treatment of molecular constitution in its most general form. For a given set of atoms numbered 1 to *N*, all mathematically possible molecular graphs can be generated and organised according to their interrelationships, without imposing constraints such as valence, minimal connectivity, or even the requirement that the entities be realisable in three-dimensional space. Since there can be at most one atom connection (a bond irrespective of bond order, or “graph edge”) between any two atoms (the “graph vertices”), all resulting molecular graphs are simple graphs.^38^

**Fig. 2:**
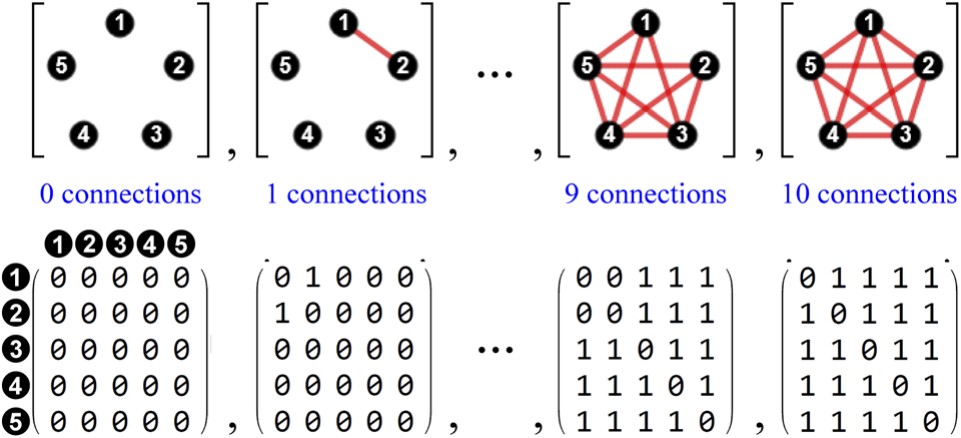
Ordered listing of unrestricted atom-connectivity configurations for the 5-atom class. Below the molecular graph of each atom-connectivity configuration is shown the total number of atom connections along with its adjacency matrix (representing an abstract polytope).

The enumeration of all such unrestricted atom-connectivity configurations for N atoms corresponds to the sum of all possible arrangements of *k* atom connections, where *k* ranges from 0 to the maximum number of connections.^25^ Hence, for N atoms, the number of mathematically possible atom-connectivity configurations equals 2^*N*(*N*−1)/2^. For example, in the 5-atom system illustrated in Fig. 2, the complete set terminates in a single configuration comprising a total of ten atom–atom connections (the maximum possible) yielding 1024 distinct possibilities overall.

This enumeration rises superexponentially and is formally of the order 
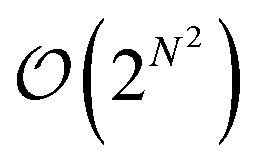
 and thus exceeds the exponential scaling typical of standard isomer-enumeration approaches,^25^ making it one of the more computationally demanding problems in chemical combinatorics.

Why then pursue an approach seemingly more complex than existing schemes? Previous methods for isomer enumeration have depended on chemical constraints such as atomic valence and steric limitations to simplify the problem.^25,27–31,39–42^ In addition, they typically required that all atoms form a single connected molecular entity – a useful assumption when the task was limited to counting isomers, but one that necessarily excluded molecular graphs corresponding to reaction intermediates or bimolecular scenarios.

Our approach adopts a broader perspective, considering all chemical entities derivable from a given atomic set and the relationships among them. This framework naturally accommodates both association–dissociation processes and unimolecular transformations.

A further advantage lies in its mathematical completeness. By generating and organising molecular graphs solely on the basis of atom connectivity, the resulting configuration spaces are exhaustive: no possibilities are omitted or put another way, there are no unknowns within this analysis. The total size of this space, 2^*N*(*N*−1)/2^, defines an absolute upper bound for any enumeration. When certain connectivity patterns are excluded – for instance, for chemical or physical reasons – the resulting system represents a rigorously defined subset of the complete space. The relationship between complete and constrained sets is thus explicit: exclusions are clearly defined, and any unexplored portion of the full space remains well characterised even if not analysed in detail. In this sense, there are no unknown unknowns.

### Importance of subvalent and hypervalent species

A key strength of the Polytope Formalism is its explicit inclusion of atom-connectivity configurations containing subvalent or hypervalent atoms. Such species arise naturally as intermediates in desmotropic reactions, allowing the formalism to capture processes that involve both bond formation and bond cleavage.

In practice, the permissible degree of subvalency or hypervalency for any atom depends on the chemical elements involved and their steric limitations. These constitute explicit constraints within the analysis. For example, in the five-atom system of Fig. 2, the final configuration – with every atom connected to every other – is physically unrealisable in 3D space 
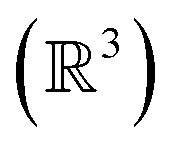
 and can therefore be excluded on that basis.^43^ This condition represents the fundamental steric constraint (see SI Section SI10) significantly reducing the superexponential scaling.

### Abstract polytopes

In stereoisomerism, the relevant polytopes correspond to geometric objects that can also be represented abstractly in matrix form. In the context of molecular constitution,^44^ the different atom-connectivity configurations represent distinct patterns or permutations of connectivity. This combinatorial structure can likewise be expressed in matrix form and, by analogy with the stereochemical case, these may be regarded as abstract polytopes.^21^ These abstract polytope species – henceforth referred to simply as species – and their matrix representations are equivalent to the adjacency matrices of simple molecular graphs used in cheminformatics.^45^ These diagonally symmetric matrices encode which atoms are connected in a molecule, irrespective of bond order. In Fig. 2, each atom-connectivity configuration is shown alongside its corresponding adjacency matrix.

### Organisational principles and taxonomic hierarchy

To manage the complexity of results produced by the Polytope Formalism, we employ the same hierarchical taxonomic system – class, family, genus, and species – introduced previously for stereoisomerism.^18–21^ Its usefulness and flexibility are demonstrated below and throughout this work.

A class refers to the system of atoms to which the connectivity permutations are applied. Family is defined by the total number of atom connections (the “edge count”), genus by the general pattern of connectivity (molecular-graph isomorphism), and species by a specific arrangement of atom connections for that atomic set (see the SI, Sections SI3 and SI5, for further details).^23^

**Fig. 3:**
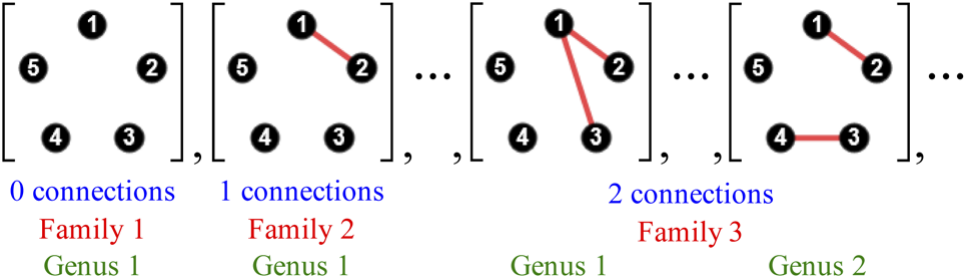
Several species from the 5-atom class demonstrating taxonomic distinctions.

For the five-atom class (see Fig. 3), the atom-connectivity species can be grouped into families defined by total edge count – from 1 through 10. Within each family, species are further classified into genera according to their connectivity pattern. For example, in the five-atom class, Family 1 comprises one genus with a single species having no connections; Family 2 contains one genus with ten species, each having a single atom–atom connection; Family 3 includes two genera – 30 species where two connections share a common atom (forming an atom–atom–atom unit) and 15 species where the two connections are independent (two separate atom–atom units). The complete classifications for the 3-, 4-, and 5-atom classes are presented in the SI, Section SI9.

Later sections introduce a more focused implementation of the formalism, where the definitions of family and genus are modified slightly; the worked examples illustrate these refinements. Once specific elements are assigned to atoms, this taxonomy becomes chemically meaningful and directly applicable.

### Reaction graphs – traditional reaction representation – potential energy surface – Chemical Space relationships

The interrelationships among the connectivity-permuted species generated by the formalism can be represented as reaction graphs (distinct from molecular graphs, which depict atom–atom connections). In reaction graphs, species are shown as nodes (graph vertices) and their pairwise relationships as edges, each corresponding to an R^c^_de_ process – that is, an elementary desmotropic reaction. The complete reaction graph therefore encodes the entire network of desmotropic processes and, by extension, the network of all possible reactions.

Adjacency between two species in a reaction graph – and the precise nature of that adjacency – is defined rigorously in the SI, Section SI15. Here we focus on the simplest case, where adjacent species differ by only a single atom–atom connection.

By assigning representative geometries and specifying all relevant quantum-mechanical details (electronic, rovibrational, and nuclear details), an energy value can be associated with each species. Transformation between adjacent species then traces a path on the associated PES, with each reaction-graph edge defining a segment of the reaction coordinate. Collectively, the reaction graph maps onto the PES topology of the system: the species generated by the formalism sample the key points of the surface, capturing the topology of the underlying chemical landscape. This correspondence is intrinsic to the Polytope Formalism and applies equally to stereoisomerism and molecular constitution.

**Fig. 4:**
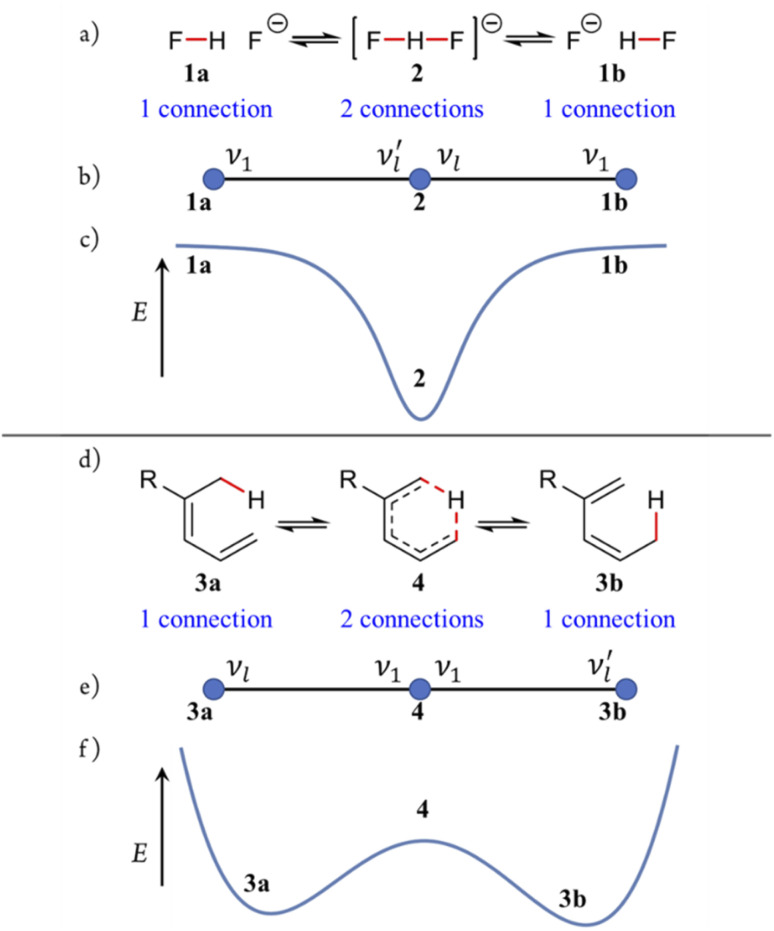
Two desmotropic chemical reactions, reaction-graph representations, and potential energy surface relationships for (a–c) dissociation (R^c^_de_2 process) of bifluoride anion 2 into [HF + fluoride] 1, and (d–f) the [1,5] sigmatropic rearrangement (R^c^_de_1 process) of 3a to 3b*via*4. Coloured atom connections are permuting. *ν*_*l*_ denotes local bond-stretching modes. *ν*_1_ denotes the imaginary mode.

Two illustrative examples are shown in Fig. 4. Fig. 4a–c depict the unsymmetrical dissociation of the bifluoride anion 2 into 1a and 1b; Fig. 4d–f show the [1,5]-sigmatropic rearrangement of 3a to 3b*via*4. Despite their mechanistic differences, both reactions yield reaction graphs of identical topology – three vertices connected sequentially by two edges. Although their PESs (Fig. 4c and f) differ in detail, their graphs are topologically isomorphic. This shared topology among distinct chemical systems exemplifies a central feature of the Polytope Formalism: while the details of the energetics will vary, the underlying connectivity of the configuration space remains invariant.

Within the formalism, certain species correspond to well-defined PES critical points, whereas others must be defined qualitatively. Each graph edge corresponds to an elementary reaction coordinate associated with local bond-stretching vibrational modes; sequences of edges may combine to form a complete reaction coordinate in the traditional sense (further details are provided in the SI.)

Given a reaction graph for a chemical system, the principles of transition-state theory (TST)^46^ can be applied by associating the relative energies, gradients, and curvatures of the constituent species with their respective graph vertices. In this way, the reaction graph encodes the essential features of the PES and serves as a compact representation of the chemical landscape of the system.

Importantly, not all atoms in a system need participate in connectivity permutations (as in Fig. 4d), and the full range of possible permutations need not be applied. Such restrictions constitute imposed constraints. For example, in both reactions shown in Fig. 4, the active hydrogen atom is constrained to maintain at least one bond throughout the transformation.

### Modular structure theorem

A powerful feature of the Polytope Formalism – independent of its specific implementation – is the highly structured nature of its configurations and configuration spaces, which lend themselves naturally to graph-theoretic treatment. From this emerges the *Modular Structure Theorem*, which can be stated as follows:

**Modular structure theorem**

For any chemical system, the configuration space resulting from imposed constraints (for example, reduced atom count or limited connectivity) constitutes a subspace of the corresponding unconstrained configuration space.

Although seemingly self-evident, graph theory permits this statement to be formalised with full mathematical rigour (see SI, Section SI12). The theorem guarantees that focused analyses are properly embedded within larger, unconstrained spaces, enabling tractable exploration of reduced systems while maintaining a defined relationship to their generalised counterparts.

Smaller, constraint-based analyses are often computationally more manageable, whereas larger configurational spaces can be generated by progressively relaxing these constraints – for instance, by adding atoms, incorporating stereoisomerism, or considering alternative quantum states. The following section introduces a general method for conducting such focused analyses.

## A practical approach: 
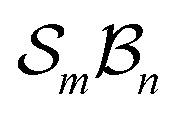
 partitioning

Given the superexponential growth inherent to combinatorics-based analyses, the Modular Structure Theorem enables focussed studies in which atom-connectivity permutations are restricted to a subset of atoms embedded in a larger framework (as in Fig. 4d). When constrained by valence,^47^ and applied to a single molecular entity, the Polytope Formalism (PF) of molecular constitution reproduces the results of established constitutional-isomer enumeration methods.^25^

This partitioning strategy is broadly applicable because most reactions involve only a few bond changes within an otherwise fixed scaffold. Rearrangement reactions and “bond-walk” processes^17,21^ – from simple tautomerism to complex phenomena such as ion transport through transmembrane channels^48^ – fit naturally within this framework.

**Fig. 5:**
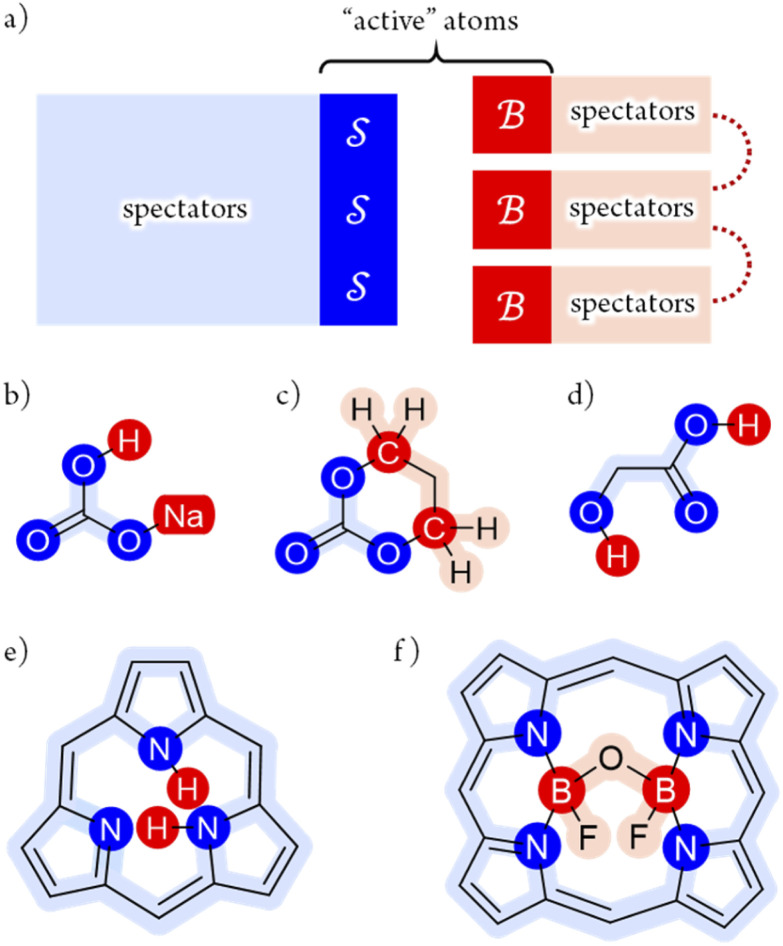
(a) partitioning of 
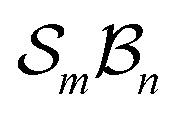
 molecular systems into bonding sites 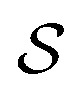 and bonders 
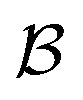
 that undergo atom-connection permutations, and spectator atoms and bonds. (b) NaHCO_3_ partitioned as 
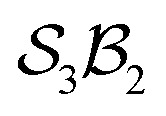
 with three identical O-atom sites and distinct bonders: H-atom and Na-atom. (c) A cyclic carbonate ester partitioned as 
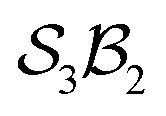
 with three identical O-atom sites and two linked identical methylene C-atom bonders. (d) Glycolic acid partitioned as 
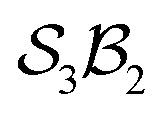
 with two identical and one distinct O-atom sites, and two identical H-atom bonders. (e) Free-base subporphyrin partitioned as 
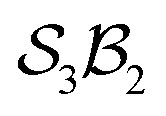
 for H-tautomerism. (f) B_2_OF_2_-porphyrin partitioned as 
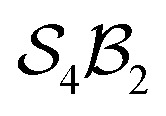
 to describe strepsisomerisation.^17,21^

Fig. 5 illustrates the approach. The subset of active atoms permitted to undergo connectivity permutation is divided into two roles: bonders 
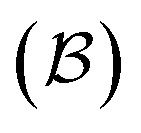
 – the mobile atoms – and bonding sites 
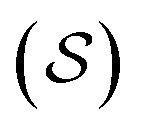
, typically anchored within one or more larger molecular fragments. Distinct atom-connection configurations arise solely from permuting connectivity between bonders and sites. All remaining atoms are spectators, whose connectivities remain fixed throughout the transformation. With *m* bonding sites 
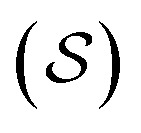
 and *n* bonders 
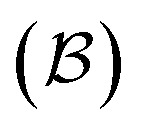
, the system is said to belong to the 
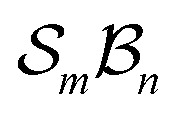
 class.

The examples in Fig. 5b–d each realise 
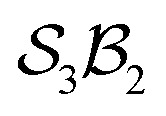
 partitioning, with three O-atom sites (darker blue). In Fig. 5b (NaHCO_3_), the bonders are H and Na (darker red), and the central C-atom is a spectator within the site complex; the three sites are equivalent (
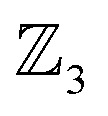
 site symmetry – see SI4 for why space groups are not used to define connectivity symmetry). In Fig. 5c, the site complex is again “carbonate”, but the bonders are two equivalent methylene C-atoms linked *via* spectators. Fig. 5d treats glycolic acid, still 
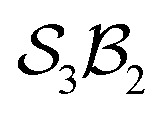
, but with broken site symmetry: two equivalent O-atoms (carboxylate) and one distinct O-atom (alcohol).

Within this 
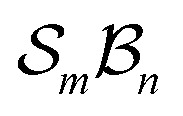
 framework, the same set of connectivity permutations applies to all systems in Fig. 5b–d. Consequently, each yields the same numbers of families and species – 6 families and 49 species.

Fig. 5e and f illustrate cases in which the bonders lie inside the site complex. In Fig. 5e, free-base subporphyrin (free-base triphyrin[1.1.1]) is prepared for H-tautomerism: the H-atom bonders may connect to any of three N-atom sites, and the site complex (subporphyrin dianion) exhibits 
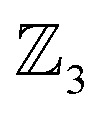
 site-connectivity symmetry. In Fig. 5f, a B_2_OF_2_–porphyrin complex is partitioned as 
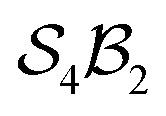
 to investigate the strepsisomerisation^17,21^ bond-walk mechanism: four N-atom sites and the macrocycle (porphyrindiyl) exhibit 
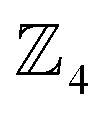
 site symmetry, the two B-atoms act as bonders, and O and F are spectators within the bonder complex.

### Worked example: H-tautomerism in free-base subporphyrin monoanion

**Fig. 6:**
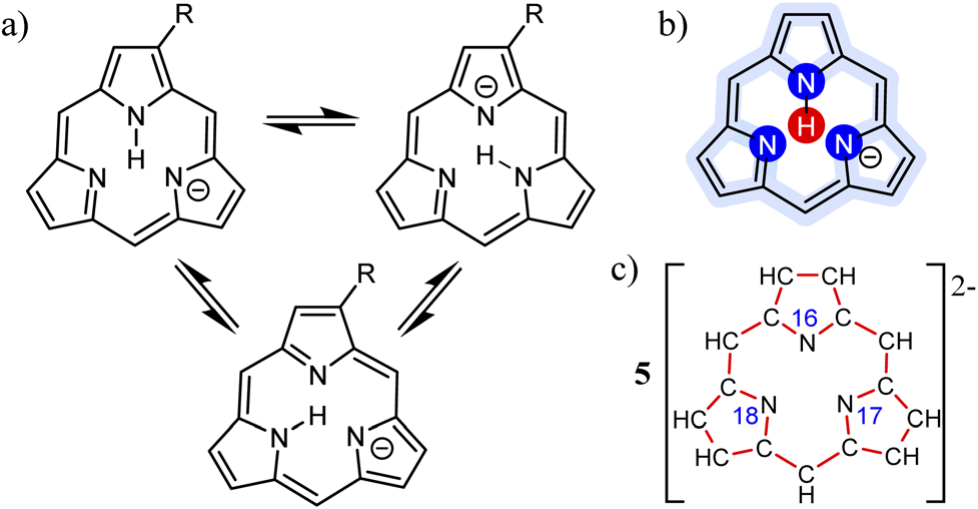
Analysis of tautomerism in free-base subporphyrin monoanion. (a) Traditional view on a symmetry-broken macrocycle. (b) 
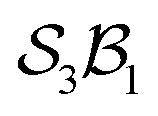
 partitioning with three site N-atoms (dark blue) and one bonder H-atom (red shading). All other atoms are spectators (light blue shading). (c) Atom-connectivity picture of the “subporphyrindiyl” fragment 5 exhibiting 
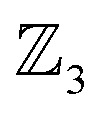

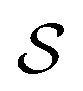
-symmetry. Explicit H-atoms and 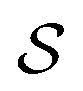-atom locants (N-atoms) are shown.

We illustrate the Polytope Formalism with H-tautomerism in the free-base subporphyrin monoanion (Fig. 6). The core questions are: (i) which species are possible, and (ii) how do they interconvert?

**Fig. 7:**
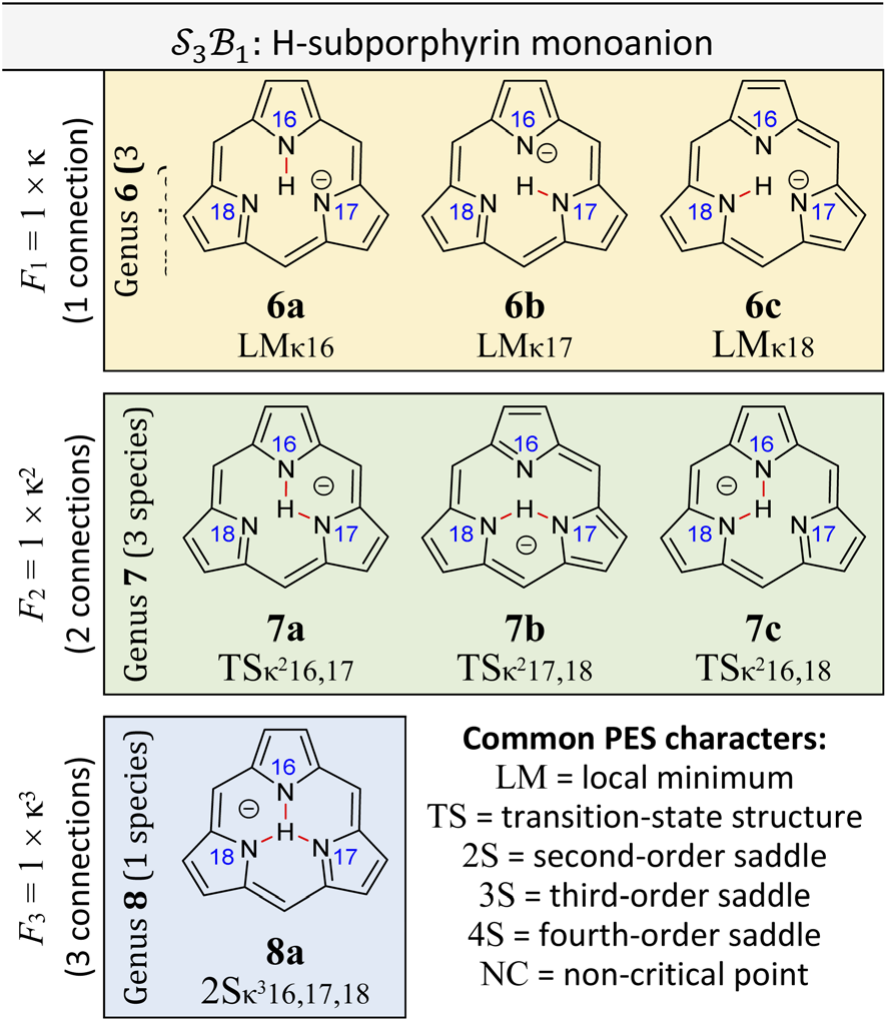
All species of the 
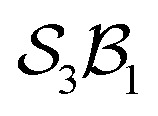
 class exhibiting 
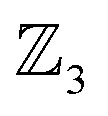

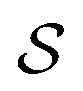
-symmetry under the condition of nondissociation, as exemplified for tautomerism in free-base subporphyrin monoanion. All species are collected into genera and families and assigned a descriptive and compact label. A short listing of common PES characters are shown.

**Fig. 8:**
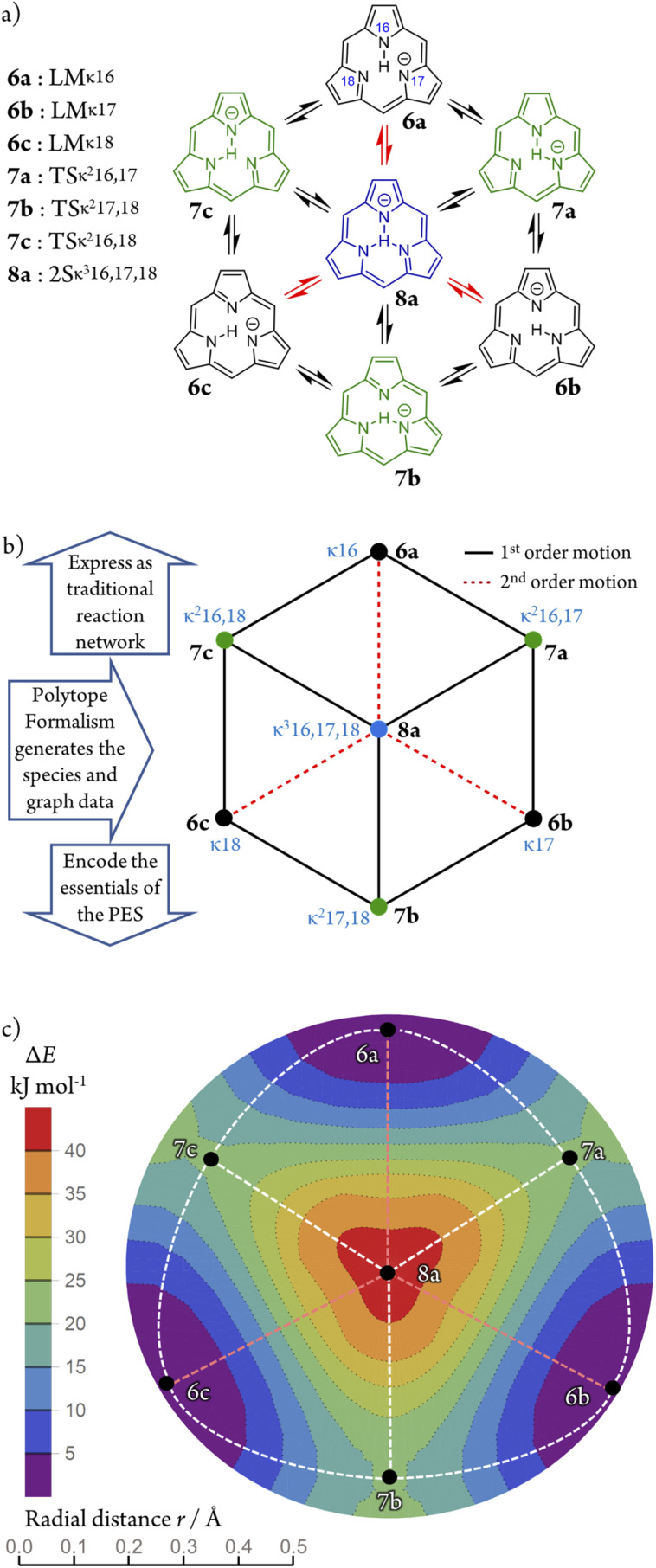
Tautomerism in free-base subporphyrin monoanion as 
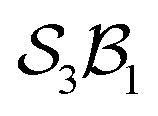
 partitioning, graph representation, and corresponding PES relationships. (a) Traditional reaction network of three isomers (LM), transition-state structures (TS), and second order saddle-point transition-state structure (2S). (b) Undirected graph representation corresponding to (a). Atom-connectivity lists annotated in blue. (c) DFT-calculated PES scan for the chemical system showing locations of species. Dashed lines show minimum-energy pathways (MEPs). Graph topology in (b) corresponds to the topology of the MEPs on the PES. The formalism generates the graph (b) from which the reaction network (a) ensues, and many essential features of the PES (c) can be encoded.

**Fig. 9:**
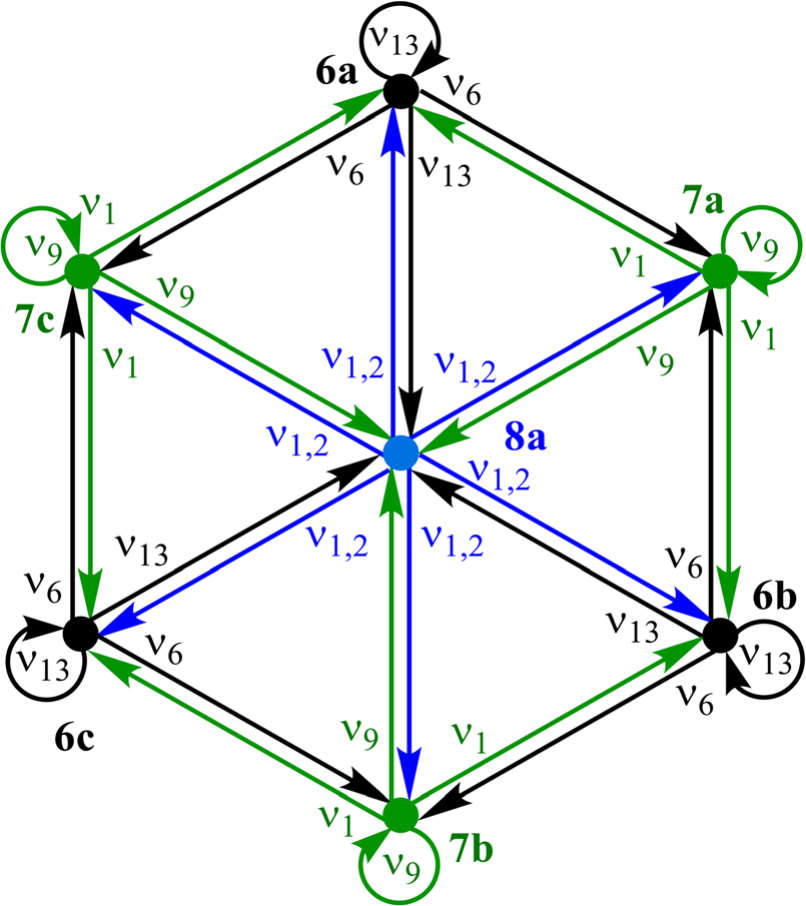
Vibration-annotated directed desmotropic reaction graph for tautomerism in free-base subporphyrin monoanion. Each graph edge is annotated by the DFT-calculated normal vibrational mode of the source species that affects the transformation. For 7a*ν*_1_ denotes the single imaginary bond-stretching vibration, and for 8a*ν*_1,2_ denotes the doubly degenerate imaginary bond-stretching vibrations.

**Fig. 10:**
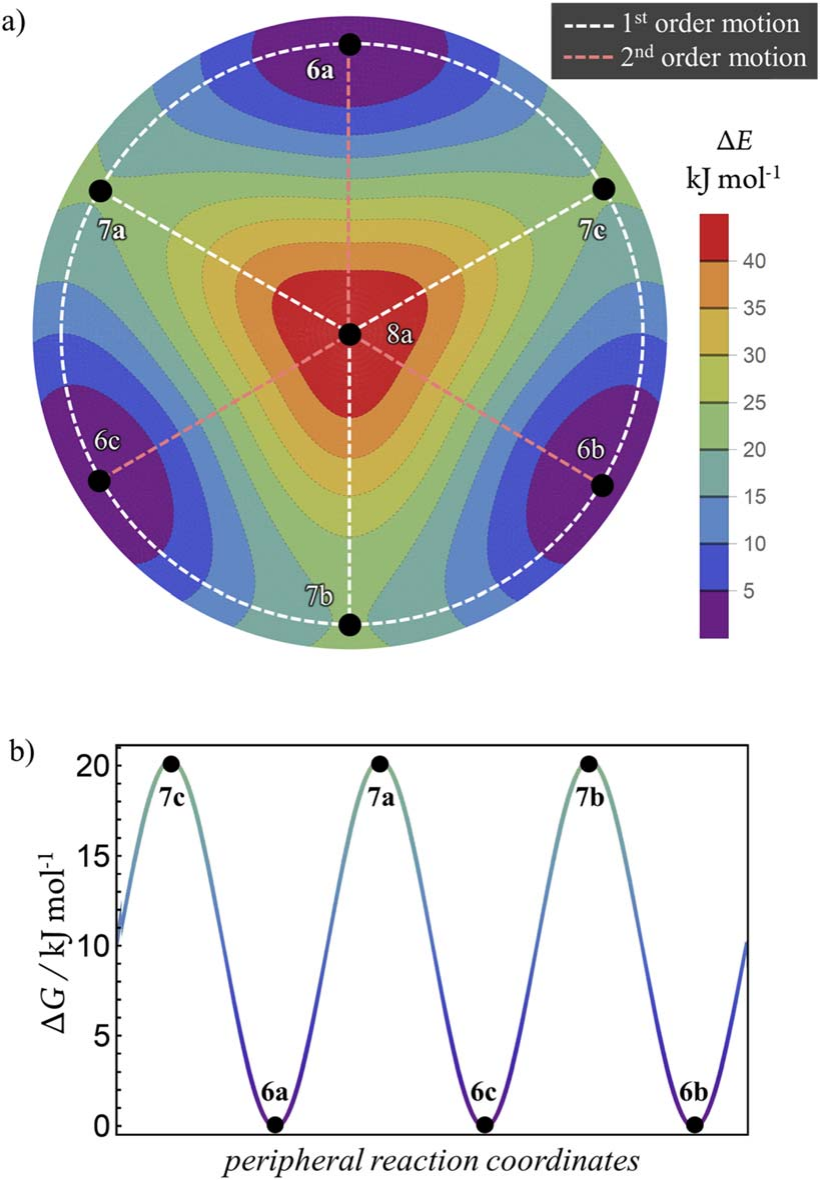
Minimal interpolated approximation of the PES for tautomerism in free-base subporphyrin monoanion using the 
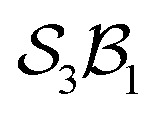
 partitioning approach. (a) The 2D PES overlain by the reaction graph (labelled points and dashed lines). (b) The 1D periodic PES interpolated from genera 6 and 7 – the outer periphery in (a) with the same energy scale.

Focussing first on the high-symmetry system R = H (Fig. 6a), we partition the monoanion as 
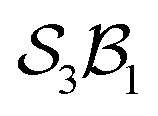
 (Fig. 6b). The “subporphyrindiyl” fragment 5 (Fig. 6c) – the monoanion without the inner hydrogen – exhibits 
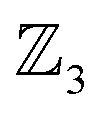
 symmetry from the atom-connectivity perspective. Under the nondissociation condition (single molecular entity), full connectivity permutation between the three N-atom sites and the single H-atom bonder yields seven species (Fig. 7 and in Fig. 8–10):

• Family F_1_ (κ) – genus 6: three degenerate species 6a–c, each with one bonder–site connection.

• Family F_2_ (κ^2^) – genus 7: three species 7a–c, each with the bonder bridging two sites.

• Family F_3_ (κ^3^) – genus 8: the single species 8a, with the bonder triply bridging all three sites.

This “one-genus-per-family” pattern is not general; later examples display richer taxonomic structures. When 
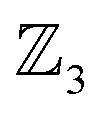
 site symmetry is broken (R ≠ H, Fig. 6a), the three κ species (6a–c) become chemically distinct.

### Naming the species and defining species–species relationships

Because the formalism generates many distinct species, we require concise, unambiguous labels. The abstract-polytope (adjacency-matrix) representation is not human-readable, so we adopt a compact connectivity-based κ notation (adapted from the κ convention^49^) combined with PES character (LM, TS, 2S, …). Thus, in Fig. 7:

• LMκ16 denotes species 6a, a local minimum-structure with the bonder attached to site atom 16;

• TSκ^2^16,17 denotes species 7a, a transition-state structure with the bonder bridging site atoms 16 and 17;

• 2Sκ^3^16,17,18 denotes species 8a, a second-order saddle point structure.

Links to systematic nomenclature and formal definitions are given in the SI, Section SI11.

The notation also encodes relational information. Species LMκ16 and TSκ^2^16,17 share site atom 16 and differ by one connectivity change (add/remove connection to site 17). We term this a first-order motion and the connecting process an R^c^_de_ first-order step. Two connectivity changes (*e.g.*, one bonder changes by two links, or two bonders change by one link each) define a second-order motion. These order concepts are useful when discussing pathways on their associated PES; formal definitions are given in the SI, Section SI15.

Typically, a first-order relationship corresponds to a ±1 change in the number of negative Hessian eigenvalues dominated by bond-stretching character (*e.g.*, LM ⇌ TS, 6a ⇌ 7a; TS ⇌ 2S, 7a ⇌ 8a; Fig. 8). A second-order relationship usually corresponds to ±2 such eigenvalues (*e.g.*, LM ⇌ 2S, 6a ⇌ 8a; Fig. 8). Notably, certain pericyclic reactions (*e.g.*, Diels–Alder) deviate from this simple correspondence; see Discussion and SI Section SI18.

### Multidimensional reaction landscape for H-tautomerism in free-base subporphyrin monoanion

The atom-connectivity configuration differences for a pair of species indicates whether they are “adjacent” on their reaction graph, that is, whether a graph edge exists between those species. For unimolecular (non-dissociative) H-tautomerism in free-base subporphyrin, only first-order and second-order relationships occur. Fig. 8b shows the combined first- and second-order motions graphs, corresponding to the traditional reaction network in Fig. 8a (with matching layout).

The corresponding Density-Functional Theory (DFT) calculated qualitative PES for the migrating H atom, constructed in a real-space polar coordinate system, is shown in Fig. 8c. The seven species and their minimum-energy pathways (MEPs) are indicated. The motions graph (Fig. 8c) maps isomorphically onto the real-space PES and its MEPs: white dashed paths correspond to first-order motions, light-red dashed paths to second-order motions.

Inspection of the optimised species reveals that bond-stretching vibrational modes (*e.g.*, one imaginary frequency for 7a, two for 8a) distort geometries along the MEPs toward reaction-graph neighbours, consistent with the motion order.

A directed version of this graph (Fig. 9), following the stereoisomerism treatment,^18–20^ annotates each edge with the DFT normal mode of the source species that drives the transformation. Self-loops are required to account for both displacement directions of a vibration and, in this example, represent the steep PES walls at the periphery.

The standard approach within quantum chemical modelling's approach to Transition-State Theory (TST), is to determine what species are important in a chemical reaction and assign energies to them. Given the Polytope Formalism generates a list of species and their reaction graph, it is a simple exercise to apply TST here noting that the reaction graph edges each represent piecewise reaction coordinates. Not only does this yield a multidimensional implementation of TST, it also represents a compact discretised encoding of the PES (Fig. 10). The two PES plots in Fig. 10a, generated by simple interpolation from this information, are isomorphic to the real-space PES in Fig. 8c. Although reaction graphs are discrete and abstract – not geometric coordinates – their isomorphism with real-space PESs demonstrates a direct link to the underlying geometry. This capability to structure multidimensional TST becomes increasingly valuable as chemical complexity grows, as shown in subsequent sections.

## Tools for more complex systems

### Enumeration, cataloguing, and graphing of the species of the 
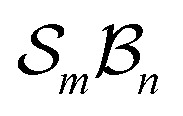
 class

To facilitate structured listing of atom-connectivity species, software for generating families, genera, and species within the 
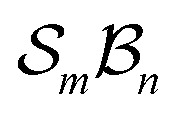
 class (under the assumptions of nondissociation and 
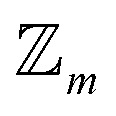
 site symmetry) is provided in the SI.

A further chemically intuitive restriction is to limit the maximum number of bonders at a single site atom. In such cases, the modified class is denoted 
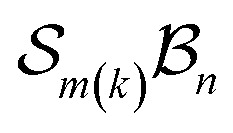
, where *k* specifies the maximum number of bonders permitted at any site atom.

Starting from the intrinsic form of each atom-connectivity species (see Fig. 3 and 4), a simple atomic model of sites and bonders can be generated. This transition from abstract atom connectivity to plausible 3-dimensional molecular geometry naturally introduces steric and stereoisomeric considerations. Consequently, more than one geometry may correspond to a single atom-connectivity species, as isomers are now defined by both connectivity and local/global stereochemistry. Since each dimension of variation is finite, for example a pair of enantiomers, the number of possible structures remains countable.

For a given 
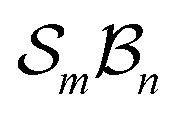
-partitioned system under nondissociation, the total numbers of families (*N*_f_), genera (*N*_g_), and species (*N*_s_) are summarised in Tables 1–3. Expanded tables and mathematical expressions for these quantities, up to 
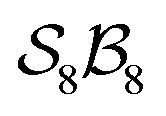
, are provided in SI, Section SI13.

**Table 1:** Numbers of families *N*_f_, for 
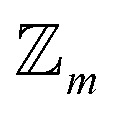
 site symmetric 
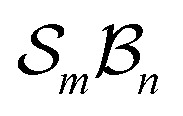
 and 
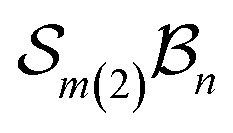
 classes under the constraint of nondissociation

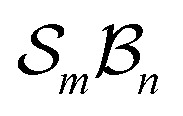 families *N*_f_					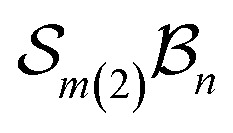 families *N*_f_				
*m\n*	1	2	3	4	*m\n*	1	2	3	4
1	1	1	1	1	1	1	1	—	—
2	2	3	4	5	2	2	3	—	—
3	3	6	10	15	3	3	6	6	—
4	4	10	20	35	4	4	10	13	11

**Table 2:** Numbers of genera *N*_g_, for 
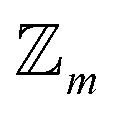
 site symmetric 
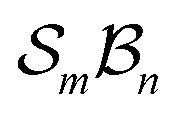
 and 
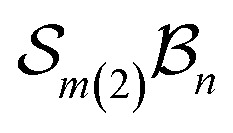
 classes under the constraint of nondissociation

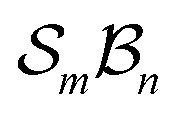 genera *N*_g_					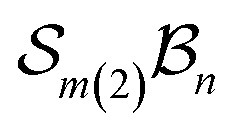 genera *N*_g_				
*m*\*n*	1	2	3	4	*m\n*	1	2	3	4
1	1	1	1	1	1	1	1	—	—
2	2	4	6	9	2	2	4	—	—
3	3	9	23	51	3	3	9	10	—
4	5	28	124	494	4	5	28	57	64

**Table 3:** Numbers of species *N*_s_, for 
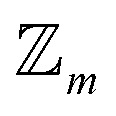
 site symmetric 
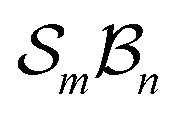
 and 
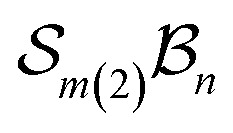
 classes under the constraint of nondissociation

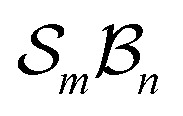 species *N*_s_					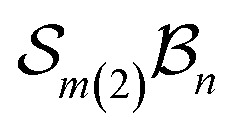 species *N*_s_				
*m\n*	1	2	3	4	*m\n*	1	2	3	4
1	1	1	1	1	1	1	1	—	—
2	3	9	27	81	2	3	9	—	—
3	7	49	343	2401	3	7	49	174	—
4	15	225	3375	50 625	4	15	225	1680	6510

Associated graphing utilities are also included for visualisation and basic graph-theoretic analysis (Section SI14).

### From abstract connectivity to physical structures

Not all configurations envisaged by this enumeration will be chemically realisable owing to steric limitations in connecting the bonding sites (and possibly the bonders, see Fig. 5). However, whenever a physically plausible molecular framework can be constructed for a given species, computational modelling may be applied to generate a three-dimensional structure.

As a practical first step, molecular mechanics offers a suitable starting point due to its explicit atom-connectivity definitions. Manual modelling suffices for smaller systems, but the true strength of the method lies in its automation, enabling systematic generation, evaluation, and comparison of connectivity-defined chemical structures.

## Implementation of the formalism for more complex chemical systems: 
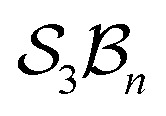
 classes

To illustrate more nuanced features of the Polytope Formalism, we examine two representative systems: neutral free-base subporphyrin, analysed as the 
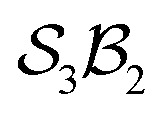
 class, and triphyrin[2.2.2], analysed as the 
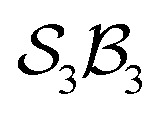
 class, where the number of bonders that can connect to any one site is limited to two – written 
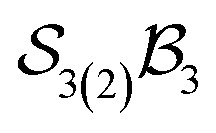
.

### An 
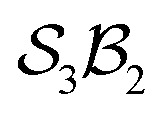
 system

Although the spatial constraints within the inner cavity of subporphyrins restrict the number of stable species that can form with hydrogen bonders, it is instructive to explore all theoretical possibilities as a basis for deeper understanding of the formalism. Larger macrocycles can readily accommodate multiple hydrogen bonders.

**Fig. 11:**
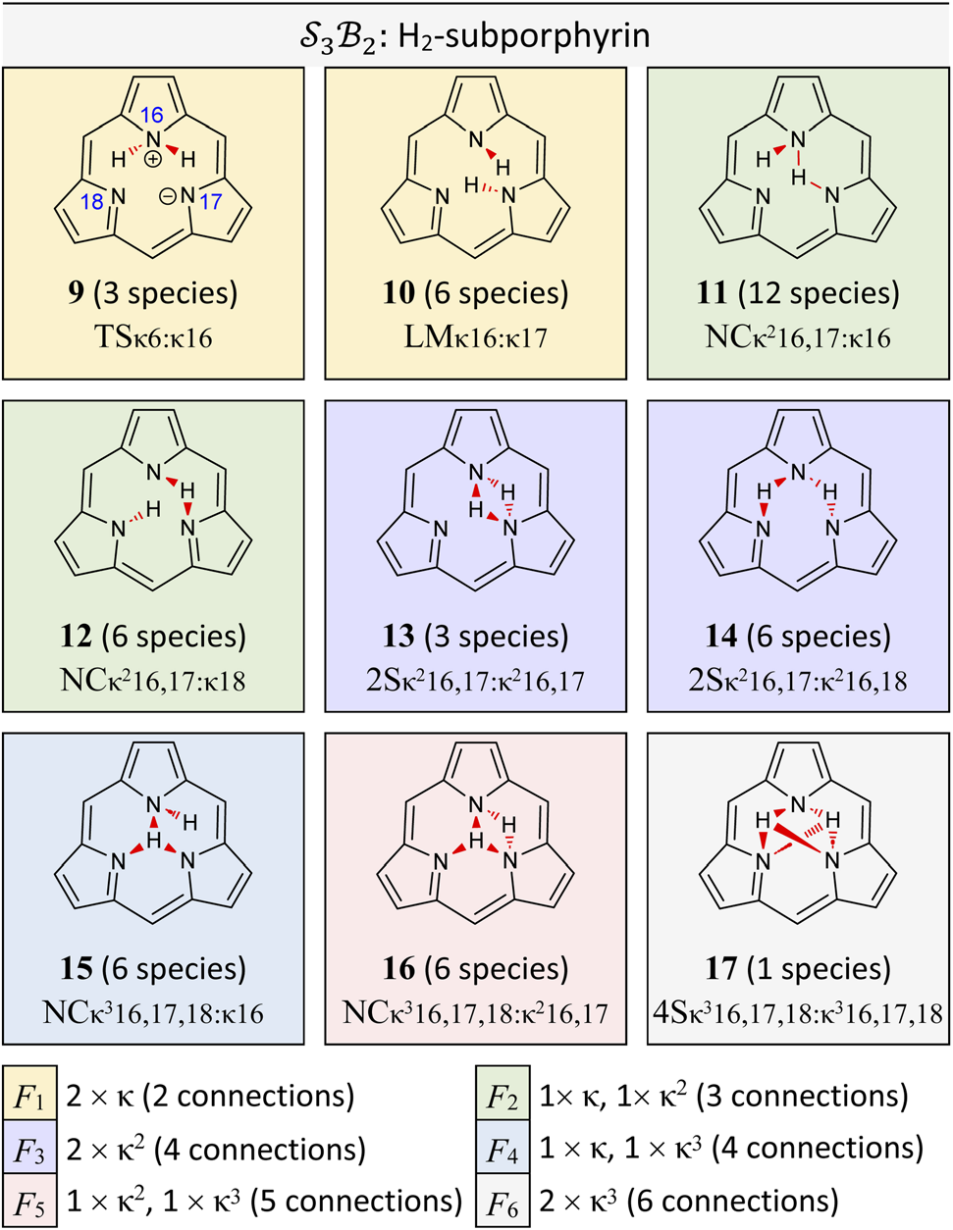
All genera for tautomerism in two 
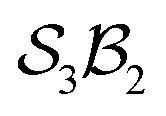
 systems (free-base subporphyrin and a general trioxido complex) exhibiting 
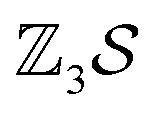
-symmetry under the condition of nondissociation. For each, all *N*_g_ = 9 possible genera are shown as 2D chemical structures. Genera are colour-coded into *N*_f_ = 6 families F_1_ through to F_6_, depicting different permuted connection totals and bonder linking patterns as described in the legend. Compact labels are given for a representative species of each free-base subporphyrin genus. Stereochemistries are only demonstrative.

Building on the earlier example of H-tautomerism in the free-base subporphyrin monoanion, the addition of a second bonder (H^+^) gives the neutral free-base subporphyrin, partitioned as 
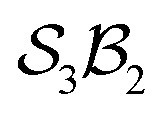
 (Fig. 11). In this case, two mobile H-atom bonders are free to migrate within the macrocyclic cavity. The files “genera_S3B2.txt” and “species_S3B2.txt” provided in the SI contain the raw data used to generate the generic and specific structures shown in Fig. 11.

The scope of possible atom-connectivity configurations increases substantially with the addition of a second bonder. Inspection of Tables 1–3 shows that the 
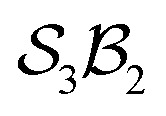
 class contains six families and 49 species, distributed among nine genera. Unlike the simple definitions of taxonomic ranks in the N-atom class (where all atoms are equivalent), the definitions of family and genus for 
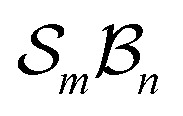
-partitioned classes with *n* ≥ 2 are more intricate.

Distinctions between genera within a single family are based on the combinations of bonder-site connection multiplicities. For example, within family F_1_, Genus 9 comprises species where both bonders attach to the same site, whereas Genus 10 comprises species where the bonders attach to different sites. In both cases, the bonders are non-bridging.

**Fig. 12:**
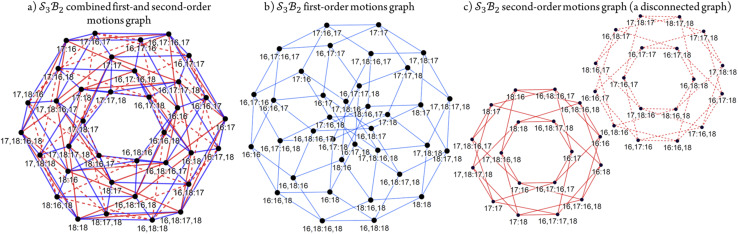
Toroidal desmotropic reaction graphs of genera 9–14 for the 
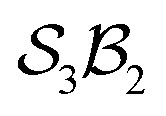
 class. All graph vertices are labeled by the bonder connectivity (omitting the κi symbols) and edges are coloured by motions type (blue: first order; red: second order). (a) Combined first- and second-order motions graph laid out to show the toroidal form. First-order and second-order motions indicated by blue and red graph edges, respectively. (b) First-order motions graph. (c) Second-order motions graph. This is a disconnected graph.

Graphical outputs from the accompanying software, applied to Genera 9–14 for the 
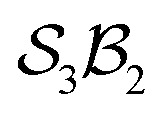
 class, are shown in Fig. 12. For clarity and to reduce complexity, For ease of visualisation, genera 15–17, which include at least one bonder connected to three sites, are omitted from this analysis.

The combined first-order and second-order motions graph for Genera 9–14 is shown in Fig. 12a, with first-order edges in blue and second-order edges in red. The topology of this combined graph corresponds to a 2-torus. It comprises 36 species (vertices) connected by 144 edges.

**Fig. 13:**
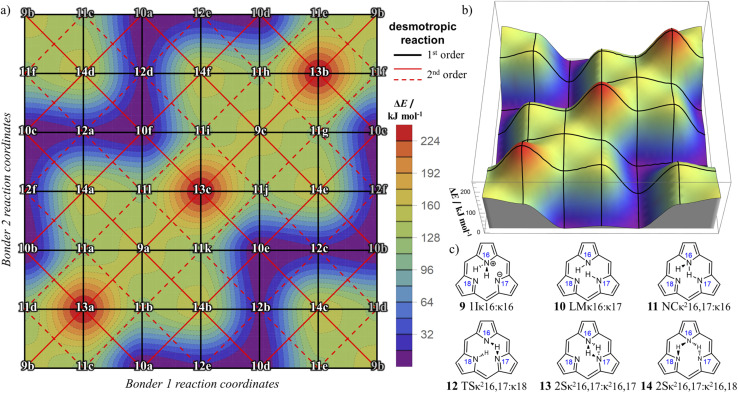
Interpolated approximation of the DFT-calculated potential-energy surface for tautomerism in free-base subporphyrin partitioned as 
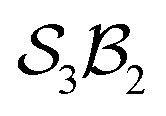
 using genera 9–14. Surface generated from calculated energies for representative structures for each genus. (a) 2D-periodic rendering of the 2-toroidal PES. All species located on the surface are labelled by their species number (see SI for details). The piecewise reaction coordinates of each bonder are periodic with replicate configuration genera shown in grey (top row and right edge). Desmotropic reactions are indicated as first order (solid black lines) and two second order networks (red dashed and red dotted lines). (b) 3D rendering of the PES. (c) The six genera with representative structures and their compact symbols indicating PES character. Stereochemistries are only demonstrative.

The first-order and second-order motion graphs (Fig. 12b and c, respectively) also exhibit 2-toroidal topology and, according to the Modular Structure Theorem, are subgraphs of the combined graph in Fig. 12a. The second-order graph (Fig. 12c) is disconnected, indicating that second-order motions alone cannot interconvert species between its separate subgraph components. The calculated PES corresponding to Fig. 12 is shown in Fig. 13 with further details in SI15 and SI16. Other examples of the 
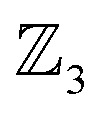
 symmetric 
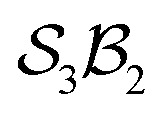
 class include free-base triphyrin[2.2.2] monoanion (*e.g.*, 28 less one inner H-atom). This expanded macrocycle admits larger atoms in place of the inner H-atoms and modulation of the corresponding PES (see Discussion).

### An 
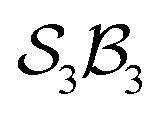
 system

The free-base triphyrin[2.2.2], which contains three bonders and three sites, corresponds to the 
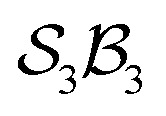
 class. Inspection of Tables 1–3 shows that this class comprises ten families, 23 genera, and 343 species, including configurations in which all three bonders attach to a single site atom (N-atom). On valence and steric grounds, such species are excluded from further analysis.

**Fig. 14:**
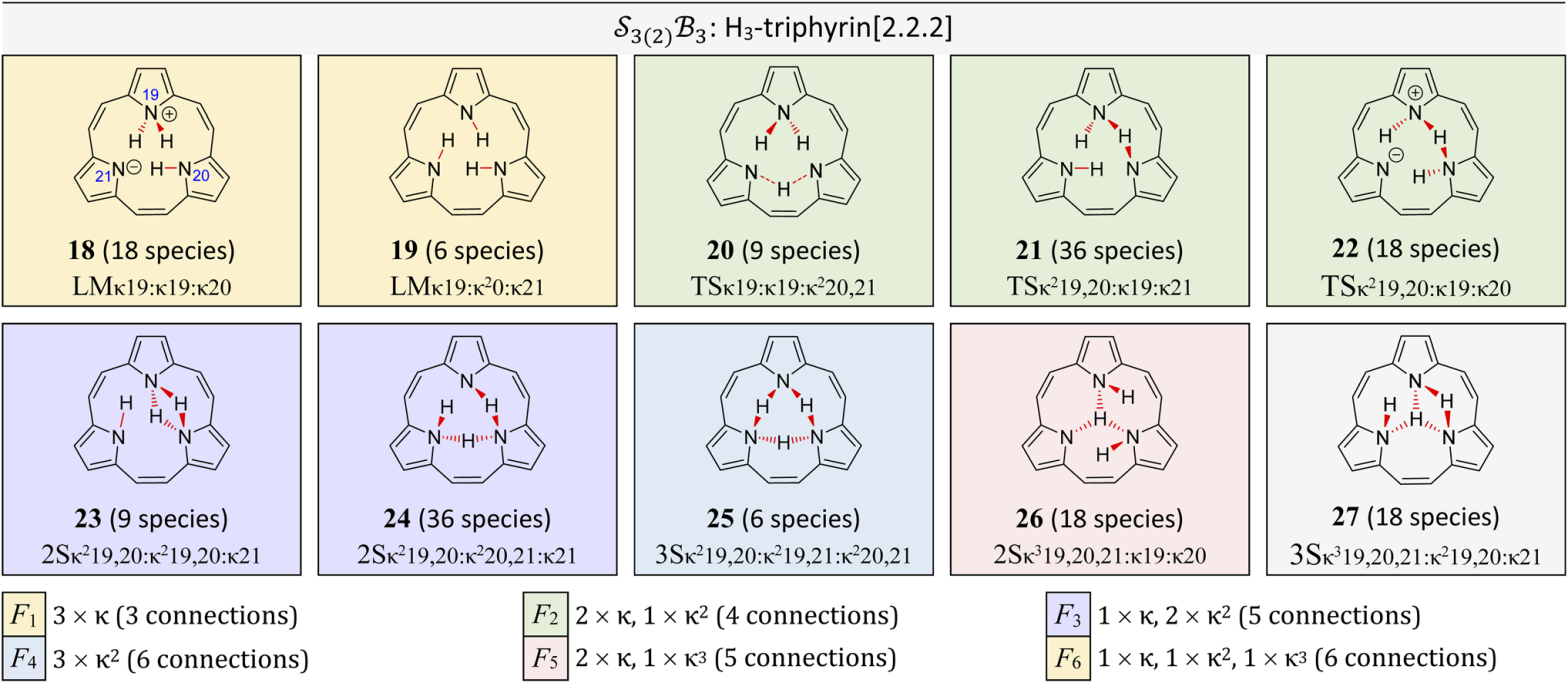
All bond topology genera for tautomerism in free-base triphyrin[2.2.2] corresponding to the 
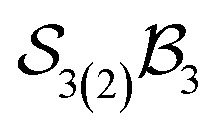
 class, exhibiting 
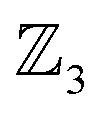

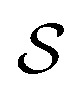
-symmetry, and under the condition of nondissociation. All *N*_g_ = 10 possible genera are shown as 2D chemical structures. The genera are color-coded into *N*_f_ = 6 families F_1_ through to F_6_, depicting different permuted bond totals and bonder linking patterns as described in the legend. The compact label is given for a representative species for each free-base triphyrin[2.2.2] genus. There are a total of 174 species. Stereochemistries are only demonstrative.

When the maximum number of bonders per site is restricted to 2, the system is denoted 
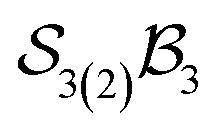
. Under this constraint, there are 6 families, 10 genera, and 174 species. The corresponding genera and families are shown in Fig. 14. Following the pattern established for the free-base subporphyrin monoanion (Fig. 7) and free-base subporphyrin (Fig. 11 and 12), the graphs for genera 18–25 exhibit the topology of a 3-torus, albeit with missing vertices arising from the exclusion of configurations in which three bonders occupy a single site.

Electronic data files describing the 
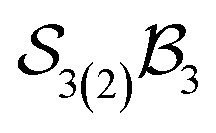
-partitioned free-base triphyrin[2.2.2] system are provided in the SI.

## Discussion – molecular constitution

### Integrability of the formalism with cheminformatic tools

In this work we have presented the Polytope Formalism of molecular constitution in a manner accessible to a broad cross-section of chemists, employing familiar structural depictions while reserving formal mathematics for the SI. Importantly, the matrix representation of the abstract polytopes used in this framework corresponds closely to the digital encoding of molecular structures. Consequently, we anticipate that the formalism can be seamlessly integrated with existing cheminformatic platforms, including those used in drug discovery and major databases such as Chemical Abstracts and Reaxys

### Nomenclature and non-local minimum structures on potential energy surfaces

Systematic chemical nomenclature can be regarded as a human-readable encoding of molecular graphs – or, equivalently, of their matrix representations.

The existing nomenclatural framework, however, is confined to chemical entities corresponding to local minima on potential energy surfaces. Transition-state structures and other non-minimum configurations cannot be named within this framework. Given their central role in elucidating or rationalising reaction mechanisms, in chemical education, and throughout the literature, this inability to systematically name and digitally store such species represents a significant limitation.

As demonstrated by examples in this paper, many species generated through the Polytope Formalism – and one of the keys to its power – correspond to the inclusion and description of non-local minima species. We contend that these entities should also be systematically nameable and storable within chemical databases. Only modest extensions to the current nomenclatural system are required – some we have already implemented and discussed in earlier work.^17–21^ In SI Section SI11, we adumbrate how atom-connectivity configurations are translated into this augmented nomenclatural framework. A detailed exposition will be presented in a forthcoming publication.

### Atom-connectivity analogues as structural motifs

At its most fundamental level, the Polytope Formalism of molecular constitution entails the combinatorial generation of atom-connectivity configurations. At this stage, no chemical elements are assigned to the abstract “atoms”. Although subsequent assignment of elements and calculation of representative structures yield system-specific geometries and energies, the underlying configurations and their interrelationships remain invariant – they are isomorphic regardless of atomic identity.

Consequently, the configuration and reaction graph of one chemical system can be directly mapped onto that of another analogous system: the absolute energies differ, but the topology of the PES remains identical. This gives rise to the concept of atom-connectivity analogues.

**Fig. 15:**
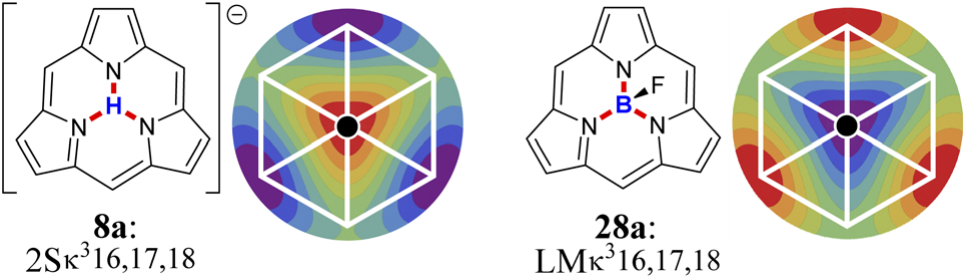
Demonstration of atom-connectivity configurations as structural motifs. Species 8a is the high energy central species (black) on its DFT-calculated PES. Its BF-analogue 37a becomes the lowest energy species on its PES. The reactions graphs (white) for both systems overlie their PESs and are isomorphic.

For example, in our earlier analysis of the free-base subporphyrin monoanion under 
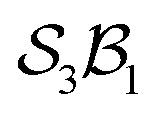
 partitioning (without bonder dissociation), we identified seven distinct species whose combined first- and second-order reaction graph exhibits a characteristic topology. A direct analogue of this system can be produced by replacing the bonder (H^+^) with [BF_2_]^+^, as illustrated in Fig. 15.

Fig. 15 shows that the reaction graphs of the two systems are isomorphic even though their PESs differ profoundly: species 8a is a high-energy second-order saddle point on its surface, whereas its analogue 28a is a local minimum and the global minimum of its PES.

Because the formalism systematically enumerates all possibilities under explicit constraints, it enables the discovery and classification of such structural motifs, clearly distinguishing what has been observed from what remains undiscovered. In forthcoming work, we analyse the porphyrin system under 
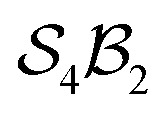
 partitioning (without bonder dissociation) and show that 12 of the 28 theoretically possible binding motifs have already been identified or tentatively proposed – though not always correctly.^21^

### The special cases of cycloadditions and chelotropic reactions

For concerted processes in which two or more atom connections form or break simultaneously – such as cycloadditions and chelotropic reactions – the list of atom-connectivity configurations generated by the Polytope Formalism does not distinguish between the cyclised product and the first-order transition state that leads to its formation.

**Fig. 16:**
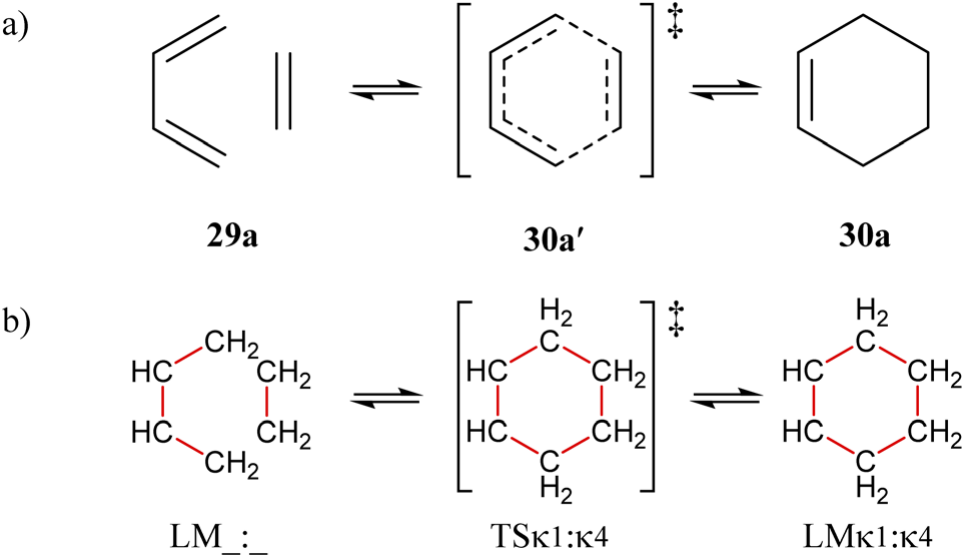
The [dienophile + diene] 29a undergoing [1,4]-cycloaddition to the adduct 30a. (a) Traditional depiction of concerted process including transition-state structure 30a′. (b) Atom-connectivity picture of the reaction does not differentiate between species 30a′ (TS^κ^1^:κ^4^^) and 30a (LM^κ^1^:κ^4^^).

An illustrative case is the Diels–Alder reaction (Fig. 16), where the transition state 30a′ and the product 30a share identical atom-connectivities. Consequently, in its simplest implementation and translation into 3D structures, the formalism under-samples the configuration space for such transformations.

As far as we can determine, this is the only scenario of PES under-sampling and there are two approaches that can be used to accommodate this. The first is simply to include the search for an intermediate species (*e.g.*, the TS 30a′) as suggested by the PES character of the two species (*e.g.*, both 29a and 30a are LM structures). This however represents an ad hoc solution, and we cannot guarantee it will always work. The better approach is to always sample two equally spaced points along every reaction coordinate when translating configurations into 3D representations. This guarantees a full sampling of the configuration space and has the added benefit of providing a finer representation and thus encoding of the associated PES. A full algorithmic description will be presented elsewhere.

Further details of this 
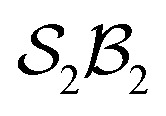
-partitioned system are provided in SI section SI18, where it also serves as an example of an 
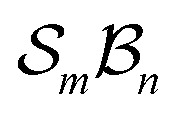
-partitioned bimolecular system (*i.e.*, one that allows bonder dissociation). Note the underscore placeholders in the symbol LM_:_ for 29a where “_” means no bonder-site atom-connections for that bonder.

### Reaction graph as a framework for multidimensional transition-state theory

When at-least energies – and ideally, gradients, and curvatures – are assigned to the vertices of a reaction graph, the resulting construct provides an efficient encoding of the associated PES since the Polytope Formalism samples its key stationary points. It follows that Transition-State Theory (TST) can be extended from individual reactions to complete reaction networks. The reaction graph, together with its energetic parameters, thus forms a framework for multidimensional TST from which a system of rate equations can be determined.

Beyond the microcanonical formulation, this multidimensional TST can be generalised to describe ensemble systems, enabling treatment of statistical-thermodynamic questions and a unified approach to kinetics across complex reaction networks.

### Implications of the modular structure theorem – nested spaces and self-similar topologies of subregions

The most significant implication of the Modular Structure Theorem lies in how it formalises relationships between analyses performed under different levels of constraint. Focussed analyses – obtained by imposing explicit restrictions – yield simpler, more tractable representations, and the theorem states that each such constrained analysis is nested within (*i.e.*, forms a subspace of) the corresponding unconstrained space.

The converse is also true: adding further atoms generates a larger system whose reaction graph contains – or embeds – that of the smaller system. The topologies that emerge therefore display self-similar and hierarchical organisation: simpler topologies are nested within more complex ones. The resulting “shape of Chemical Space” comprises recurring motifs extending across the broader structural landscape.

This nested hierarchy provides a mathematical rationale for the similar behaviour of chemical analogues and for the reproducibility of reaction outcomes under comparable conditions. Indeed, the entire logic of retrosynthetic analysis relies on this nested and self-similar organisation of Chemical Space.

### Exploiting the modular structure theorem for describing composite object interactions

Up to this point, our discussion has centred on the Polytope Formalism at the atomistic level. However, the same combinatorial and organisational principles can be applied to interactions between larger composite objects.

Examples include the thermodynamic behaviour of supramolecular assemblies^50–54^ and DNA,^55–57^ both of which can be viewed as comprising discrete components (*e.g.*, nucleobase sequences, polydentate ligands, metal ions) that interact through well-defined modes and constraints. For instance, on single-stranded DNA, intramolecular Watson–Crick H-bonding is only feasible when the paired nucleobases are separated by at least ∼3 nucleotides,^57^ providing a simple, physically motivated rule that excludes such impossible configurations. Within the formalism, these components generate a structured list of “species complexes”, each of which may subsequently be modelled at an atomistic level to determine their energetics.

The organisational machinery of the Polytope Formalism then arranges these complexes into a reaction landscape, with the modular structure theorem guaranteeing that this coarse-grained representation is a constrained subspace of the fully atomistic one. Thus, composite-level Chemical Space is revealed as a lower-resolution view of the same underlying structure, demonstrating the scalability of the formalism from atoms to supramolecular systems.

### Additional input needed to describe a chemical system

Practitioners of quantum-chemical modelling recognise that, beyond approximate molecular geometry, additional explicit and implicit parameters are required to specify a chemical system. The most common case involves an electronic ground state – typically a singlet for closed-shell systems or a doublet for open-shell systems – together with the lowest rovibrational state. More elaborate descriptions may further specify isotopic composition (*e.g.*, ^2^H or ^13^C in place of ^1^H and ^12^C, respectively), overall nuclear spin state, or excited electronic and vibrational states. Each of these factors is essential for a complete physical description.

In the present work we have focussed on molecular constitution, which concerns atom connectivity and, indirectly, atom–atom distances. Many of the generated species are chiral, and stereochemical distinctions must therefore be incorporated when conceptualising or modelling these entities. This naturally raises a final question: when all such details are defined, does this fully describe a chemical entity? We contend that the answer is yes, and in the following section we outline what such a comprehensive description entails.

## Discussion – the general framework of the Polytope Formalism and its full application

### High-level structure of the Polytope Formalism

In developing the conceptual and organisational framework of the Polytope Formalism for the seemingly disparate domains of stereoisomerism and molecular constitution, we have presented applications encompassing both continuum features (*e.g.* spatial relationships) and discrete properties (*e.g.* atom connectivity). We have further shown that the same mathematical tools – particularly Combinatorics and Graph Theory – can be applied to comprehensively generate and systematically organise the complete set of possible configurations.

We now turn to the additional factors that define chemical entities and consider how, at the highest level, the conceptual and organisational framework of the Polytope Formalism may be extended to incorporate them.

**Fig. 17:**
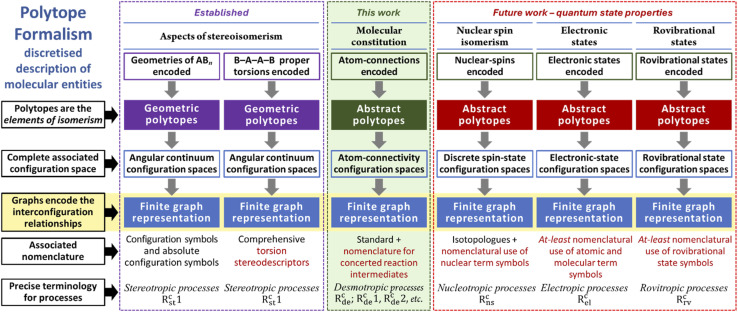
Overview of the Polytope Formalism description of molecular entities. The general framework is given on the left with specific details for each aspect of molecular state. The items in red require development.

Fig. 17 presents, side by side, the established aspects of stereoisomerism within the formalism^20^ and how molecular constitution (this work) fits within this higher-level structure. For stereoisomerism, the associated configurations correspond to geometric polytopes, and the relevant configuration spaces are continua that can subsequently be discretised. In contrast, as shown in this paper, molecular constitution involves atom-connectivity configurations that are abstract polytopes with inherently discrete configuration spaces.

For both stereoisomerism and molecular constitution, each has an associated nomenclatural system and a precise terminological lexicon.

Together, stereoisomerism and molecular constitution describe the essential facets of molecular geometry. Mechanistically, processes associated with unimolecular stereoisomerism (stereotropic) are driven primarily by changes in angular internal coordinates, with atom–atom distances remaining approximately constant – the signature of preserved connectivity.^19,20^ By contrast, changes in molecular constitution (desmotropic) involve vibrational modes dominated by bond-stretching character, although angular contributions are typically present except in the simplest systems.

It is important to note that within this framework, bond length as a geometric parameter is not used directly. Consequently, the Polytope Formalism does not seek to provide an alternative molecular coordinate system in 
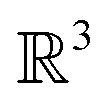
. In the formalism's application to stereoisomerism, the discretisation step transformed continua real-space parameters into more abstract discrete spaces that cease to represent real-space parameters. In a sense, with molecular constitution the picture is the other way around. Its implementation begins with discrete parameters (whether an atom–atom connection exists or not) and only when translating this into a 3D embodiment does bond-length enter the picture.

### The other chemical descriptors

From current understanding, all remaining factors that define chemical entities are quantum-mechanical in nature – specifically, the nuclear details, electronic configuration, and rovibrational state of the system. Our proposal for how each of these components fits within the higher-level structure of the Polytope Formalism is illustrated in Fig. 17.

Nuclear details determine isotopologues and spin isomers. Because the sets of possibilities arising from such nuclear variations are finite, their permutation is relatively straightforward and, as with molecular constitution, the corresponding “polytopes” representing nuclear configurations are abstract. Interconversion of isotopologues within a chemical system (which need not be unimolecular) occurs only through nuclear transpositions, giving rise to simple isotopologue exchange graphs that represent the configuration spaces. In the case of nuclear-spin isomerism, quantum mechanics dictates a more complex range of possibilities. Although in both cases the associated energy landscapes span comparatively small ranges relative to typical chemical energies, this landscape of nuclear spin states is probed – *via* nucleotropic processes – during NMR measurements.

Existing systematic nomenclature already accommodates isotopologues; the inclusion of nuclear term symbols within that framework would render it complete. The electronic state of a molecular entity, not necessarily unimolecular, is a central determinant of chemical identity. It represents the specific configuration of electrons, defined by their allowed energy levels and spins for a fixed nuclear framework. The number of electrons and their permitted spin states form a finite set of possibilities, while the spectrum of energy levels is more complex, typically comprising a manifold of discrete states up to the onset of a continuum. Even so, it remains feasible to generate electronic-state configurations using a combinatorial approach. Again, these configurations can be represented using abstract polytopes though how the continuum is integrated with the discrete states requires careful consideration.

Electronic-state symbols capture the gross facets of electronic-state configurations but are not used within the existing nomenclatural system. Processes that change one electronic configuration to another, for example the absorption or emission of a photon, are described as electropic.

Rovibrational states can be treated analogously to electronic states, again using abstract polytopes to represent their possible configurations. Given the relative energy scales involved, the vibrational and rotational components can be considered separately or jointly. Consistent with established quantum-chemical practice, it is natural to treat electronic and rovibrational states together within a unified framework that reflects the full manifold of electronic states, each of which accommodates a manifold of vibrational states which, in turn, accommodate the rotational states. A nomenclatural system for these rovibrational aspects requires much work as it would need to accommodate both vertical transitions and relaxational mechanisms that span a wide range of time scales. Processes that interconvert rovibrational states, for example collisions in a bimolecular system or the absorption/emission of a photon, are described as rovitropic.

### Chemical space

Unless new physical principles governing chemistry are discovered, the assignment of all essential descriptors – atom connectivity, stereochemical configuration, nuclear details, electronic state, and rovibronic-state configurations – constitutes a complete system of discretised chemical definition. Applying all these descriptors within a Polytopal Formalism analysis enables the comprehensive generation of the associated Chemical Space.

In broad terms, each configuration is associated with a specific energy (config, *E*), and thus an energy term corresponds to every point in the discretised configuration space. Every physical or chemical process may therefore be viewed as a “path” or, more precisely, a series of steps, through this space. For example, in photocatalysed synthesis,^54,58^ a reaction can be conceptualised as a sequence of discrete steps through this Chemical Space – beginning with vertical excitation, followed by a shift in overall electronic configuration as the excitation is transferred to the reactants, and concluding with a rearrangement of atom connectivities to yield the products.

The formally complete mathematical definition provided by the Polytope Formalism facilitates computational exploration of this landscape. Although infinite in dimension and complexity, the hierarchically structured and self-similar shape of this space promises its tractable exploration. Chemists have long invoked the notion of Chemical Space in a heuristic or metaphorical sense; the Polytope Formalism provides it with a physically grounded, mathematically rigorous, granular structure. Within this framework, topology defines the adjacency relations between species, while topography – the associated energetic contours – reflects the physical reality of the chemical landscape. In a real sense, all chemistry is an exploration of Chemical Space and the Polytope Formalism provides the roadmap for its systematic exploration.

## Conclusions

The Polytope Formalism provides a rigorous and unifying mathematical framework for representing all possible molecular configurations and their relationships. Put simply, by generating a description for every possibility and mechanism, it not only describes what has already been discovered, but points the way to the undiscovered. Extending it from stereoisomerism to molecular constitution shows that both arise naturally within the same abstract framework and yielding analogously structured hierarchical spaces. By describing molecules solely through atom connectivity, the formalism captures traditional isomers as well as subvalent and hypervalent species representing reaction-intermediate species, giving a complete picture of interconversion processes.

When energy details are assigned to the vertices of its reaction graphs, the formalism becomes a compact encoding of the potential-energy surface (PES), linking configuration topology directly with chemical reactivity. Through focussed analyses, such as the 
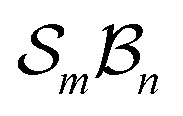
-partitioning approach, tractable subsets of this space can be explored systematically, revealing reaction pathways and relationships among species with mathematical completeness and chemical clarity.

In outlook, the Polytope Formalism promises a single unifying conceptual and organisational framework for accommodating all of chemistry. Further, it reveals how chemical terminology, nomenclature, and digital representation of chemical entities can be expanded thus facilitating and empowering research approaches beyond what is currently possible. Finally, as computational methods and resources continue to expand, the Polytope Formalism offers a natural mathematical infrastructure for the automated exploration of Chemical Space – linking rigorous theory, digital chemistry, and data-driven discovery within a single coherent and universal framework.

## Author contributions

Both authors made substantive contributions to the work reported herein and to preparation of the manuscript. All computing and the development of the interactive software and numerous resource files was carried out by PJC.

## Conflicts of interest

There are no conflicts to declare.

## Note added after first publication

This article replaces the version published on 8th January 2026. Fig. 12 image size has been corrected.

## Abbreviations

Class: highest-level organisational taxon
Desmotropic: of or relating to a change in atom connectivity in a molecular entity
DFT: Density-Functional Theory
Electropic: of or relating to a change in the electronic configuration of a molecular entity
Family: second-level organisational taxon
Genus: second lowest-level organisational taxon
MEP: minimum-energy pathway
Nucleotropic: of or relating to a change in the nuclear details (isotopes, nuclear state, molecular nuclear state) in a molecular entity
PES: potential energy surface
*R* ^c^_de_
: rearrangement concerted desmotropic
Rovitropic: of or relating to a change in the vibrational and/or rotational state of a molecular entity
SI: supplementary information

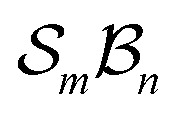

the *m*-site, *n*-bonder partitioned class

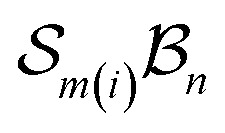

the *m*-site, *n*-bonder partitioned class with a maximum of *i*-bonders at a site
Species: lowest-level organisational taxon
Stereotropic: of or relating to a change in spatial arrangements of atoms in a molecular entity without changing the atom connectivity
TST: Transition-State Theory

## Supplementary Material

SC-OLF-D5SC08813E-s001

SC-OLF-D5SC08813E-s002

## Data Availability

The data supporting this article have been included as part of the supplementary information (SI) and numerous interactive software and resource files are included. Further detailed information including formal definitions and Cartesian coordinates of modelled species (PDF) also included. Supplementary information: further discussion, detailed mathematics, software, and other resource files: (i) “genus_direct.for” software for generating genera and species given an 
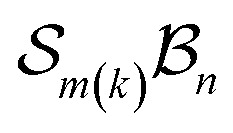
 class; (ii) many species and genus files included; (iii) mathematica notebooks for N-atom class listing, configuration space graphing, motions-order calculations, and interactive free-base subporphyrin PES viewer; (iv) output files from the graphing software for the 
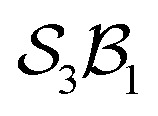
, 
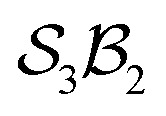
, and 
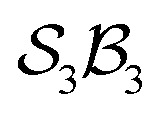
 classes. See DOI: https://doi.org/10.1039/d5sc08813e.
