# Supplementary material for: The Polytope Formalism: application to molecular constitution and the prospect of a complete description of Chemical Space
Source: Chem Sci. 2026 Jan 8;17(4):2102–18. doi: 10.1039/d5sc08813e (PMC12780917; doi:10.1039/d5sc08813e)

Class: S2B2 with site symmetry point group D2h

Genera included = {1, 2, 3, 4, 5, 6}

1st and 2nd order motions graph:

Graph vertex layout: SpringElectricalEmbedding

Hamiltonian graph? True

Eulerian graph? True

Planar graph? False

Graph radius = 2.

Graph diameter = 2.

Graph density = 0.571429

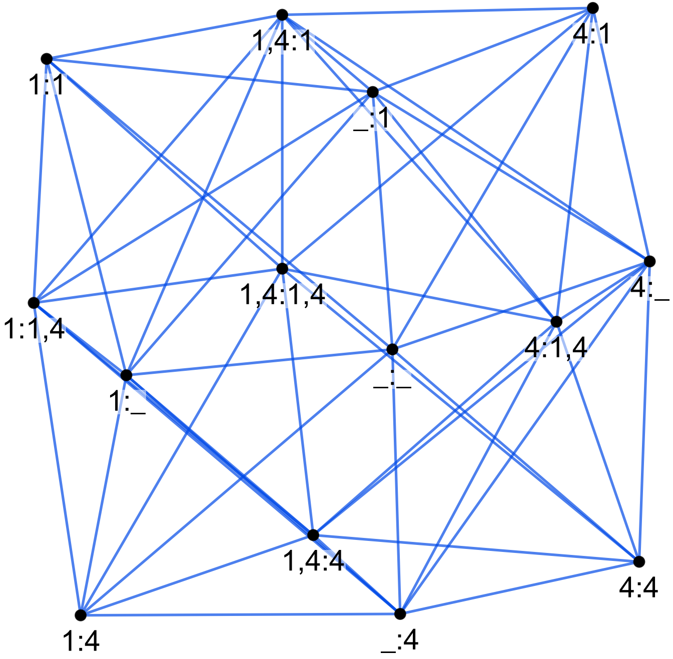

Supplement: SC-017-D5SC08813E-s001 [file SC-017-D5SC08813E-s001.zip › publication files/graphing outputs/cyclo - addition S2B2_S2B2_genera{1, 2, 3, 4, 5, 6}_1_2.pdf]
