# Supplementary material for: The Polytope Formalism: application to molecular constitution and the prospect of a complete description of Chemical Space
Source: Chem Sci. 2026 Jan 8;17(4):2102–18. doi: 10.1039/d5sc08813e (PMC12780917; doi:10.1039/d5sc08813e)

Class: S2B2 with site symmetry point group D2h
Genera included = {1, 2, 3, 4, 5, 6}
2nd order motions graph:
Graph vertex layout: SpringElectricalEmbedding
Hamiltonian graph? False
Eulerian graph? False
Planar graph? False
Graph radius = Infinity
Graph diameter = Infinity
Graph density = 0.307692

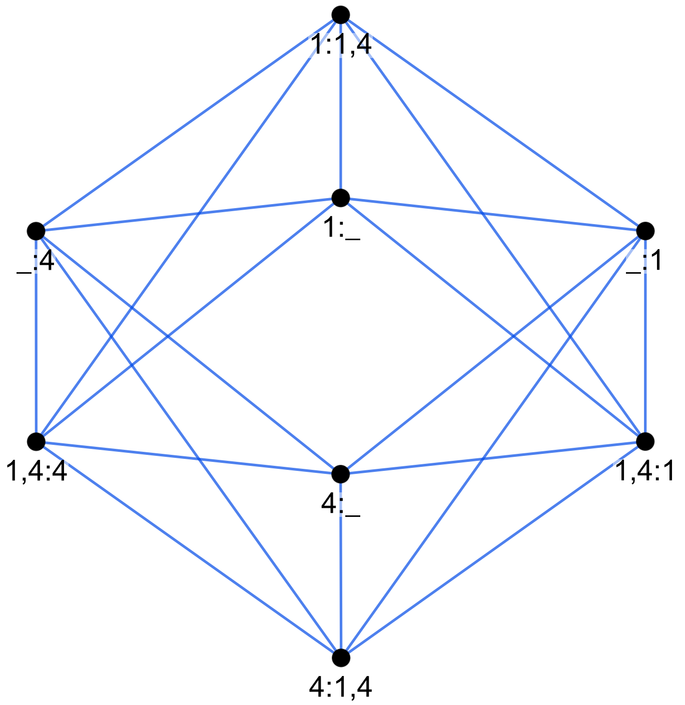

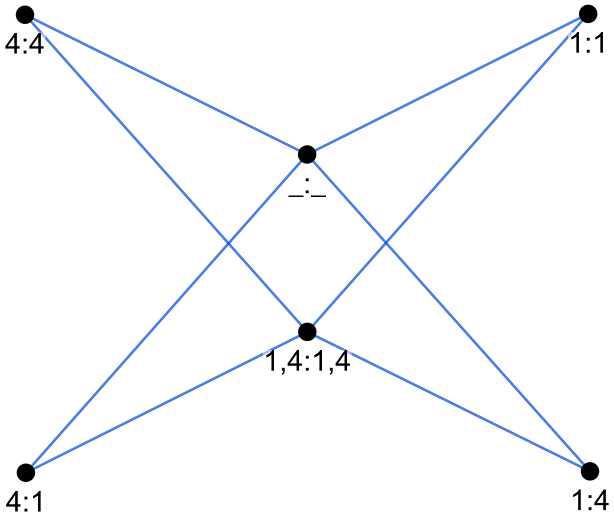

Supplement: SC-017-D5SC08813E-s001 [file SC-017-D5SC08813E-s001.zip › publication files/graphing outputs/cyclo - addition S2B2_S2B2_genera{1, 2, 3, 4, 5, 6}_2.pdf]
