# Supplementary material for: The Polytope Formalism: application to molecular constitution and the prospect of a complete description of Chemical Space
Source: Chem Sci. 2026 Jan 8;17(4):2102–18. doi: 10.1039/d5sc08813e (PMC12780917; doi:10.1039/d5sc08813e)
Supplement: SC-017-D5SC08813E-s001 [file SC-017-D5SC08813E-s001.zip › publication files/motions order outputs/S2B3 motions order table.pdf]

	0:0:0	1:1:1	0:0:1	0:1:0	1:0:0	1:1:0	1:0:1	0:1:1	0:0:0,1,2	0:0,1,2:0	0,1,2:0:0	1:1:0,1,2	1:0,1,2:1	0,1,2:1:1	0:1:0,1,2	0:0,1,2:1	1:0:0,1,2	1:0,1,2:0	0,1,2:0:1	0,1,2:1:0	0:0,1,2:0,1,2	0,1,2:0:0,1,2	0,1,2:0,1,2:0	1:0,1,2:0,1,2	0,1,2:1:0,1,2	0,1,2:0,1,2:1	0,1,2:0,1,2:0,1,2
0:0:0	0	–	–	–	–	–	–	–	2	2	2	–	–	–	–	–	–	–	–	–	4	4	4	–	–	–	6
1:1:1	–	0	–	–	–	–	–	–	–	–	–	2	2	2	–	–	–	–	–	–	–	–	–	4	4	4	6
0:0:1	–	–	0	–	–	–	–	–	2	–	–	–	–	–	–	2	–	–	2	–	4	4	–	–	–	4	6
0:1:0	–	–	–	0	–	–	–	–	–	2	–	–	–	–	2	–	–	–	–	2	4	–	4	–	4	–	6
1:0:0	–	–	–	–	0	–	–	–	–	–	2	–	–	–	–	–	2	2	–	–	–	4	4	4	–	–	6
1:1:0	–	–	–	–	–	0	–	–	–	–	–	2	–	–	–	–	–	2	–	2	–	–	4	4	4	–	6
1:0:1	–	–	–	–	–	–	0	–	–	–	–	–	2	–	–	–	2	–	2	–	–	4	–	4	–	4	6
0:1:1	–	–	–	–	–	–	–	0	–	–	–	–	–	2	2	2	–	–	–	–	4	–	–	–	4	4	6
0:0:0,1,2	2	–	2	–	–	–	–	–	0	4	4	–	–	–	–	4	–	–	4	–	2	2	6	–	–	6	4
0:0,1,2:0	2	–	–	2	–	–	–	–	4	0	4	–	–	–	4	–	–	–	–	4	2	6	2	–	6	–	4
0,1,2:0:0	2	–	–	–	2	–	–	–	4	4	0	–	–	–	–	–	4	4	–	–	6	2	2	6	–	–	4
1:1:0,1,2	–	2	–	–	–	2	–	–	–	–	–	0	4	4	–	–	–	4	–	4	–	–	6	2	2	6	4
1:0,1,2:1	–	2	–	–	–	–	2	–	–	–	–	4	0	4	–	–	4	–	4	–	–	6	–	2	6	2	4
0,1,2:1:1	–	2	–	–	–	–	–	2	–	–	–	4	4	0	4	4	–	–	–	–	6	–	–	6	2	2	4
0:1:0,1,2	–	–	–	2	–	–	–	2	–	4	–	–	–	4	0	4	–	–	–	4	2	–	6	–	2	6	4
0:0,1,2:1	–	–	2	–	–	–	–	2	4	–	–	–	–	4	4	0	–	–	4	–	2	6	–	–	6	2	4
1:0:0,1,2	–	–	–	–	2	–	2	–	–	–	4	–	4	–	–	–	0	4	4	–	–	2	6	2	–	6	4
1:0,1,2:0	–	–	–	–	2	2	–	–	–	–	4	4	–	–	–	–	4	0	–	4	–	6	2	2	6	–	4
0,1,2:0:1	–	–	2	–	–	–	2	–	4	–	–	–	4	–	–	4	4	–	0	–	6	2	–	6	–	2	4
0,1,2:1:0	–	–	–	2	–	2	–	–	–	4	–	4	–	–	4	–	–	4	–	0	6	–	2	6	2	–	4
0:0,1,2:0,1,2	4	–	4	4	–	–	–	4	2	2	6	–	–	6	2	2	–	–	6	6	0	4	4	–	4	4	2
0,1,2:0:0,1,2	4	–	4	–	4	–	4	–	2	6	2	–	6	–	–	6	2	6	2	–	4	0	4	4	–	4	2
0,1,2:0,1,2:0	4	–	–	4	4	4	–	–	6	2	2	6	–	–	6	–	6	2	–	2	4	4	0	4	4	–	2
1:0,1,2:0,1,2	–	4	–	–	4	4	4	–	–	–	6	2	2	6	–	–	2	2	6	6	–	4	4	0	4	4	2
0,1,2:1:0,1,2	–	4	–	4	–	4	–	4	–	6	–	2	6	2	2	6	–	6	–	2	4	–	4	4	0	4	2
0,1,2:0,1,2:1	–	4	4	–	–	–	4	4	6	–	–	6	2	2	6	2	6	–	2	–	4	4	–	4	4	0	2
0,1,2:0,1,2:0,1,2	6	6	6	6	6	6	6	6	4	4	4	4	4	4	4	4	4	4	4	4	2	2	2	2	2	2	0
