# Supplementary material for: The Polytope Formalism: application to molecular constitution and the prospect of a complete description of Chemical Space
Source: Chem Sci. 2026 Jan 8;17(4):2102–18. doi: 10.1039/d5sc08813e (PMC12780917; doi:10.1039/d5sc08813e)
Supplement: SC-017-D5SC08813E-s001 [file SC-017-D5SC08813E-s001.zip › publication files/motions order outputs/S3B2 motions order table.pdf]

	0:0	1:1	2:2	0:1	1:0	1:2	2:1	2:0	0:2	0:1,2	1,2:0	1:0,2	0,2:1	2:0,1	0,1:2	0:0,2	0,2:0	1:0,1	0,1:1	2:1,2	1,2:2	0:0,1	0,1:0	1:1,2	1,2:1	2:0,2	0,2:2	1,2:1,2	0,2:0,2	0,1:0,1	1,2:0,2	0,2:1,2	0,2:0,1	0,1:0,2	0,1:1,2	1,2:0,1	0:0,1,2	0,1,2:0	1:0,1,2	0,1,2:1	2:0,1,2	0,1,2:2	1,2:0,1,2	0,1,2:1,2	0,2:0,1,2	0,1,2:0,2	0,1:0,1,2	0,1,2:0,1	0,1,2:0,1,2
0:0	0	–	–	–	–	–	–	–	–	–	–	–	–	–	–	1	1	–	–	–	–	1	1	–	–	–	–	–	2	2	–	–	2	2	–	–	2	2	–	–	–	–	–	–	3	3	3	3	4
1:1	–	0	–	–	–	–	–	–	–	–	–	–	–	–	–	–	–	1	1	–	–	–	–	1	1	–	–	2	–	2	–	–	–	–	2	2	–	–	2	2	–	–	3	3	–	–	3	3	4
2:2	–	–	0	–	–	–	–	–	–	–	–	–	–	–	–	–	–	–	–	1	1	–	–	–	–	1	1	2	2	–	2	2	–	–	–	–	–	–	–	–	2	2	3	3	3	3	–	–	4
0:1	–	–	–	0	–	–	–	–	–	1	–	–	1	–	–	–	–	–	1	–	–	1	–	–	–	–	–	–	–	2	–	2	2	–	2	–	2	–	–	2	–	–	–	3	3	–	3	3	4
1:0	–	–	–	–	0	–	–	–	–	–	1	1	–	–	–	–	–	1	–	–	–	–	1	–	–	–	–	–	–	2	2	–	–	2	–	2	–	2	2	–	–	–	3	–	–	3	3	3	4
1:2	–	–	–	–	–	0	–	–	–	–	–	1	–	–	1	–	–	–	–	–	1	–	–	1	–	–	–	2	–	–	2	–	–	2	2	–	–	–	2	–	–	2	3	3	–	3	3	–	4
2:1	–	–	–	–	–	–	0	–	–	–	–	–	1	1	–	–	–	–	–	1	–	–	–	–	1	–	–	2	–	–	–	2	2	–	–	2	–	–	–	2	2	–	3	3	3	–	–	3	4
2:0	–	–	–	–	–	–	–	0	–	–	1	–	–	1	–	–	1	–	–	–	–	–	–	–	–	1	–	–	2	–	2	–	2	–	–	2	–	2	–	–	2	–	3	–	3	3	–	3	4
0:2	–	–	–	–	–	–	–	–	0	1	–	–	–	–	1	1	–	–	–	–	–	–	–	–	–	–	1	–	2	–	–	2	–	2	2	–	2	–	–	–	–	2	–	3	3	3	3	–	4
0:1,2	–	–	–	1	–	–	–	–	1	0	–	–	2	–	2	–	–	–	2	–	–	–	–	–	–	–	2	–	–	–	–	1	–	–	1	–	1	–	–	3	–	3	–	2	2	–	2	–	3
1,2:0	–	–	–	–	1	–	–	1	–	–	0	2	–	2	–	–	–	2	–	–	–	–	–	–	–	2	–	–	–	–	1	–	–	–	–	1	–	1	3	–	3	–	2	–	–	2	–	2	3
1:0,2	–	–	–	–	1	1	–	–	–	–	2	0	–	–	2	–	–	–	–	–	2	–	2	–	–	–	–	–	–	–	1	–	–	1	–	–	–	3	1	–	–	3	2	–	–	2	2	–	3
0,2:1	–	–	–	1	–	–	1	–	–	2	–	–	0	2	–	–	–	–	–	2	–	2	–	–	–	–	–	–	–	–	–	1	1	–	–	–	3	–	–	1	3	–	–	2	2	–	–	2	3
2:0,1	–	–	–	–	–	–	1	1	–	–	2	–	2	0	–	–	2	–	–	–	–	–	–	–	2	–	–	–	–	–	–	–	1	–	–	1	–	3	–	3	1	–	2	–	2	–	–	2	3
0,1:2	–	–	–	–	–	1	–	–	1	2	–	2	–	–	0	2	–	–	–	–	–	–	–	2	–	–	–	–	–	–	–	–	–	1	1	–	3	–	3	–	–	1	–	2	–	2	2	–	3
0:0,2	1	–	–	–	–	–	–	–	1	–	–	–	–	–	2	0	2	–	–	–	–	–	2	–	–	–	2	–	1	–	–	–	–	1	–	–	1	3	–	–	–	3	–	–	2	2	2	–	3
0,2:0	1	–	–	–	–	–	–	1	–	–	–	–	–	2	–	2	0	–	–	–	–	2	–	–	–	2	–	–	1	–	–	–	1	–	–	–	3	1	–	–	3	–	–	–	2	2	–	2	3
1:0,1	–	1	–	–	1	–	–	–	–	–	2	–	–	–	–	–	–	0	2	–	–	–	2	–	2	–	–	–	–	1	–	–	–	–	–	1	–	3	1	3	–	–	2	–	–	–	2	2	3
0,1:1	–	1	–	1	–	–	–	–	–	2	–	–	–	–	–	–	–	2	0	–	–	2	–	2	–	–	–	–	–	1	–	–	–	–	1	–	3	–	3	1	–	–	–	2	–	–	2	2	3
2:1,2	–	–	1	–	–	–	1	–	–	–	–	–	2	–	–	–	–	–	–	0	2	–	–	–	2	–	2	1	–	–	–	1	–	–	–	–	–	–	–	3	1	3	2	2	2	–	–	–	3
1,2:2	–	–	1	–	–	1	–	–	–	–	–	2	–	–	–	–	–	–	–	2	0	–	–	2	–	2	–	1	–	–	1	–	–	–	–	–	–	–	3	–	3	1	2	2	–	2	–	–	3
0:0,1	1	–	–	1	–	–	–	–	–	–	–	–	2	–	–	–	2	–	2	–	–	0	2	–	–	–	–	–	–	1	–	–	1	–	–	–	1	3	–	3	–	–	–	–	2	–	2	2	3
0,1:0	1	–	–	–	1	–	–	–	–	–	–	2	–	–	–	2	–	2	–	–	–	2	0	–	–	–	–	–	–	1	–	–	–	1	–	–	3	1	3	–	–	–	–	–	–	2	2	2	3
1:1,2	–	1	–	–	–	1	–	–	–	–	–	–	–	–	2	–	–	–	2	–	2	–	–	0	2	–	–	1	–	–	–	–	–	–	1	–	–	–	1	3	–	3	2	2	–	–	2	–	3
1,2:1	–	1	–	–	–	–	1	–	–	–	–	–	–	2	–	–	–	2	–	2	–	–	–	2	0	–	–	1	–	–	–	–	–	–	–	1	–	–	3	1	3	–	2	2	–	–	–	2	3
2:0,2	–	–	1	–	–	–	–	1	–	–	2	–	–	–	–	–	2	–	–	–	2	–	–	–	–	0	2	–	1	–	1	–	–	–	–	–	–	3	–	–	1	3	2	–	2	2	–	–	3
0,2:2	–	–	1	–	–	–	–	–	1	2	–	–	–	–	–	2	–	–	–	2	–	–	–	–	–	2	0	–	1	–	–	1	–	–	–	–	3	–	–	–	3	1	–	2	2	2	–	–	3
1,2:1,2	–	2	2	–	–	2	2	–	–	–	–	–	–	–	–	–	–	–	–	1	1	–	–	1	1	–	–	0	–	–	–	–	–	–	–	–	–	–	2	2	2	2	1	1	–	–	–	–	2
0,2:0,2	2	–	2	–	–	–	–	2	2	–	–	–	–	–	–	1	1	–	–	–	–	–	–	–	–	1	1	–	0	–	–	–	–	–	–	–	2	2	–	–	2	2	–	–	1	1	–	–	2
0,1:0,1	2	2	–	2	2	–	–	–	–	–	–	–	–	–	–	–	–	1	1	–	–	1	1	–	–	–	–	–	–	0	–	–	–	–	–	–	2	2	2	2	–	–	–	–	–	–	1	1	2
1,2:0,2	–	–	2	–	2	2	–	2	–	–	1	1	–	–	–	–	–	–	–	–	1	–	–	–	–	1	–	–	–	–	0	–	–	–	–	–	–	2	2	–	2	2	1	–	–	1	–	–	2
0,2:1,2	–	–	2	2	–	–	2	–	2	1	–	–	1	–	–	–	–	–	–	1	–	–	–	–	–	–	1	–	–	–	–	0	–	–	–	–	2	–	–	2	2	2	–	1	1	–	–	–	2
0,2:0,1	2	–	–	2	–	–	2	2	–	–	–	–	1	1	–	–	1	–	–	–	–	1	–	–	–	–	–	–	–	–	–	–	0	–	–	–	2	2	–	2	2	–	–	–	1	–	–	1	2
0,1:0,2	2	–	–	–	2	2	–	–	2	–	–	1	–	–	1	1	–	–	–	–	–	–	1	–	–	–	–	–	–	–	–	–	–	0	–	–	2	2	2	–	–	2	–	–	–	1	1	–	2
0,1:1,2	–	2	–	2	–	2	–	–	2	1	–	–	–	–	1	–	–	–	1	–	–	–	–	1	–	–	–	–	–	–	–	–	–	–	0	–	2	–	2	2	–	2	–	1	–	–	1	–	2
1,2:0,1	–	2	–	–	2	–	2	2	–	–	1	–	–	1	–	–	–	1	–	–	–	–	–	–	1	–	–	–	–	–	–	–	–	–	–	0	–	2	2	2	2	–	1	–	–	–	–	1	2
0:0,1,2	2	–	–	2	–	–	–	–	2	1	–	–	3	–	3	1	3	–	3	–	–	1	3	–	–	–	3	–	2	2	–	2	2	2	2	–	0	4	–	4	–	4	–	3	1	3	1	3	2
0,1,2:0	2	–	–	–	2	–	–	2	–	–	1	3	–	3	–	3	1	3	–	–	–	3	1	–	–	3	–	–	2	2	2	–	2	2	–	2	4	0	4	–	4	–	3	–	3	1	3	1	2
1:0,1,2	–	2	–	–	2	2	–	–	–	–	3	1	–	–	3	–	–	1	3	–	3	–	3	1	3	–	–	2	–	2	2	–	–	2	2	2	–	4	0	4	–	4	1	3	–	3	1	3	2
0,1,2:1	–	2	–	2	–	–	2	–	–	3	–	–	1	3	–	–	–	3	1	3	–	3	–	3	1	–	–	2	–	2	–	2	2	–	2	2	4	–	4	0	4	–	3	1	3	–	3	1	2
2:0,1,2	–	–	2	–	–	–	2	2	–	–	3	–	3	1	–	–	3	–	–	1	3	–	–	–	3	1	3	2	2	–	2	2	2	–	–	2	–	4	–	4	0	4	1	3	1	3	–	3	2
0,1,2:2	–	–	2	–	–	2	–	–	2	3	–	3	–	–	1	3	–	–	–	3	1	–	–	3	–	3	1	2	2	–	2	2	–	2	2	–	4	–	4	–	4	0	3	1	3	1	3	–	2
1,2:0,1,2	–	3	3	–	3	3	3	3	–	–	2	2	–	2	–	–	–	2	–	2	2	–	–	2	2	2	–	1	–	–	1	–	–	–	–	1	–	3	1	3	1	3	0	2	–	2	–	2	1
0,1,2:1,2	–	3	3	3	–	3	3	–	3	2	–	–	2	–	2	–	–	–	2	2	2	–	–	2	2	–	2	1	–	–	–	1	–	–	1	–	3	–	3	1	3	1	2	0	2	–	2	–	1
0,2:0,1,2	3	–	3	3	–	–	3	3	3	2	–	–	2	2	–	2	2	–	–	2	–	2	–	–	–	2	2	–	1	–	–	1	1	–	–	–	1	3	–	3	1	3	–	2	0	2	–	2	1
0,1,2:0,2	3	–	3	–	3	3	–	3	3	–	2	2	–	–	2	2	2	–	–	–	2	–	2	–	–	2	2	–	1	–	1	–	–	1	–	–	3	1	3	–	3	1	2	–	2	0	2	–	1
0,1:0,1,2	3	3	–	3	3	3	–	–	3	2	–	2	–	–	2	2	–	2	2	–	–	2	2	2	–	–	–	–	–	1	–	–	–	1	1	–	1	3	1	3	–	3	–	2	–	2	0	2	1
0,1,2:0,1	3	3	–	3	3	–	3	3	–	–	2	–	2	2	–	–	2	2	2	–	–	2	2	–	2	–	–	–	–	1	–	–	1	–	–	1	3	1	3	1	3	–	2	–	2	–	2	0	1
0,1,2:0,1,2	4	4	4	4	4	4	4	4	4	3	3	3	3	3	3	3	3	3	3	3	3	3	3	3	3	3	3	2	2	2	2	2	2	2	2	2	2	2	2	2	2	2	1	1	1	1	1	1	0
