# Supplementary material for: The Polytope Formalism: application to molecular constitution and the prospect of a complete description of Chemical Space
Source: Chem Sci. 2026 Jan 8;17(4):2102–18. doi: 10.1039/d5sc08813e (PMC12780917; doi:10.1039/d5sc08813e)
Supplement: SC-017-D5SC08813E-s001 [file SC-017-D5SC08813E-s001.zip › publication files/motions order outputs/S4B1 motions order table.pdf]

	0	1	2	3	0,1	1,2	2,3	0,3	0,2	1,3	1,2	0,2	0,1	0,1,2	0,1,2
0	0	–	–	–	1	–	–	1	1	–	–	1	1	2	2
1	–	0	–	–	1	1	–	–	–	1	1	–	1	2	2
2	–	–	0	–	–	1	1	–	1	–	1	1	–	2	2
3	–	–	–	0	–	–	1	1	–	1	–	–	–	–	–
0,1	1	1	–	–	0	–	–	–	–	–	–	–	0	1	1
1,2	–	1	1	–	–	0	–	–	–	–	0	–	–	1	1
2,3	–	–	1	1	–	–	0	–	–	–	–	–	–	–	–
0,3	1	–	–	1	–	–	–	0	–	–	–	–	–	–	–
0,2	1	–	1	–	–	–	–	–	0	–	–	0	–	1	1
1,3	–	1	–	1	–	–	–	–	–	0	–	–	–	–	–
1,2	–	1	1	–	–	0	–	–	–	–	0	–	–	1	1
0,2	1	–	1	–	–	–	–	–	0	–	–	0	–	1	1
0,1	1	1	–	–	0	–	–	–	–	–	–	–	0	1	1
0,1,2	2	2	2	–	1	1	–	–	1	–	1	1	1	0	0
0,1,2	2	2	2	–	1	1	–	–	1	–	1	1	1	0	0
