# Supplementary figures and images for: The Polytope Formalism: application to molecular constitution and the prospect of a complete description of Chemical Space
Source: Chem Sci. 2026 Jan 8;17(4):2102–18. doi: 10.1039/d5sc08813e (PMC12780917; doi:10.1039/d5sc08813e)

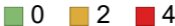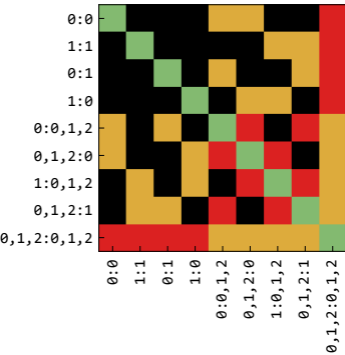

Supplement: SC-017-D5SC08813E-s001 [file SC-017-D5SC08813E-s001.zip › publication files/motions order outputs/S2B2 motions order plot.pdf]

0 2 4 6

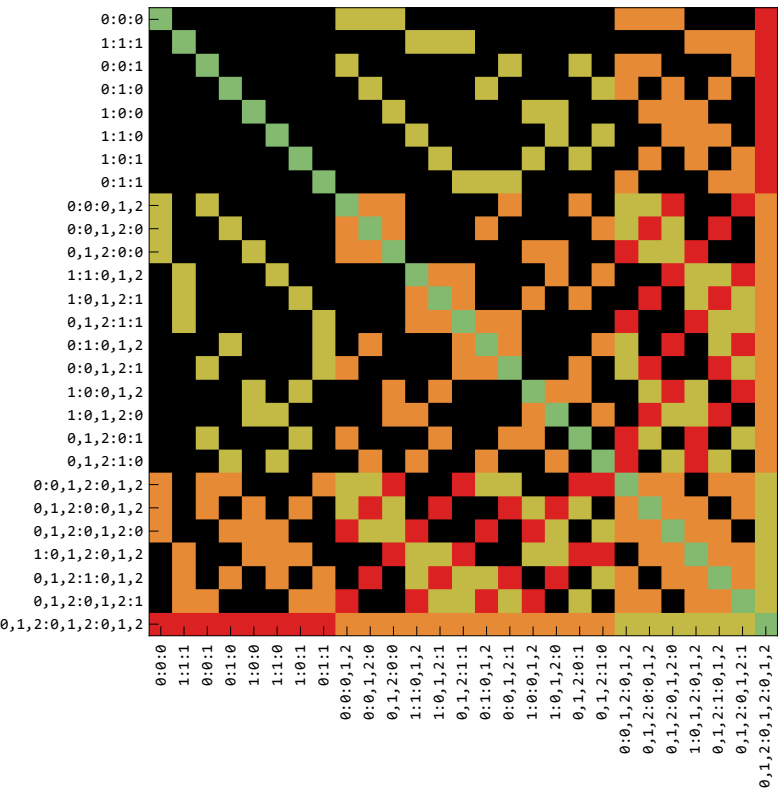

Supplement: SC-017-D5SC08813E-s001 [file SC-017-D5SC08813E-s001.zip › publication files/motions order outputs/S2B3 motions order plot.pdf]

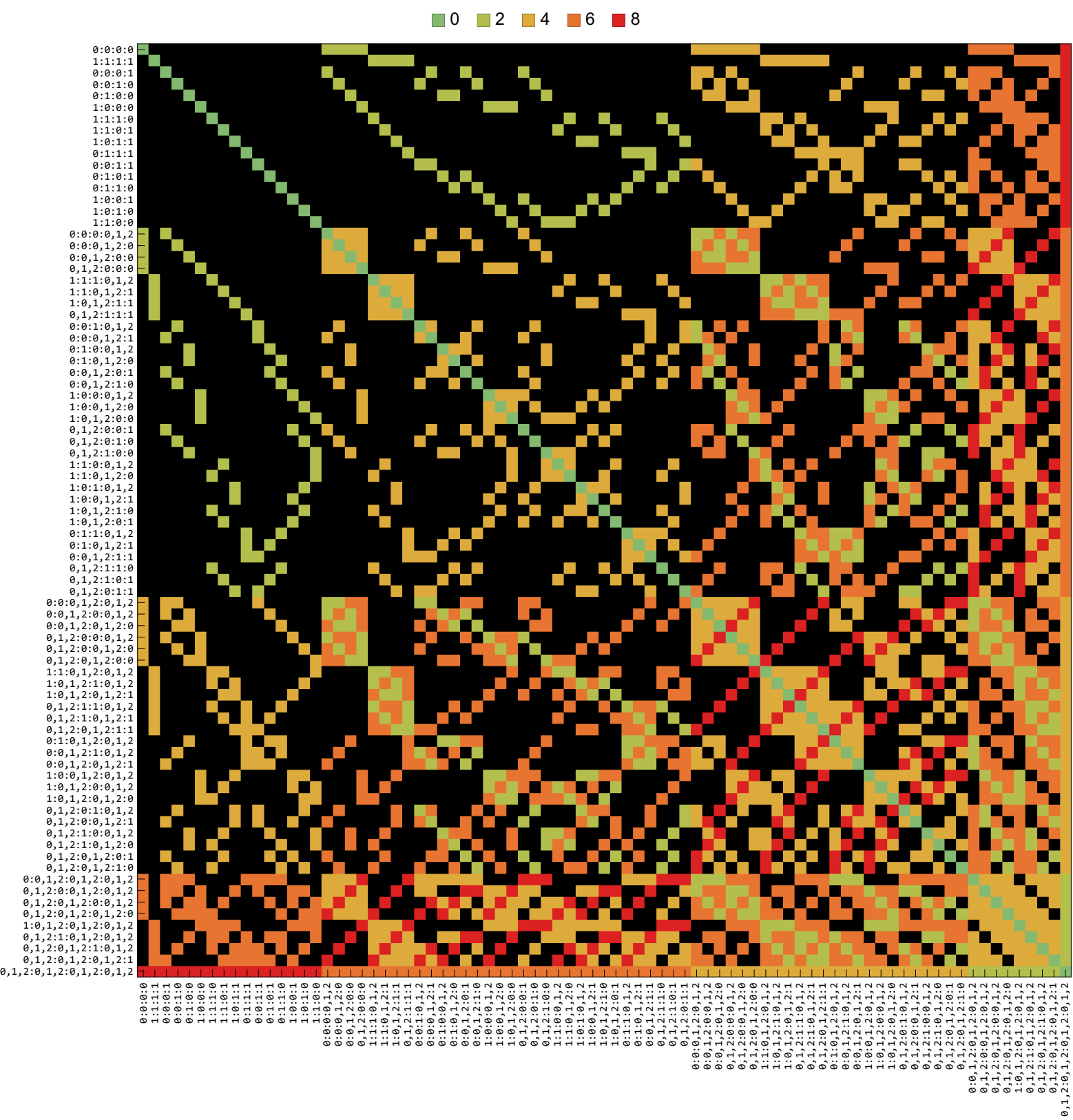

Supplement: SC-017-D5SC08813E-s001 [file SC-017-D5SC08813E-s001.zip › publication files/motions order outputs/S2B4 motions order plot.pdf]

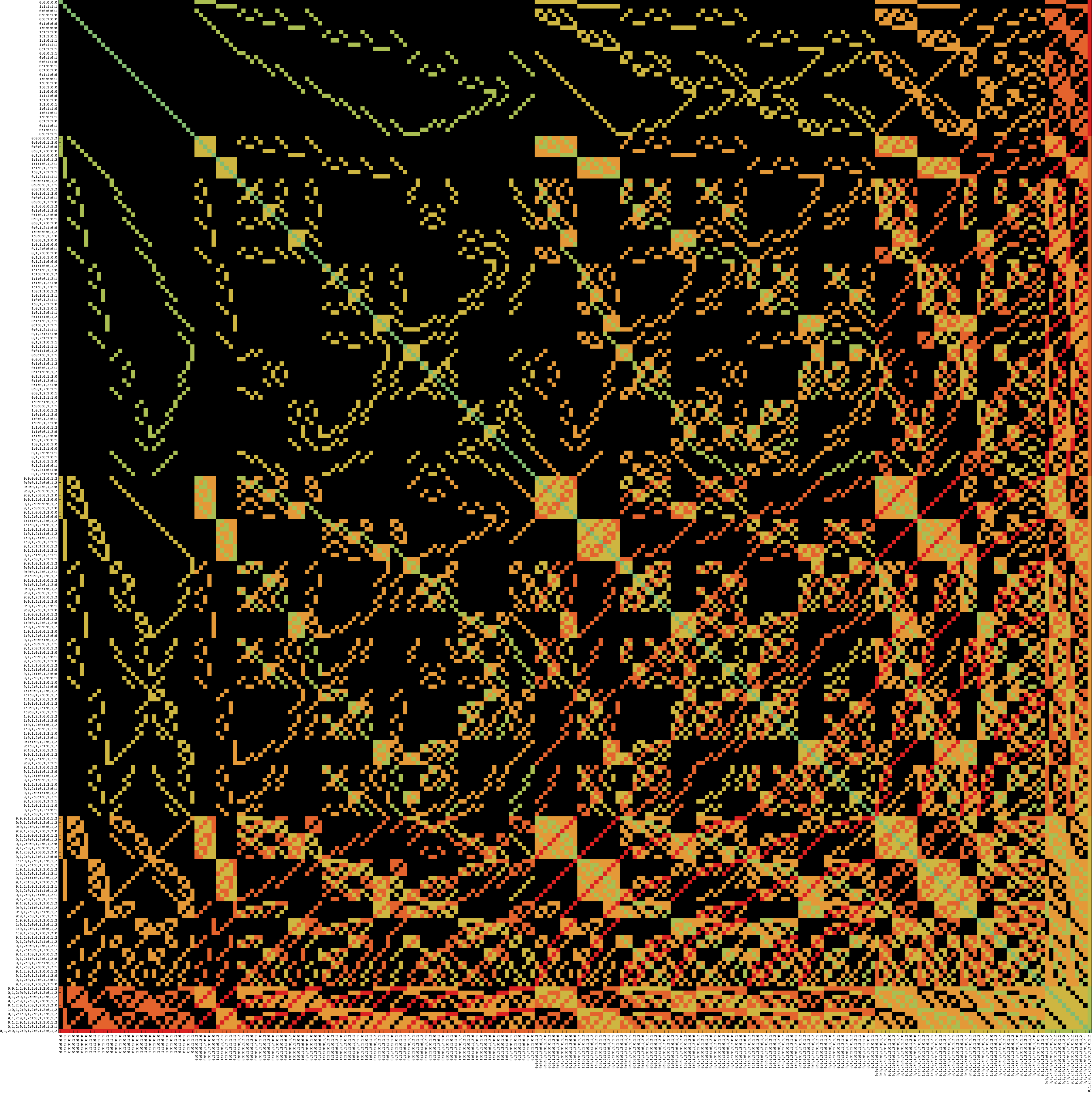

Supplement: SC-017-D5SC08813E-s001 [file SC-017-D5SC08813E-s001.zip › publication files/motions order outputs/S2B5 motions order plot.pdf]

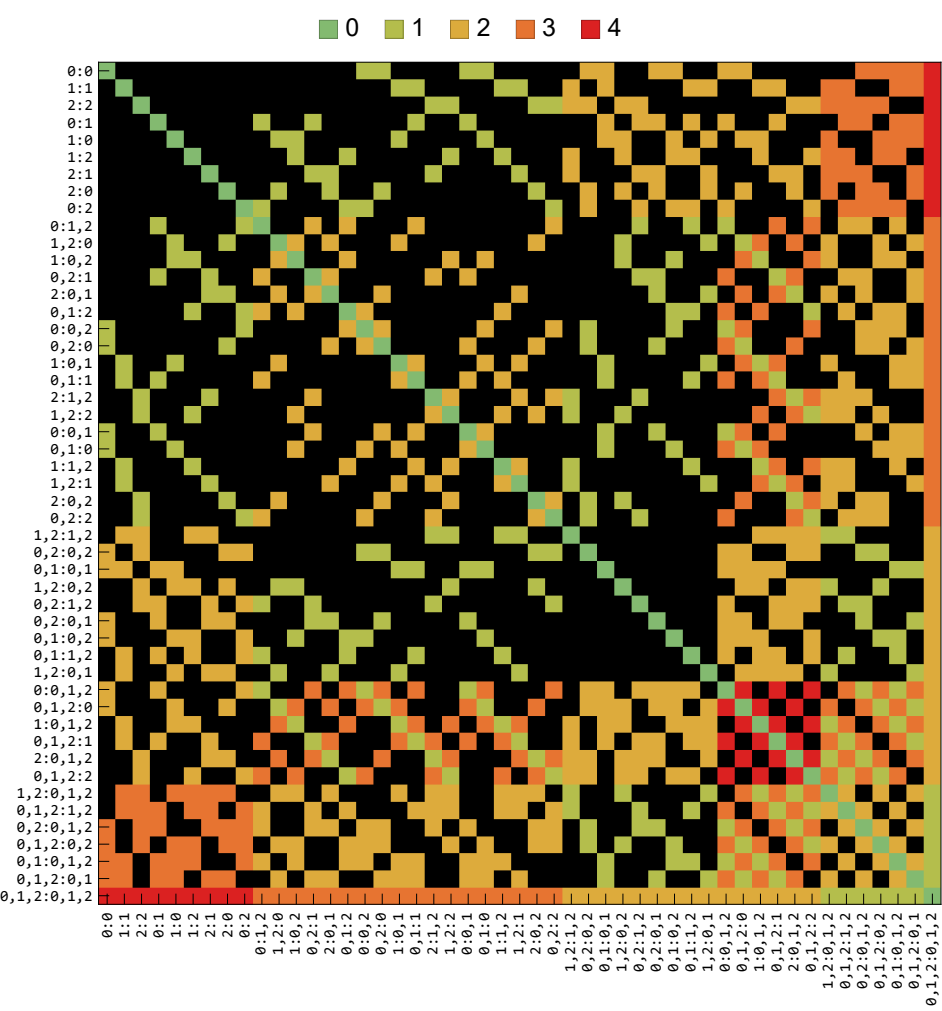

Supplement: SC-017-D5SC08813E-s001 [file SC-017-D5SC08813E-s001.zip › publication files/motions order outputs/S3B2 motions order plot.pdf]

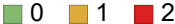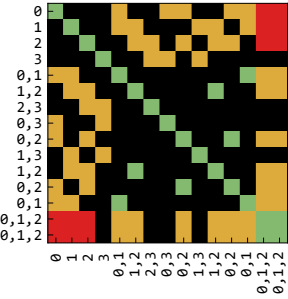

Supplement: SC-017-D5SC08813E-s001 [file SC-017-D5SC08813E-s001.zip › publication files/motions order outputs/S4B1 motions order plot.pdf]

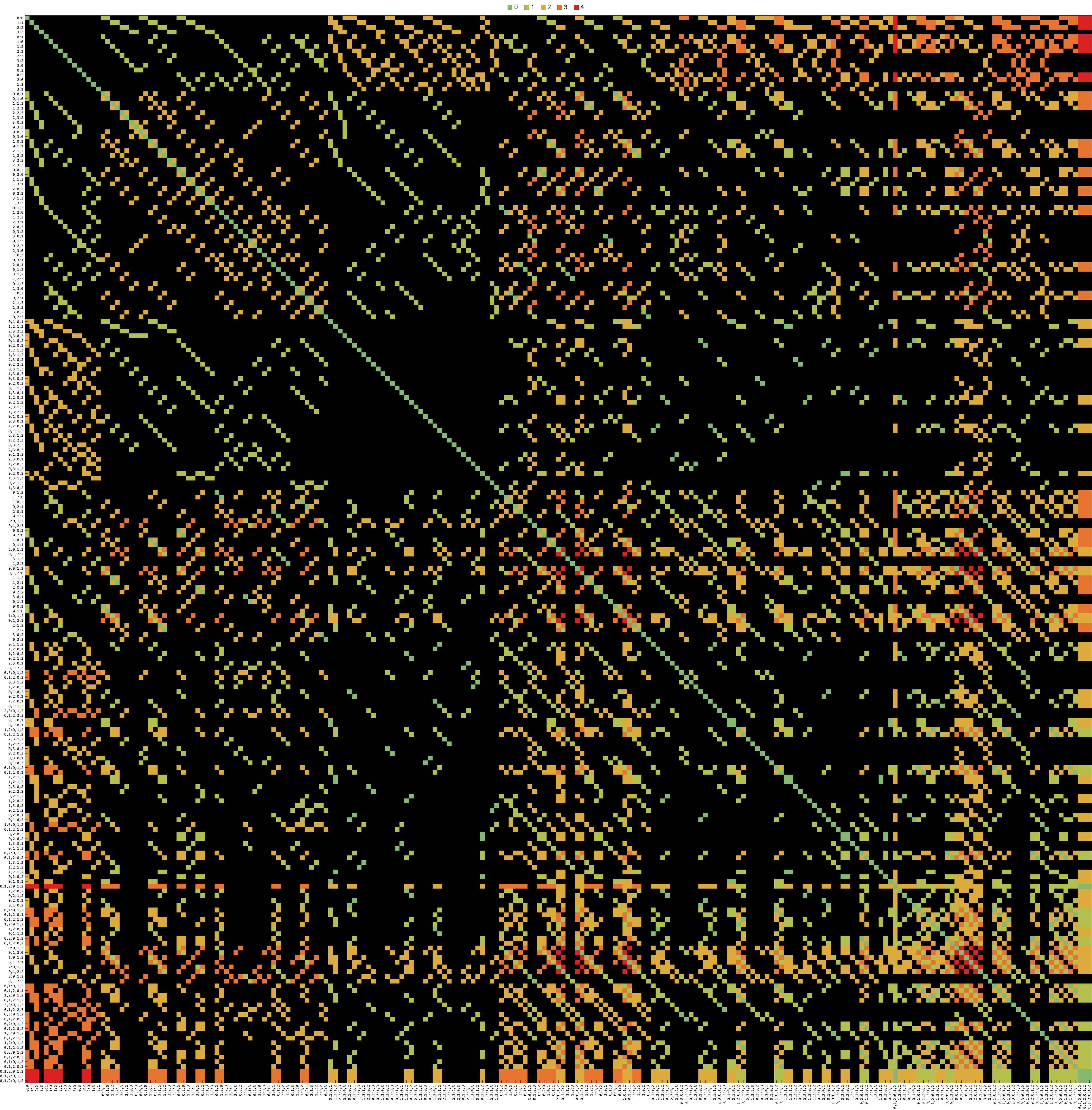

Supplement: SC-017-D5SC08813E-s001 [file SC-017-D5SC08813E-s001.zip › publication files/motions order outputs/S4B2 motions order plot.pdf]

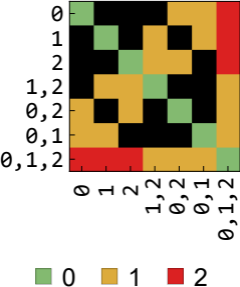

Supplement: SC-017-D5SC08813E-s001 [file SC-017-D5SC08813E-s001.zip › publication files/species/S3B1 motions order plot.pdf]

0 1 2 3 4

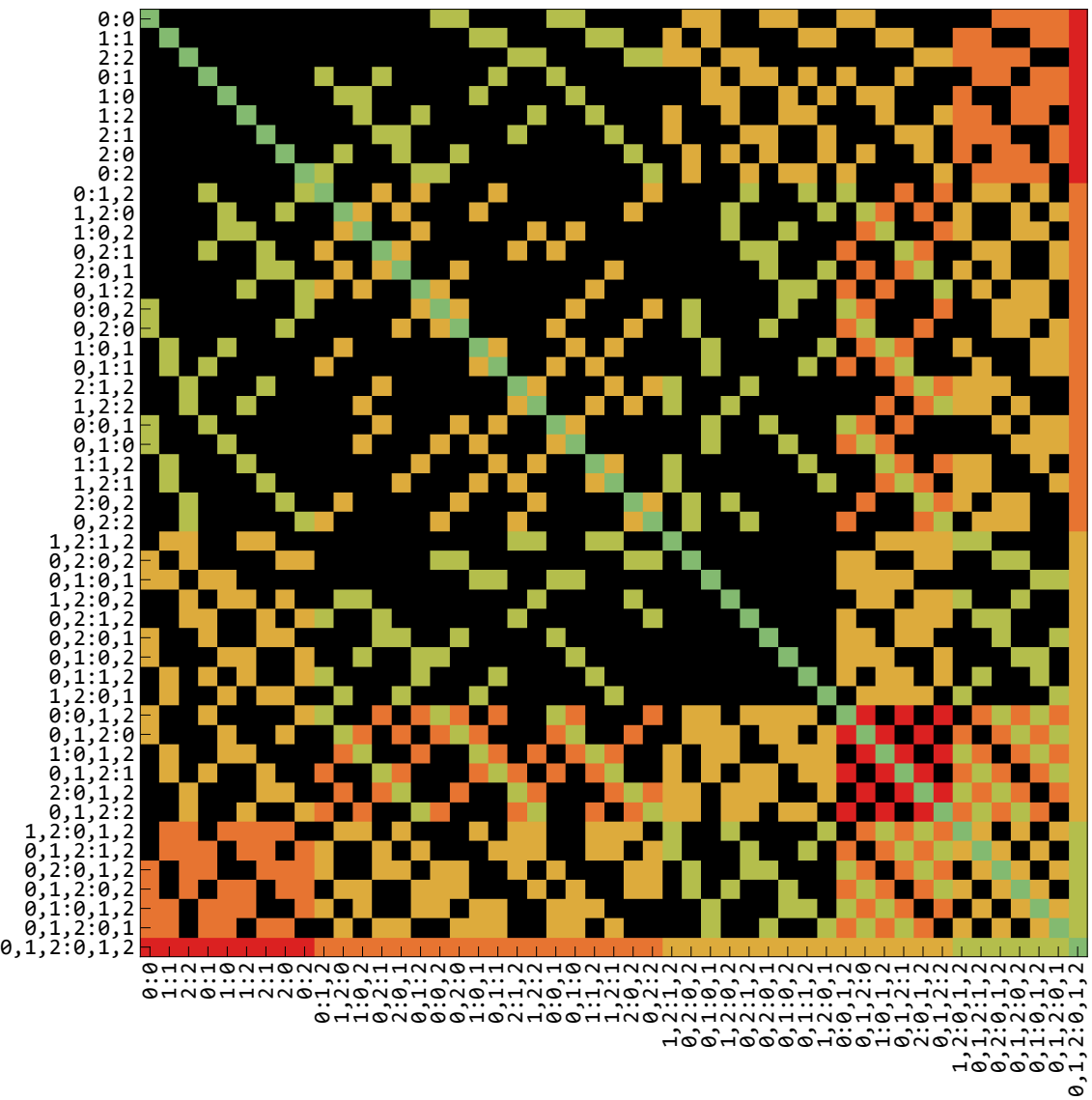

Supplement: SC-017-D5SC08813E-s001 [file SC-017-D5SC08813E-s001.zip › publication files/species/S3B2 motions order plot.pdf]
