# Supplementary material for: The Polytope Formalism: application to molecular constitution and the prospect of a complete description of Chemical Space
Source: Chem Sci. 2026 Jan 8;17(4):2102–18. doi: 10.1039/d5sc08813e (PMC12780917; doi:10.1039/d5sc08813e)
Supplement: SC-017-D5SC08813E-s002 [file SC-017-D5SC08813E-s002.pdf]

Supplementary Information (SI) for Chemical Science.
This journal is © The Royal Society of Chemistry 2025

The Polytope Formalism: Application to Molecular Constitution and the Prospect of a Complete Description of Chemical Space

Supplementary Information

Peter J. Canfield[a*] and Maxwell J. Crossley[a*]

[a] School of Chemistry, The University of Sydney, NSW 2006, Australia
*peter.canfield@sydney.edu.au, maxwell.crossley@sydney.edu.au

SI1. Electronic files included in the Supplementary Information: 2
SI2. Reactions graphs historically used to work out the structure of benzene from its chloro-substituted isomers.... 3
SI3. Glossary – definitions within the Polytope Formalism 4
SI4. Why space groups are not used to define connectivity symmetry. 5
SI5. Formal mathematical definitions of taxa in the unconstrained *n*-atom class 6
SI6. Bond order 8
SI7. Resolving 3-centre and polyhapto bonding schemes into atom-connection pairs 8
SI8. Desmotropic processes 8
SI9. Polytope Formalism of molecular constitution, unconstrained *n*-atom class taxonomy 9
SI10. Fundamental Steric Constraint for otherwise unconstrained *N*-atom-connectivity species.... 18
SI11. Compact descriptive symbols for naming species and relationship with chemical nomenclature 19
SI12. Modular Structure Theorem 20
SI13. Enumeration of families and species under the for $\mathcal{S}_m\mathcal{B}_n$ partitioned approach 21
SI14. Software to generate families, genera, and species for $\mathcal{S}_m\mathcal{B}_n$ with m, $n \leq 8$ 21
SI15. Formal definition for motions-order relationships between configurations 24
SI16. Species configurations – PES map for free-base subporphyrin partitioned as $\mathcal{S}_3\mathcal{B}_2$ using genera 11 – 16 26
SI17. All genera for tautomerism in two $\mathcal{S}_3\mathcal{B}_2$ systems.... 27
SI18. Genera for $\mathcal{S}_2\mathcal{B}_2$ partitioned [dienophile + diene] system allowing for dissociation 28
SI19. Optimised Cartesian coordinates with key calculation details, results, and systematic chemical names.... 30

SI1. Electronic files included in the Supplementary Information:

The Fortran code "genus_direct.for" and PC executable "genus_direct.exe" (written by Prof. Jeffrey R. Reimers, ORCID: 0000-0001-5157-7422) generates data files, many of which are also provided in the accompanying folder "species".

The Mathematical script "N-atom class graphs.nb" generates the outputs given in Polytope Formalism of molecular constitution, unconstrained n-atom class taxonomy.

The Mathematical script "SmBn partitioned Polytope Formalism of molecular constitution graphing.nb" allows for customised reaction graphs to be constructed using the data in the "species" folder. The folder "graphing outputs" containing select outputs from this software.

The Mathematical script "free-base subporphyrin PES.nb" depicts the potential energy surface (Fig. 13 in the main text) and its toroidal mapping, given definable and arbitrary angular phase shifts.

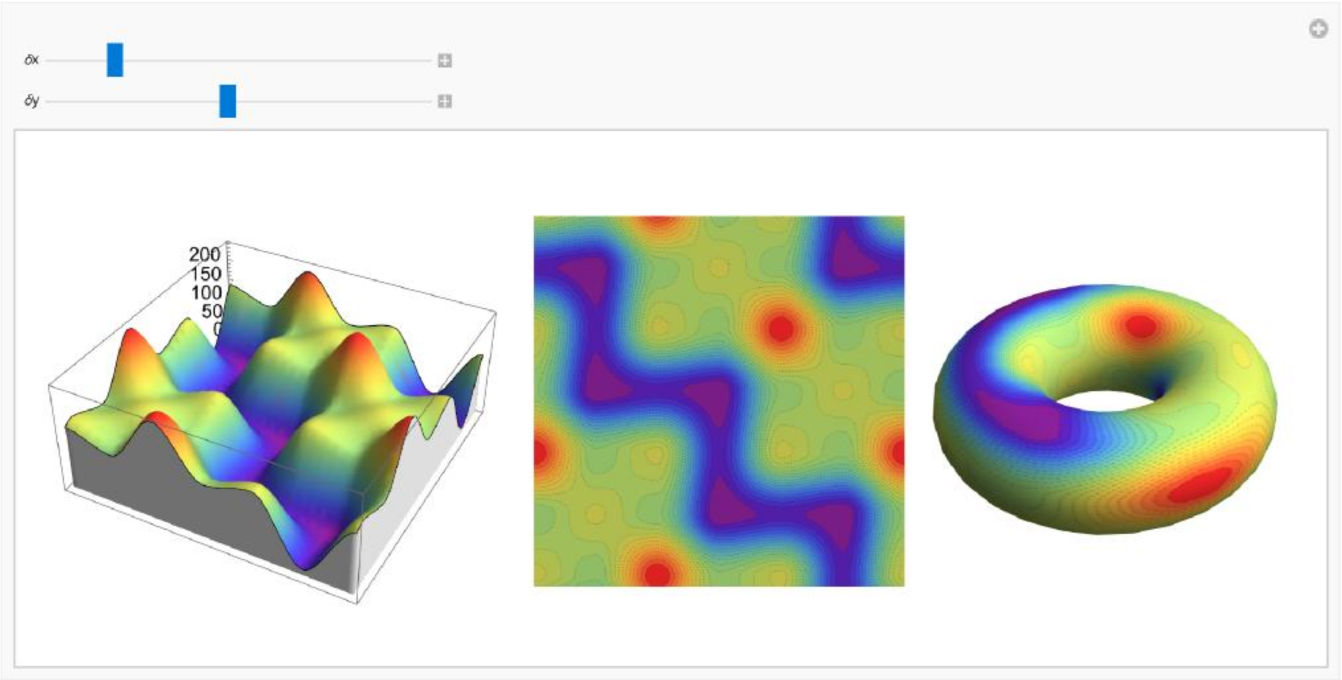

The Mathematical script "motions order matrix.nb" calculates the motions-order matrices given in Species configurations – PES map for free-base subporphyrin partitioned as $\boldsymbol{S_3\mathcal{B}2}$ using genera $11-16$.

Folder: "motions order outputs" containing select outputs from this software

SI2. Reactions graphs historically used to work out the structure of benzene from its chloro-substituted isomers

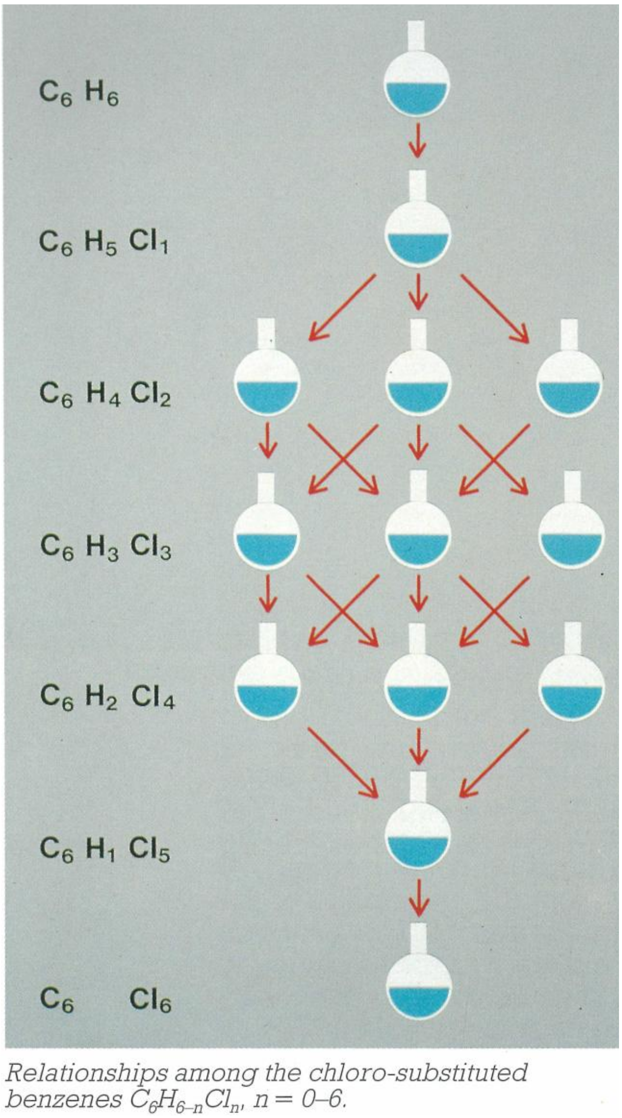

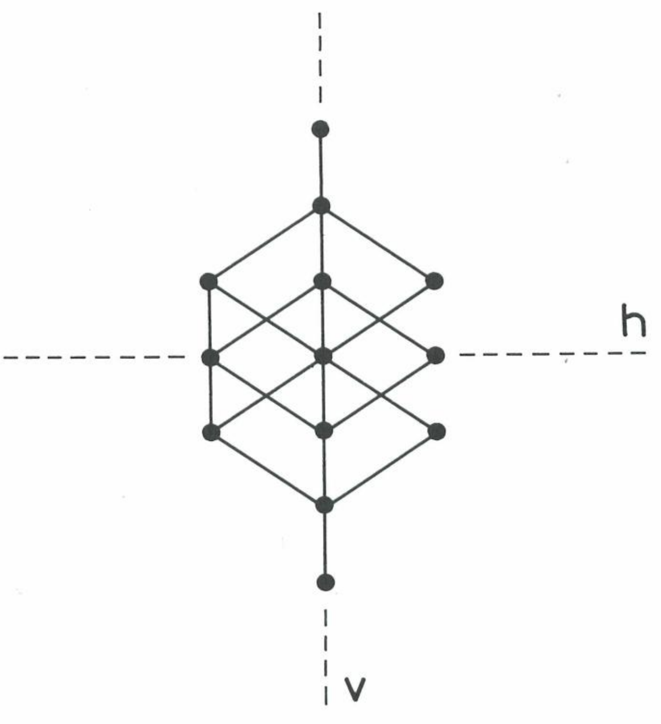

Figure SI.1 Figures from pages 46 and 47 in “Reflections on Symmetry in Chemistry… and Elsewhere”, Heilbronner, E., Dunitz, J. D., Basel: VCHA; Veinheim; Basel; Cambridge; New York: VCH, 1993. Reproduced with permission under CC-BY-NC-ND.

SI3. Glossary – definitions within the Polytope Formalism

**Stereotropic**	**Adjective**; from Ancient Greek ***στερεός*** *stereós* "solid" (in the *spatial* sense by analogy with stereoscopic) + ***τρόπος*** *trópos* "a turn, a change"; of or relating to a change in spatial arrangements of atoms in a molecular entity without changing the atom connectivity.
**Desmotropic**	**Adjective**; from Ancient Greek ***δεσμός*** *desmós* "bond, connection" + ***τρόπος*** *trópos* "a turn, a change"; of or relating to a change in atom connectivity in a molecular entity.
**Nucleotropic**	**Adjective**; from Latin *nŭ̆cleus* "pertaining to a small nut" (through English *nucleus* "pertaining to the nucleus of an atom) + ***τρόπος*** *trópos* "a turn, a change"; of or relating to a change in the nuclear details (isotopes, nuclear state, molecular nuclear state) in a molecular entity.
**Electropic**	**Adjective**; from Ancient Greek ***ἤλεκτρον*** *ḗlektron* "amber" (through English *electronic* "pertaining to electrons) + ***τρόπος*** *trópos* "a turn, a change"; of or relating to a change in the electronic configuration of a molecular entity.
**Rovitropic**	**Adjective**; from Latin ***rotatiō*** "to turn" + Latin ***vibrātiō*** "a shaking" (through English *rovibronic* "pertaining to the rotational and vibrational states of an electronic state) + ***τρόπος*** *trópos* "a turn, a change"; of or relating to a change in the vibrational and/or rotational state of a molecular entity.

$\mathbf{R^{c}_{st}1}$	**Process symbol:** rearrangement, concerted, stereotropic, unimolecular.
$\mathbf{R^{c}_{de}}$	**Process symbol:** rearrangement, concerted, desmotropic.
$\mathbf{R^{c}_{de}1}$, $\mathbf{R^{c}_{de}2}$, $\mathbf{R^{c}_{de}}\boldsymbol{k}$	**Process symbol:** rearrangement, concerted, desmotropic, unimolecular; bimolecular; *k*-molecular.
$\mathbf{R^{c}_{ns}}$	**Process symbol:** rearrangement, concerted, nucleotropic.
$\mathbf{R^{c}_{el}}$	**Process symbol:** rearrangement, concerted, electrotropic.
$\mathbf{R^{c}_{rv}}$	**Process symbol:** rearrangement, concerted, rovitropic.

**Class**	**Taxon:** high-level organisational taxon; adjectival form *classic*.<br>Stereoisomerism example: AB_n<br>Molecular constitution example: 5-atom;
**Family**	**Taxon:** second highest-level organisational taxon; adjectival form *familial.*<br>Stereoisomerism example: AB_4<br>Molecular constitution example: (5-atom | 2-connections)
**Genus**	**Taxon:** second lowest-level organisational taxon; adjectival form *generic*.<br>Stereoisomerism example: (AB_4 | *T*-4)<br>Molecular constitution example: (5-atom | 2-connections | 1-common-vertex)
**Species**	**Taxon:** lowest-level organisational taxon; adjectival form *specific*.<br>Stereoisomerism example: (AB_4 | *T*-4-*R*)<br>Molecular constitution example: (5-atom | 2-connections | 1-common-vertex | {{1,2},{2,3}})

SI4. Why space groups are not used to define connectivity symmetry.

Three-dimensional space groups (e.g., D_{3h}) are not used to define site symmetry in the $\mathcal{S}_m\mathcal{B}_n$ partitioning approach because the Polytope Formalism application to molecular constitution operates on an *abstract connectivity space*, not on a full spatial embedding.

In this framework, "symmetry" refers to *combinatorial equivalence* among bonding sites, not to physical operations in 3D Euclidean space. The $\mathcal{S}_m\mathcal{B}_n$ model enumerates and relates all possible connectivities between m sites and n bonders, independent of any particular geometry. The relevant symmetry groups are therefore discrete permutation groups (e.g., $\mathbb{Z}_3$, D_2) that act on the indices of sites and bonders, describing topological equivalence under relabelling rather than geometric rotation, reflection, or inversion.

By contrast, a space group such as D_{3h} encodes continuous geometric symmetry operation**s** (rotations, reflections, inversions) in three-dimensional coordinate space and depends on a chosen molecular embedding. Because the $\mathcal{S}_m\mathcal{B}_n$ partitioning formalism aims to define a *universal*, geometry-independent representation of chemical connectivity, these spatial details are deliberately abstracted away.

Thus:

- The **site symmetry** in the $\mathcal{S}_m\mathcal{B}_n$ sense ($\mathbb{Z}_3$, D_2, etc.) represents *equivalence of connection roles* within the abstract polytope.
- The **point or space group** (e.g., D_{3h}) may describe one possible realisation of that polytope in Euclidean space, but it is *not intrinsic* to the connectivity definition.
- Using space groups would tie the formalism to specific molecular geometries, breaking the intended separation between *molecular constitution* (connectivity topology) and its possible geometric realisation(s).

Hence, site symmetry in the $\mathcal{S}_m\mathcal{B}_n$ framework is defined purely *topologically and combinatorially*, not crystallographically or spatially, ensuring that the analysis generalises across all geometries consistent with the same connectivity graph.

Whilst this explanation is presented for the $\mathcal{S}_m\mathcal{B}_n$-partitioning approach, the general principle applies to *any* implementation of the Polytope Formalism applied to molecular constitution.

We use the $\mathbb{Z}_n$ convention for describing the n-cyclic group in place of C_n to avoid confusion with the 3D space group C_n.

SI5. Formal mathematical definitions of taxa in the unconstrained n-atom class

Let $n$denote the number of atoms (vertices). We work exclusively with *simple graphs* (at most one edge between any pair of atoms and no self-loops). Let g_n be the number of labelled simple molecular graphs on n atoms. Since each of the $\binom{n}{2}$ possible edges may be present or absent, a simple count gives

$$g_n = 2^{\binom{n}{2}}.$$

Exponential generating function for all species (all graphs)

Define the exponential generating function (EGF)

$$G(x) = \sum_{n\geq 0} \frac{g_n}{n!} x^n = \sum_{n\geq 0} \frac{2^{\binom{n}{2}}}{n!} x^n \qquad \text{Eq. SI5.1}$$

Species in the unconstrained n-atom class correspond to labelled graphs and therefore to the coefficients of $G(x)$.

Connected species (connected molecular graphs)

Let c_n be the number of labelled *connected* molecular graphs on n atoms. It is a classical result of labelled combinatorics that the EGF for connected structures is the *formal logarithm* of the EGF for all structures:

$$C(x) = \log G(x) = \sum_{n\geq 1} \frac{c_n}{n!} x^n \qquad \text{Eq. SI5.2}$$

Here, log is interpreted as the *formal logarithm* in the ring of formal power series $\mathbb{Z}[\![x]\!]$, not as a real-valued analytic function. Because $G(x)$has integer coefficients and unit constant term, the formal identity

$$\log(1 + A(x)) = A(x) - \frac{A(x)^2}{2} + \frac{A(x)^3}{3} - \cdots \qquad \text{Eq. SI5.3}$$

holds for $A(x) \in x\mathbb{Z}[\![x]\!]$. Since formal power-series algebra preserves integer coefficients, this guarantees that each coefficient c_n is an integer and therefore counts a finite number of connected species.

Families (by edge count)

Let $f_{n,k}$denote the number of labelled simple graphs on $n$atoms with exactly k edges. Since choosing k edges from $\binom{n}{2}$ possibilities fully determines the graph,

$$f_{n,k} = \binom{\binom{n}{2}}{k} \qquad \text{Eq. SI5.4}$$

For fixed n, each choice of k gives a *family* in the taxonomic hierarchy. Summing over all k recovers Eq. (SI5.1).

Genera (isomorphism classes)

Two labelled graphs on n atoms belong to the same *genus* if they are isomorphic (i.e., identical up to relabelling of atoms). Let $u_{n,k}$ be the number of unlabelled (isomorphism-distinct) graphs on n atoms with k edges. A standard application of the Burnside lemma gives

$$u_{n,k} = \frac{1}{n!} \sum_{\sigma\in S_n} [y^k] \prod_{\text{cycles of } \sigma} (1 + y^\ell) \qquad \text{Eq. SI5.5}$$

where the product runs over the cycles of the permutation σ, ℓ is the length of each cycle, and $[y^k]F(y)$ denotes *coefficient extraction*: the coefficient of y^k in the formal power series $F(y)$.

The total number of genera for the n-atom class is therefore

$$U_n = \sum_{k=0}^{\binom{n}{2}} u_{n,k} \qquad \text{Eq. SI5.6}$$

These unlabelled genera correspond to the abstract polytopes in the Polytope Formalism and are the fundamental "shapes" of atom-connectivity configurations.

Species

A *species* corresponds to a specific labelled atom-connectivity configuration (a specific adjacency matrix). Thus, for the unconstrained n-atom class:

- **species**: $g_n = 2^{\binom{n}{2}}$
- **families**: indexed by edge count $k = 0,1,\dots,\binom{n}{2}$
- **genera**: unlabelled graphs $u_{n,k}$ within each family

This completes the formal mathematical specification of taxa for the unconstrained n-atom class used in the Polytope Formalism.

References

Harary, F. *Graph Theory*. Addison–Wesley, 1969.

Pólya, G.; Read, R. C. *Combinatorial Enumeration of Groups, Graphs, and Chemical Compounds*. Springer, 1987.

SI6. Bond order

While our framework deliberately abandons the notion of *integer bond order* (BO) in favour of a purely connectivity-based representation, it is instructive to clarify this rationale. Bond order, though widely used, is ultimately a *construct* rather than a physical observable. Methods for estimating BO span a spectrum – from simple empirical approaches based on internuclear distances (as implemented in many molecular visualisation tools), to quantum-mechanical treatments that compute overlap populations.

The empirical approach maps bond lengths to integer or half-integer BO values, whereas quantum methods yield continuous BO values that more accurately reflect chemical reality. This continuum underscores the artificiality of the discrete bond orders traditionally employed in chemical structure representation. In contrast, atom connectivity provides an unambiguous, element-agnostic foundation upon which topological and energetic analyses can be systematically built.

SI7. Resolving 3-centre and polyhapto bonding schemes into atom-connection pairs

The Polytope Formalism of consitutional isomerism relies strictly on atom connectivity between pairs of atoms. Multicentre and polyhapto bonding schemes must be resolved into connected pairs as shown in Figure 1.

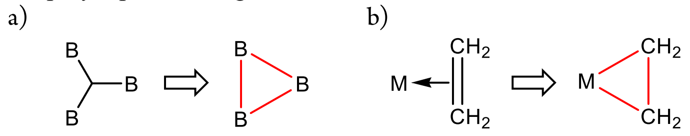

Figure SI.2 Resolving multicenter bonds as connected atom pairs. a) Resolving three-center bonds. d) Resolving an η^2 “side-on” bond.

SI8. Desmotropic processes

For a collection of atoms, a real or conceptual process that changes one atom-connectivity configuration into another, we describe as *desmotropic* [Ancient Greek δεσκός (desmós), “bond, connection” + τροπικός (tropikos), “of or pertaining to a change]. Desmotropic is thus the general term that encompasses *all* changes in connectivity and thus all bond breaking/making processes. It follows that the established concepts of pericyclic and sigmatropic processes are subsets of desmotropic processes.

A key concept within the formalism, when discussing physical mechanisms, is that of a concerted unimolecular desmotropic rearrangement for which we introduce the symbol $R^{c}_{de}1$ to the literature. $R^{c}_{de}1$ processes are the molecular constitution equivalent of concerted unimolecular *stereotropic* rearrangement ($R^{c}_{st}1$) processes as we introduced for stereoisomerism. If dissociation/association into/from several fragments is permitted then the bimolecular, termolecular, …, *k*-molecular processes can similarly be described as $R^{c}_{de}2$, $R^{c}_{de}3$, …$R^{c}_{de}k$, respectively. Finally, these may be described using a general term R^{c}_{de} without explicit reference to the molecularity number.

SI9. Polytope Formalism of molecular constitution, unconstrained *n*-atom class taxonomy

The following figures present the taxonomic organisation of the unconstrained n-atom classes for $n = 1$ to 5. The initial set lists the genera (no graph vertex indices assigned). Immediately below this are the cardinalities of the lowest subsets. Lastly is an exhaustive listing of all species grouped into their respective families and genera.

Molecular constitution 1-atom Class

Graph isomorphisms (Genera):

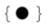

Species counts:

{{1}}

Taxonomically grouped species:

F_1G_1

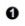

$\begin{pmatrix} 0 \end{pmatrix}$

Molecular constitution 2-atom Class

Graph isomorphisms (Genera):

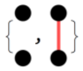

Species counts:

{{1}, {1}}

Taxonomically grouped species:

F_1G_1

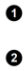

$\begin{pmatrix} 0 & 0 \\ 0 & 0 \end{pmatrix}$

F_2G_1

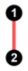

$\begin{pmatrix} 0 & 1 \\ 1 & 0 \end{pmatrix}$

Molecular constitution 3-atom Class

Graph isomorphisms (Genera):

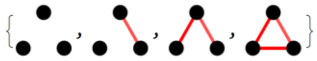

Species counts:

{{1}, {3}, {3}, {1}}

Taxonomically grouped species:

F_1G_1

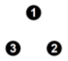

$\begin{pmatrix} 0 & 0 & 0 \\ 0 & 0 & 0 \\ 0 & 0 & 0 \end{pmatrix}$

F_2G_1

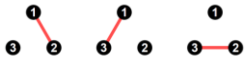

$\begin{pmatrix} 0 & 1 & 0 \\ 1 & 0 & 0 \\ 0 & 0 & 0 \end{pmatrix}$ $\begin{pmatrix} 0 & 0 & 1 \\ 0 & 0 & 0 \\ 1 & 0 & 0 \end{pmatrix}$ $\begin{pmatrix} 0 & 0 & 0 \\ 0 & 0 & 1 \\ 0 & 1 & 0 \end{pmatrix}$

F_3G_1

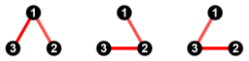

$\begin{pmatrix} 0 & 1 & 1 \\ 1 & 0 & 0 \\ 1 & 0 & 0 \end{pmatrix}$ $\begin{pmatrix} 0 & 1 & 0 \\ 1 & 0 & 1 \\ 0 & 1 & 0 \end{pmatrix}$ $\begin{pmatrix} 0 & 0 & 1 \\ 0 & 0 & 1 \\ 1 & 1 & 0 \end{pmatrix}$

F_4G_1

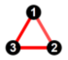

$\begin{pmatrix} 0 & 1 & 1 \\ 1 & 0 & 1 \\ 1 & 1 & 0 \end{pmatrix}$

Molecular constitution 4-atom Class

Graph isomorphisms (Genera):

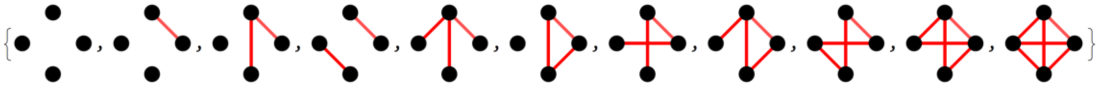

Species counts:

{{1}, {6}, {12, 3}, {4, 4, 12}, {12, 3}, {6}, {1}}

Taxonomically grouped species:

F_1G_1

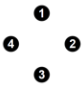

$\begin{pmatrix}0&0&0&0\\0&0&0&0\\0&0&0&0\\0&0&0&0\end{pmatrix}$

F_2G_1

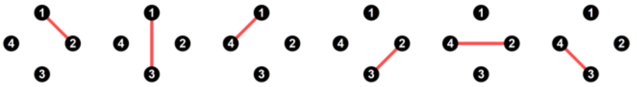

$\begin{pmatrix}0&1&0&0\\1&0&0&0\\0&0&0&0\\0&0&0&0\end{pmatrix}$ $\begin{pmatrix}0&0&1&0\\0&0&0&0\\1&0&0&0\\0&0&0&0\end{pmatrix}$ $\begin{pmatrix}0&0&0&1\\0&0&0&0\\0&0&0&0\\1&0&0&0\end{pmatrix}$ $\begin{pmatrix}0&0&0&0\\0&0&1&0\\0&1&0&0\\0&0&0&0\end{pmatrix}$ $\begin{pmatrix}0&0&0&0\\0&0&0&1\\0&0&0&0\\0&1&0&0\end{pmatrix}$ $\begin{pmatrix}0&0&0&0\\0&0&0&0\\0&0&0&1\\0&0&1&0\end{pmatrix}$

F_3G_1

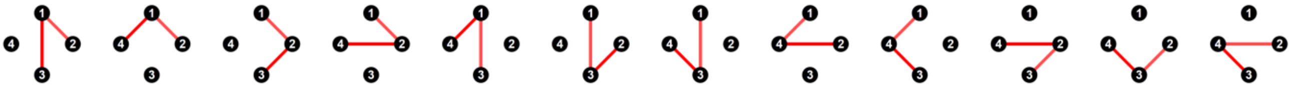

$\begin{pmatrix}0&1&1&0\\1&0&0&0\\1&0&0&0\\0&0&0&0\end{pmatrix}$ $\begin{pmatrix}0&1&0&1\\1&0&0&0\\0&0&0&0\\1&0&0&0\end{pmatrix}$ $\begin{pmatrix}0&1&0&0\\1&0&1&0\\0&1&0&0\\0&0&0&0\end{pmatrix}$ $\begin{pmatrix}0&1&0&0\\1&0&0&1\\0&0&0&0\\0&1&0&0\end{pmatrix}$ $\begin{pmatrix}0&0&1&1\\0&0&0&0\\1&0&0&0\\1&0&0&0\end{pmatrix}$ $\begin{pmatrix}0&0&1&0\\0&0&1&0\\1&1&0&0\\0&0&0&0\end{pmatrix}$ $\begin{pmatrix}0&0&1&0\\0&0&0&0\\1&0&0&1\\0&0&1&0\end{pmatrix}$ $\begin{pmatrix}0&0&0&1\\0&0&0&1\\0&0&0&0\\1&1&0&0\end{pmatrix}$ $\begin{pmatrix}0&0&0&1\\0&0&0&0\\0&0&0&1\\1&0&1&0\end{pmatrix}$ $\begin{pmatrix}0&0&0&0\\0&0&1&1\\0&1&0&0\\0&1&0&0\end{pmatrix}$ $\begin{pmatrix}0&0&0&0\\0&0&1&0\\0&1&0&1\\0&0&1&0\end{pmatrix}$ $\begin{pmatrix}0&0&0&0\\0&0&0&1\\0&0&0&1\\0&1&1&0\end{pmatrix}$

F_3G_2

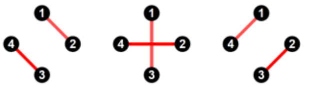

$\begin{pmatrix}0&1&0&0\\1&0&0&0\\0&0&0&1\\0&0&1&0\end{pmatrix}$ $\begin{pmatrix}0&0&1&0\\0&0&0&1\\1&0&0&0\\0&1&0&0\end{pmatrix}$ $\begin{pmatrix}0&0&0&1\\0&0&1&0\\0&1&0&0\\1&0&0&0\end{pmatrix}$

F_4G_1

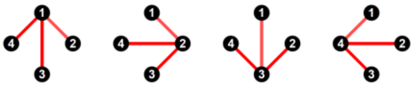

$\begin{pmatrix}0&1&1&1\\1&0&0&0\\1&0&0&0\\1&0&0&0\end{pmatrix}$ $\begin{pmatrix}0&1&0&0\\1&0&1&1\\0&1&0&0\\0&1&0&0\end{pmatrix}$ $\begin{pmatrix}0&0&1&0\\0&0&1&0\\1&1&0&1\\0&0&1&0\end{pmatrix}$ $\begin{pmatrix}0&0&0&1\\0&0&0&1\\0&0&0&1\\1&1&1&0\end{pmatrix}$

F_4G_2

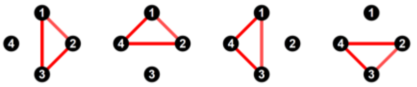

$\begin{pmatrix}0&1&1&0\\1&0&1&0\\1&1&0&0\\0&0&0&0\end{pmatrix}$ $\begin{pmatrix}0&1&0&1\\1&0&0&1\\0&0&0&0\\1&1&0&0\end{pmatrix}$ $\begin{pmatrix}0&0&1&1\\0&0&0&0\\1&0&0&1\\1&0&1&0\end{pmatrix}$ $\begin{pmatrix}0&0&0&0\\0&0&1&1\\0&1&0&1\\0&1&1&0\end{pmatrix}$

F_4G_3

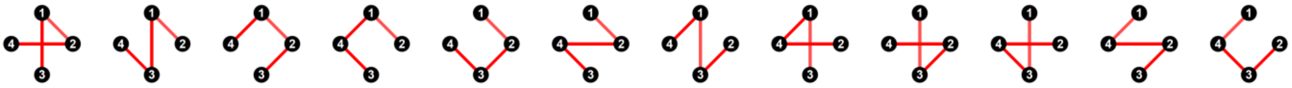

$\begin{pmatrix}0&1&1&0\\1&0&0&1\\1&0&0&0\\0&1&0&0\end{pmatrix}$ $\begin{pmatrix}0&1&1&0\\1&0&0&0\\1&0&0&1\\0&0&1&0\end{pmatrix}$ $\begin{pmatrix}0&1&0&1\\1&0&1&0\\0&1&0&0\\1&0&0&0\end{pmatrix}$ $\begin{pmatrix}0&1&0&1\\1&0&0&0\\0&0&0&1\\1&0&1&0\end{pmatrix}$ $\begin{pmatrix}0&1&0&0\\1&0&1&0\\0&1&0&1\\0&0&1&0\end{pmatrix}$ $\begin{pmatrix}0&1&0&0\\1&0&0&1\\0&0&0&1\\0&1&1&0\end{pmatrix}$ $\begin{pmatrix}0&0&1&1\\0&0&1&0\\1&1&0&0\\1&0&0&0\end{pmatrix}$ $\begin{pmatrix}0&0&1&1\\0&0&0&1\\1&0&0&0\\1&1&0&0\end{pmatrix}$ $\begin{pmatrix}0&0&1&0\\0&0&1&1\\1&1&0&0\\0&1&0&0\end{pmatrix}$ $\begin{pmatrix}0&0&1&0\\0&0&0&1\\1&0&0&1\\0&1&1&0\end{pmatrix}$ $\begin{pmatrix}0&0&0&1\\0&0&1&1\\0&1&0&0\\1&1&0&0\end{pmatrix}$ $\begin{pmatrix}0&0&0&1\\0&0&1&0\\0&1&0&1\\1&0&1&0\end{pmatrix}$

Molecular constitution 4-atom Class continued

F_5G_1

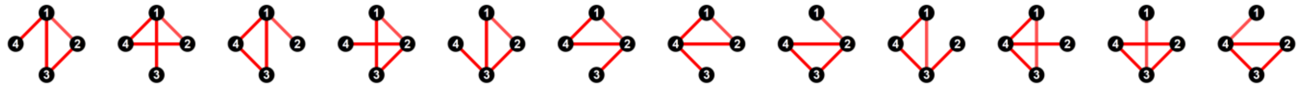

$$\begin{pmatrix}0&1&1&1\\1&0&1&0\\1&1&0&0\\1&0&0&0\end{pmatrix}\begin{pmatrix}0&1&1&1\\1&0&0&1\\1&0&0&0\\1&1&0&0\end{pmatrix}\begin{pmatrix}0&1&1&1\\1&0&0&0\\1&0&0&1\\1&0&1&0\end{pmatrix}\begin{pmatrix}0&1&1&0\\1&0&1&1\\1&1&0&0\\0&1&0&0\end{pmatrix}\begin{pmatrix}0&1&1&0\\1&0&1&0\\1&1&0&1\\0&0&1&0\end{pmatrix}\begin{pmatrix}0&1&0&1\\1&0&1&1\\0&1&0&0\\1&1&0&0\end{pmatrix}\begin{pmatrix}0&1&0&1\\1&0&0&1\\0&0&0&1\\1&1&1&0\end{pmatrix}\begin{pmatrix}0&1&0&0\\1&0&1&1\\0&1&0&1\\0&1&1&0\end{pmatrix}\begin{pmatrix}0&0&1&1\\0&0&1&0\\1&1&0&1\\1&0&1&0\end{pmatrix}\begin{pmatrix}0&0&1&1\\0&0&0&1\\1&0&0&1\\1&1&1&0\end{pmatrix}\begin{pmatrix}0&0&1&0\\0&0&1&1\\1&1&0&1\\0&1&1&0\end{pmatrix}\begin{pmatrix}0&0&0&1\\0&0&1&1\\0&1&0&1\\1&1&1&0\end{pmatrix}$$

F_5G_2

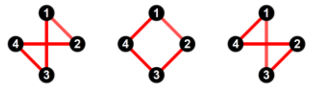

$$\begin{pmatrix}0&1&1&0\\1&0&0&1\\1&0&0&1\\0&1&1&0\end{pmatrix}\begin{pmatrix}0&1&0&1\\1&0&1&0\\0&1&0&1\\1&0&1&0\end{pmatrix}\begin{pmatrix}0&0&1&1\\0&0&1&1\\1&1&0&0\\1&1&0&0\end{pmatrix}$$

F_6G_1

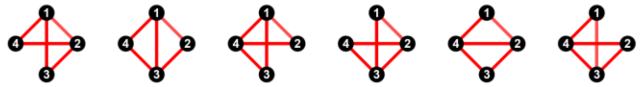

$$\begin{pmatrix}0&1&1&1\\1&0&1&1\\1&1&0&0\\1&1&0&0\end{pmatrix}\begin{pmatrix}0&1&1&1\\1&0&1&0\\1&1&0&1\\1&0&1&0\end{pmatrix}\begin{pmatrix}0&1&1&1\\1&0&0&1\\1&0&0&1\\1&1&1&0\end{pmatrix}\begin{pmatrix}0&1&1&0\\1&0&1&1\\1&1&0&1\\0&1&1&0\end{pmatrix}\begin{pmatrix}0&1&0&1\\1&0&1&1\\0&1&0&1\\1&1&1&0\end{pmatrix}\begin{pmatrix}0&0&1&1\\0&0&1&1\\1&1&0&1\\1&1&1&0\end{pmatrix}$$

F_7G_1

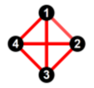

$$\begin{pmatrix}0&1&1&1\\1&0&1&1\\1&1&0&1\\1&1&1&0\end{pmatrix}$$

Molecular constitution 5-atom Class

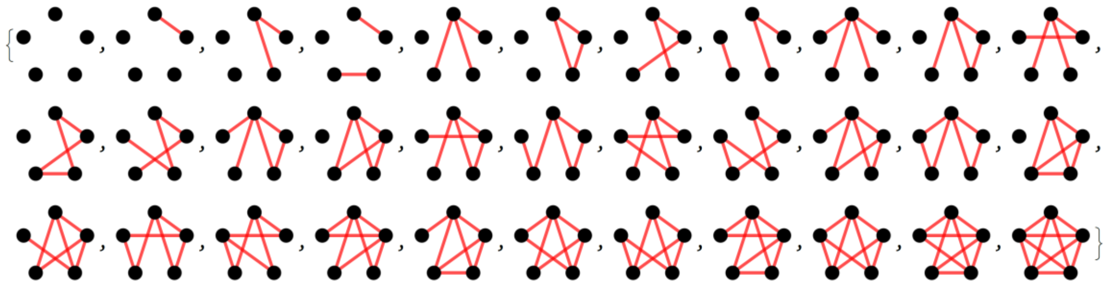

{{1}, {10}, {30, 15}, {20, 10, 60, 30}, {5, 60, 60, 10, 15, 60},
{30, 30, 60, 60, 60, 12}, {60, 15, 5, 60, 60, 10}, {10, 20, 60, 30}, {30, 15}, {10}, {1}}

F_1G_1

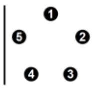

F_2G_1

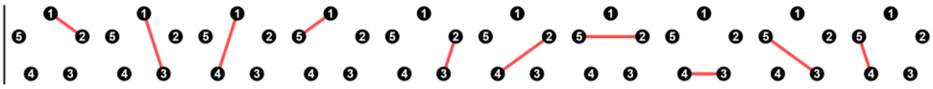

F_3G_1

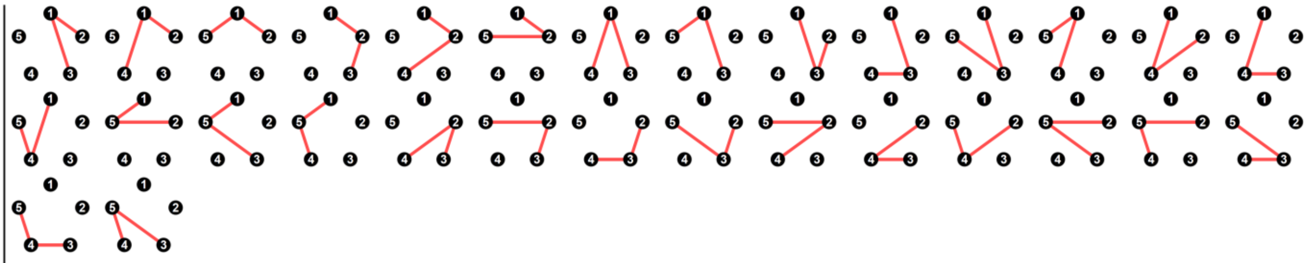

F_3G_2

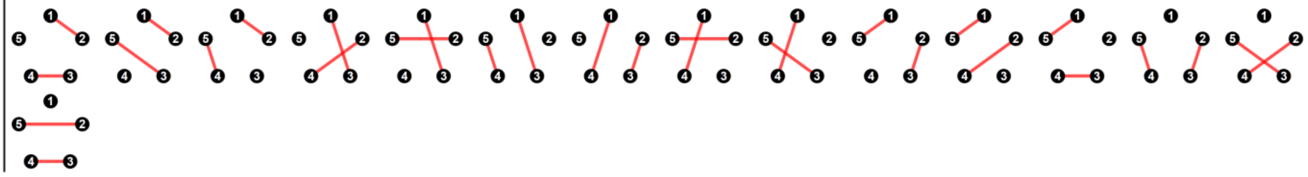

F_4G_1

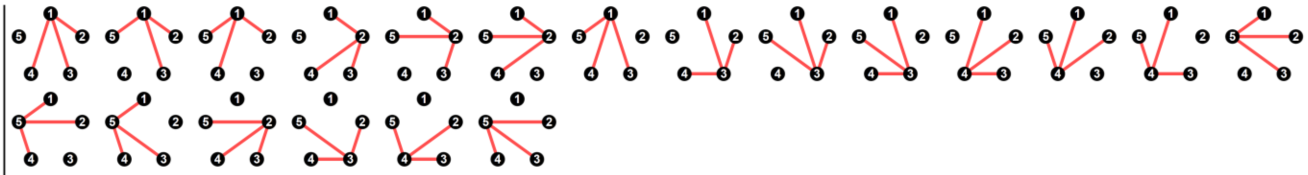

F_4G_2

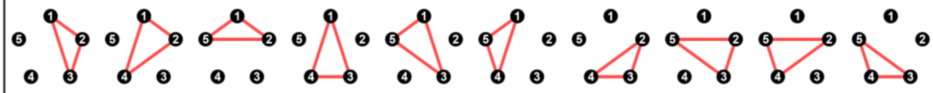

F_4G_3

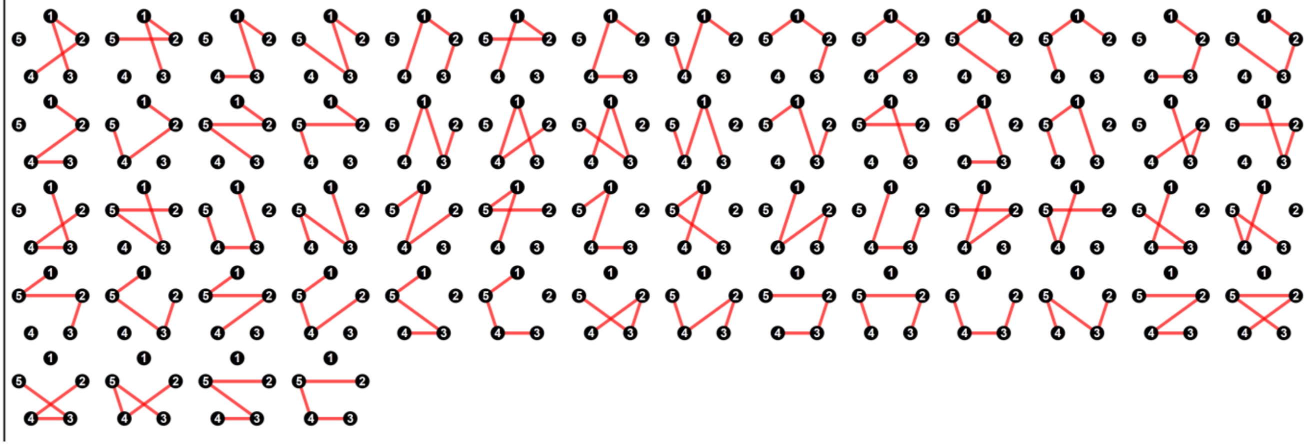

Molecular constitution 5-atom Class continued (1)

F_4G_4

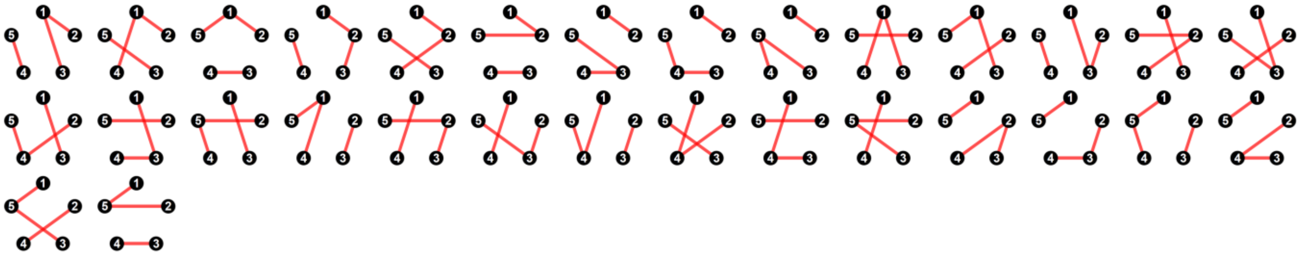

F_5G_1

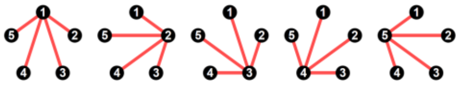

F_5G_2

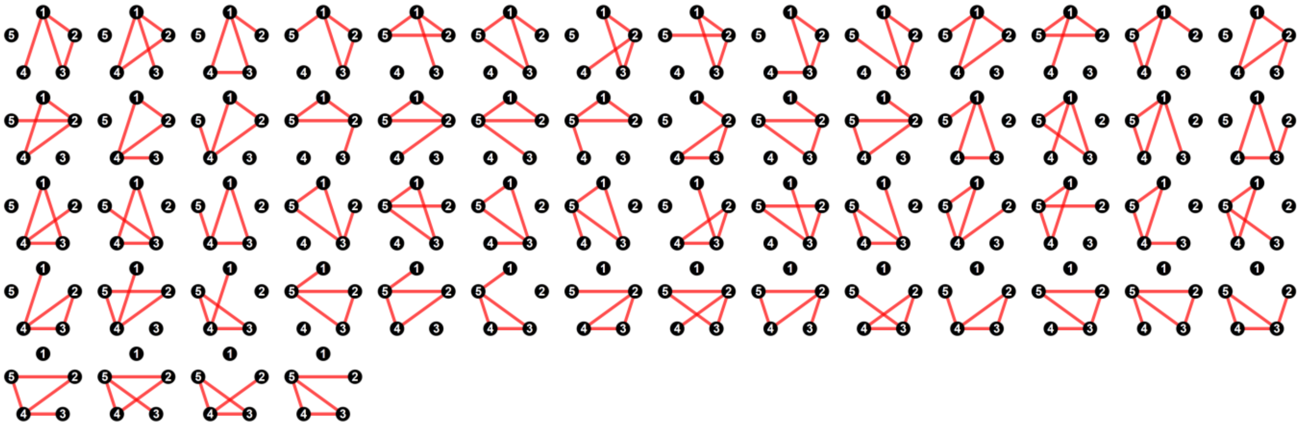

F_5G_3

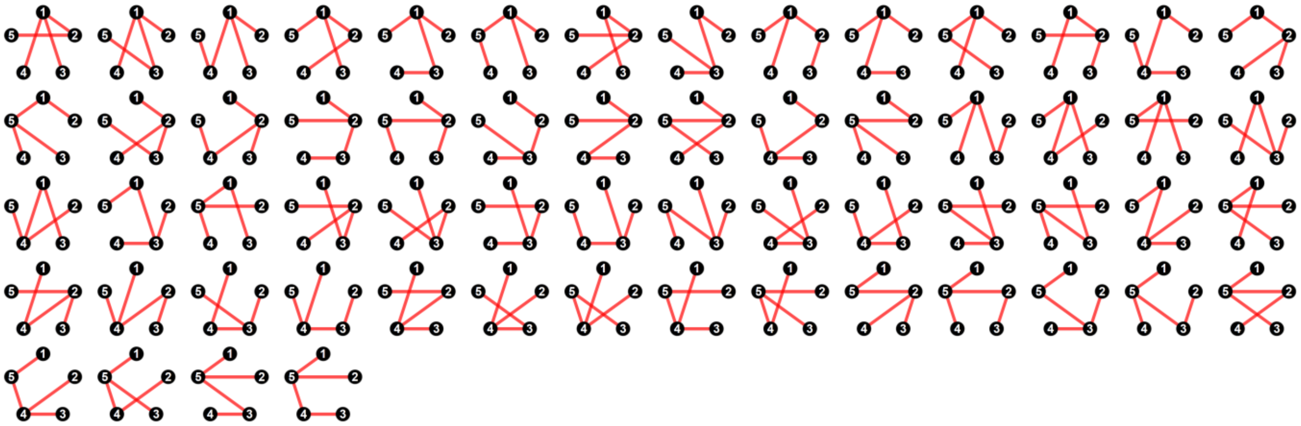

F_5G_4

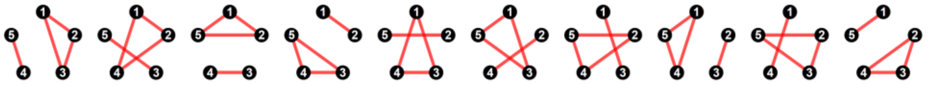

F_5G_5

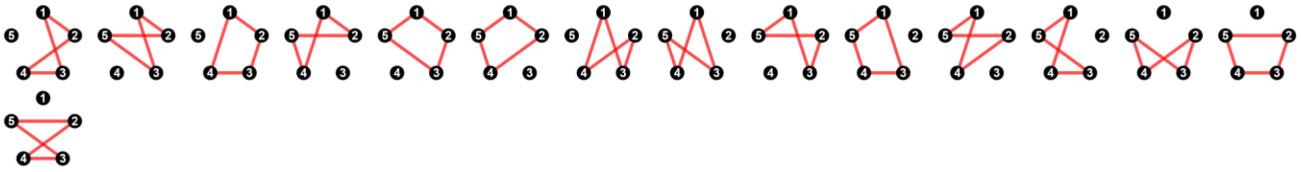

Molecular constitution 5-atom Class continued (2)

F_5G_6

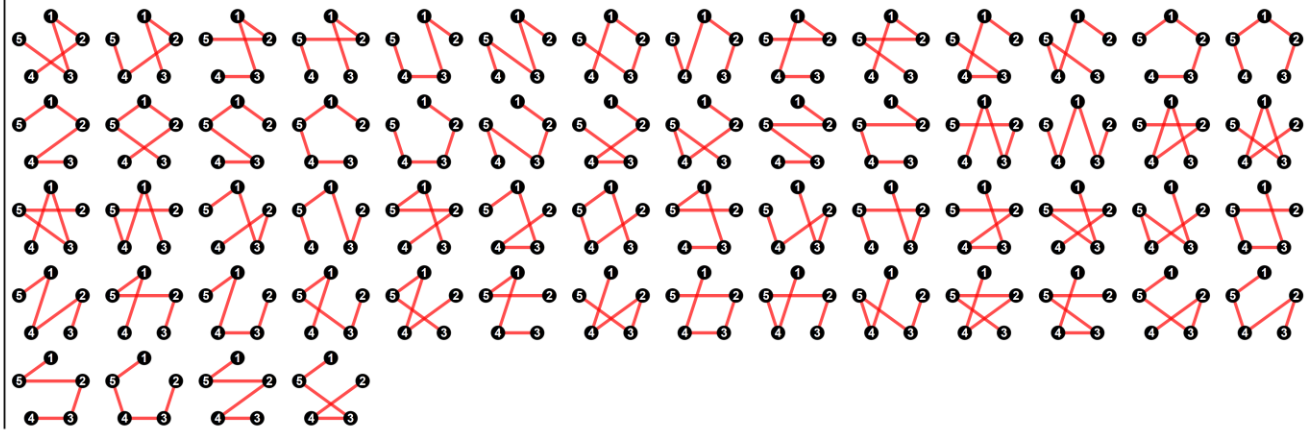

F_6G_1

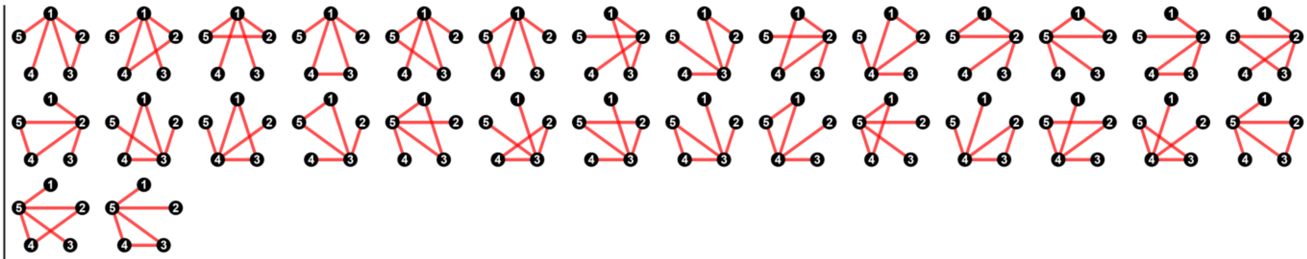

F_6G_2

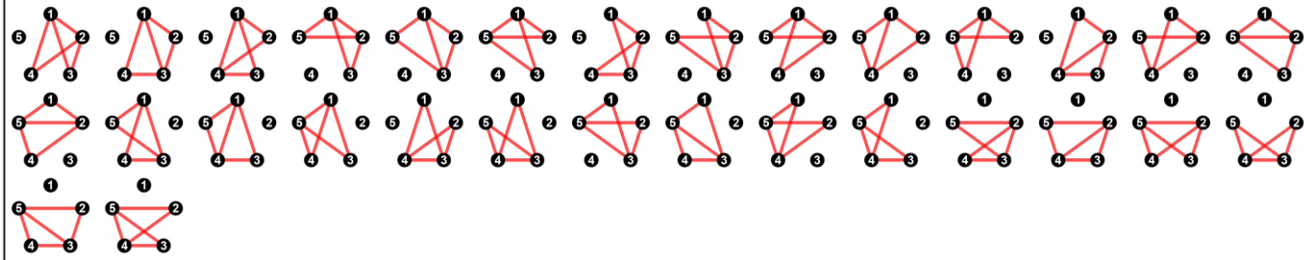

F_6G_3

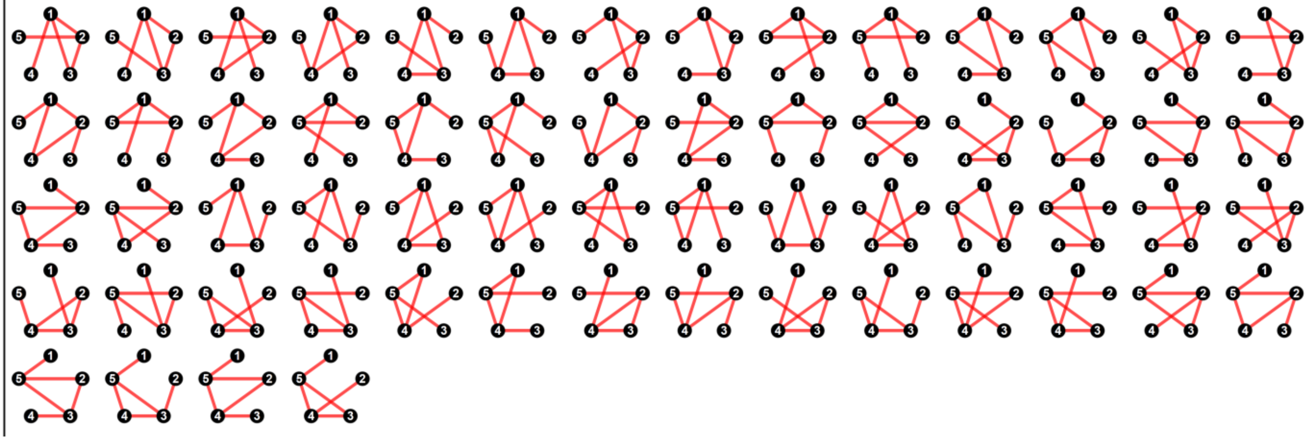

Molecular constitution 5-atom Class continued (3)

F_6G_4

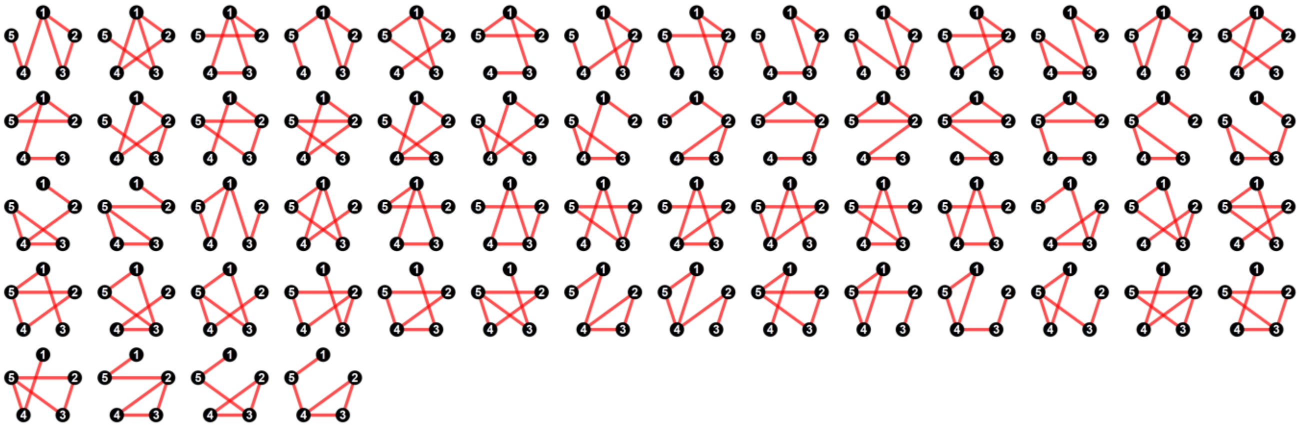

F_6G_5

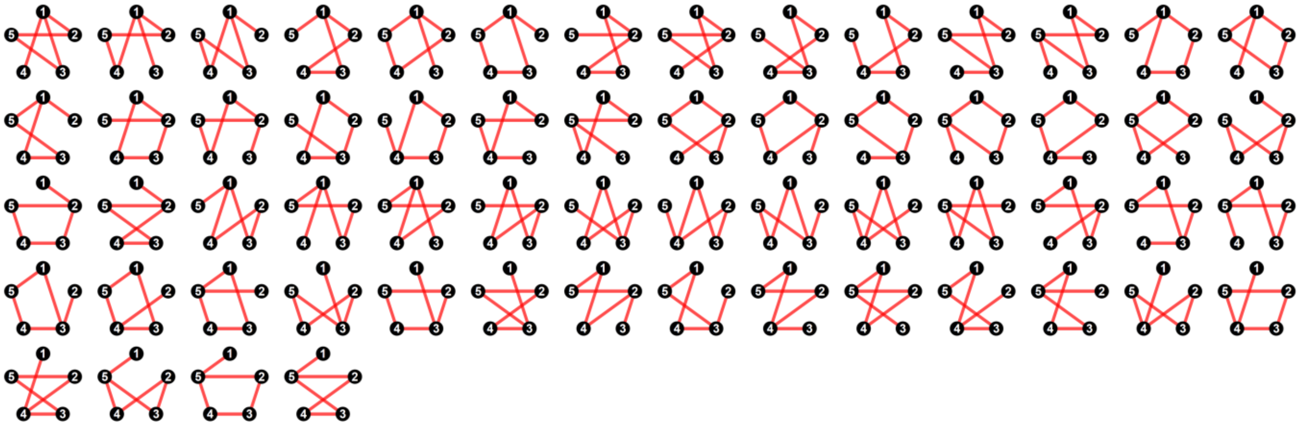

F_6G_6

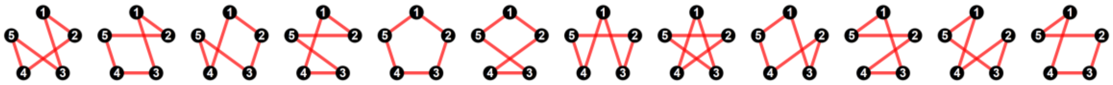

F_7G_1

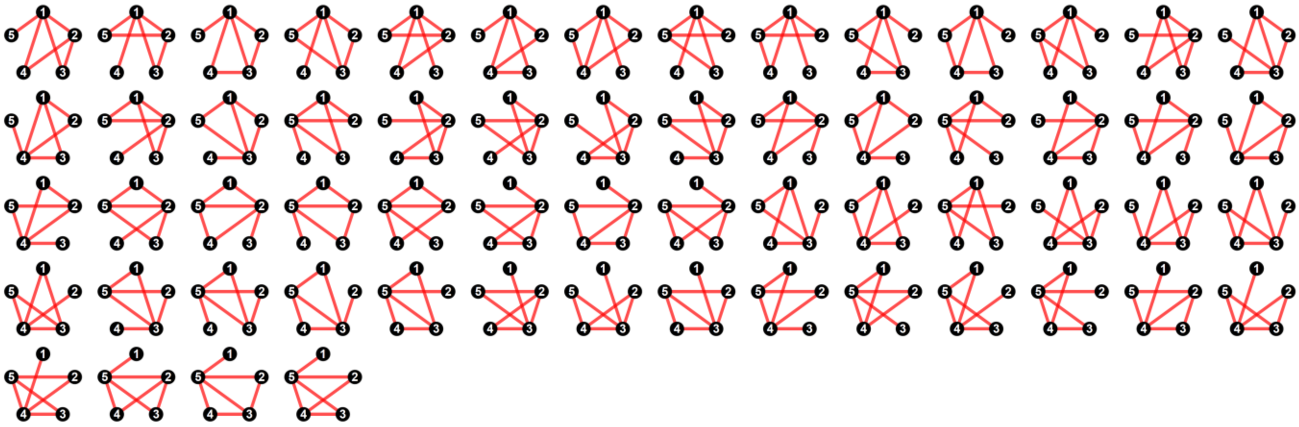

F_7G_2

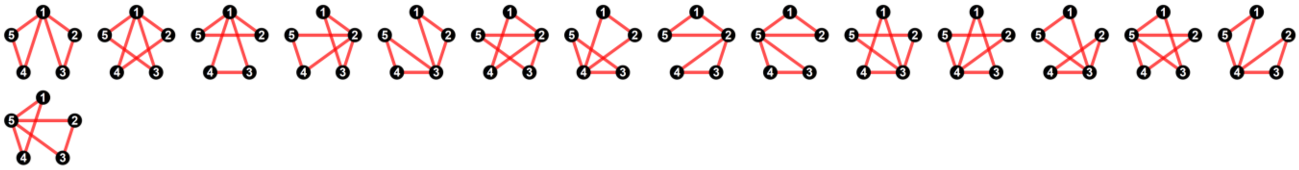

F_7G_3

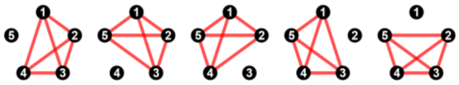

Molecular constitution 5-atom Class continued (4)

F_7G_4

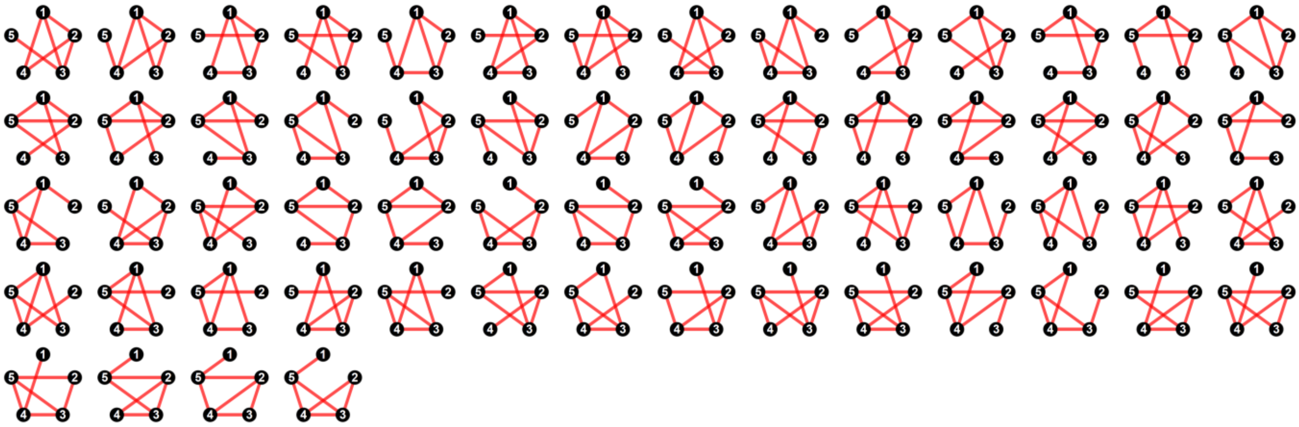

F_7G_5

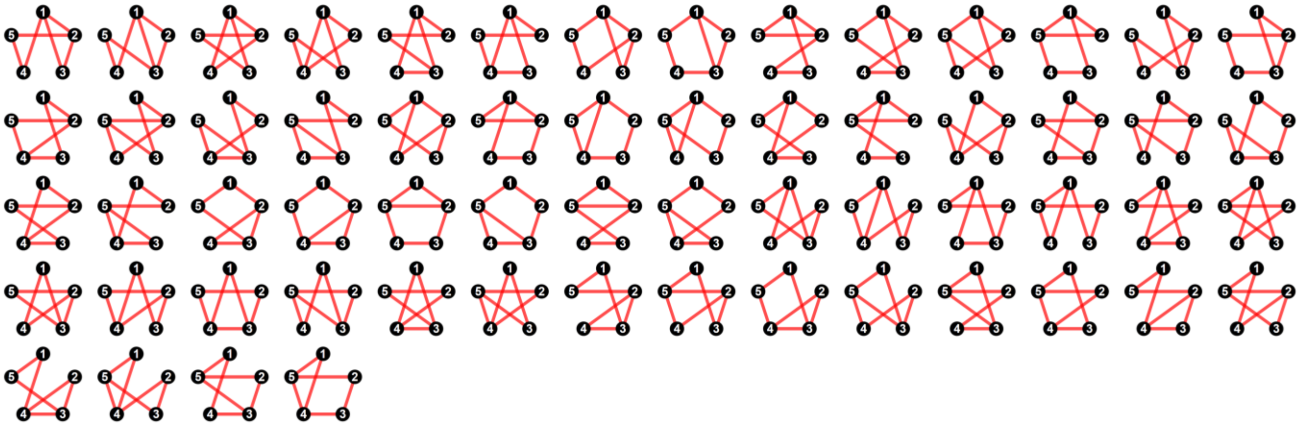

F_7G_6

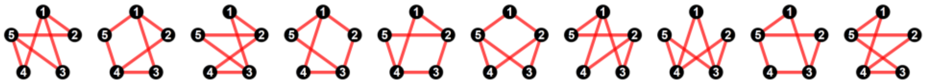

F_8G_1

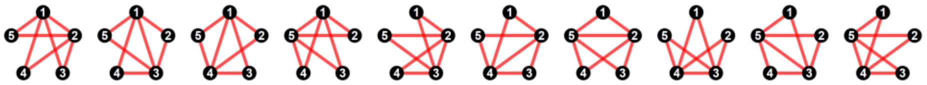

F_8G_2

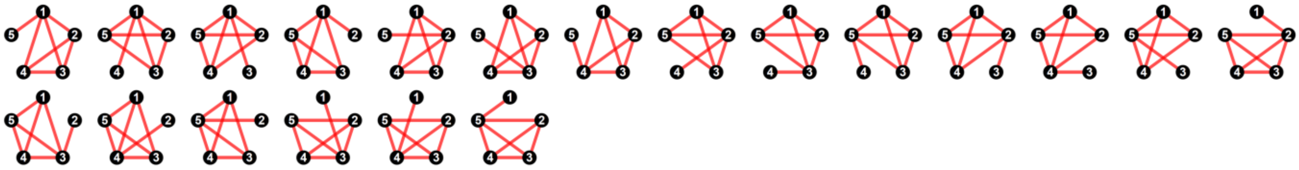

F_8G_3

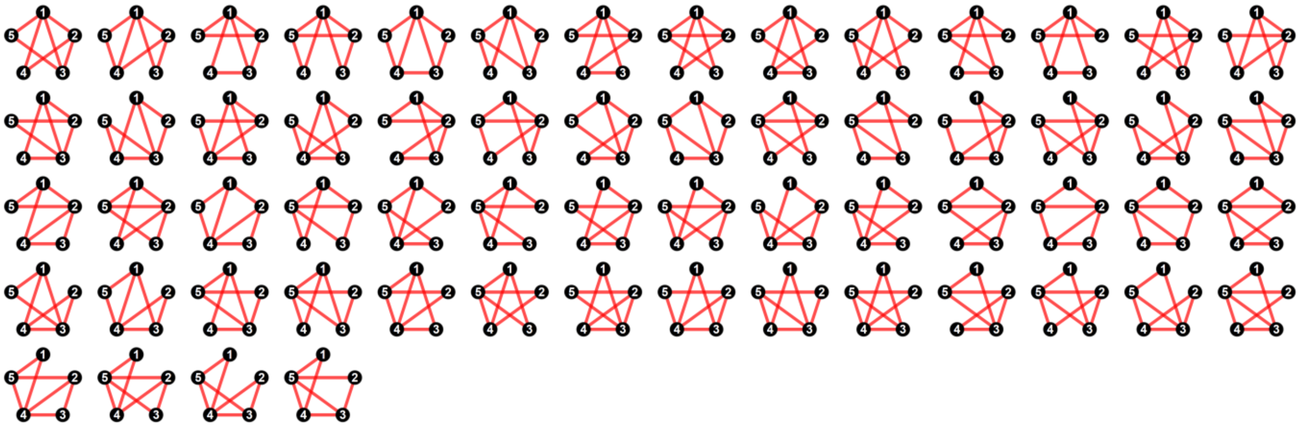

Molecular constitution 5-atom Class continued (5)

F_8G_4

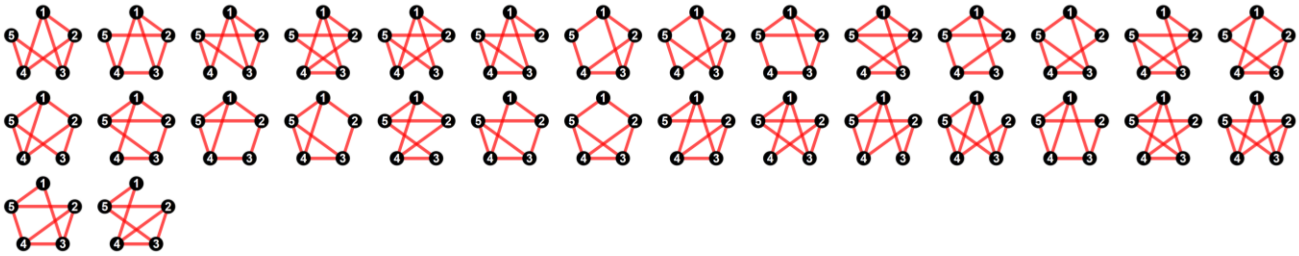

F_9G_1

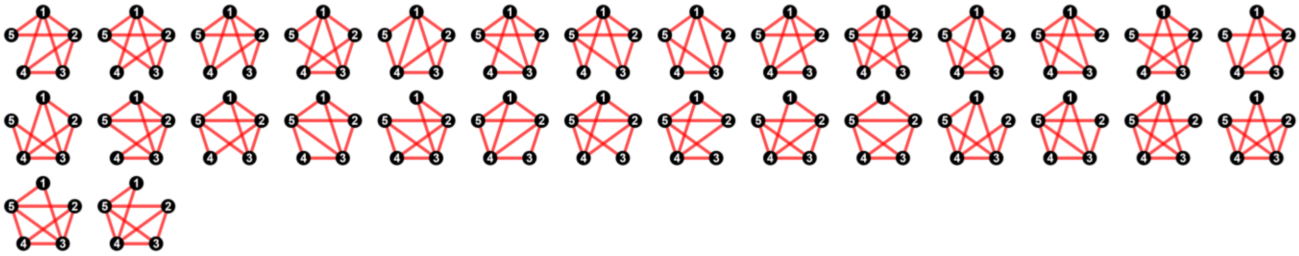

F_9G_2

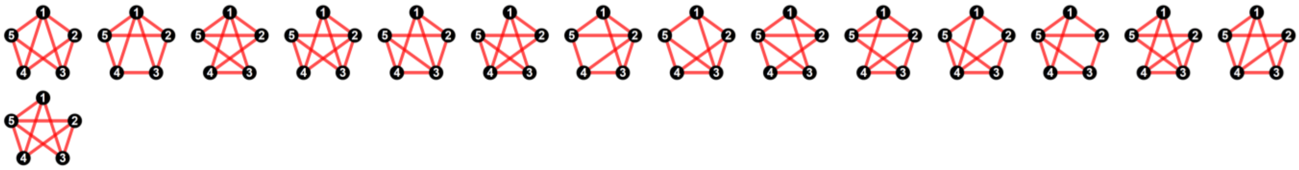

$F_{10}G_1$

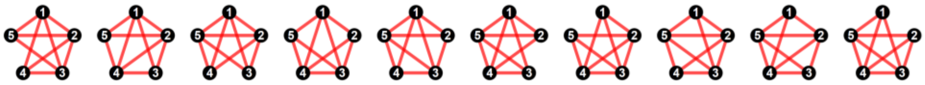

$F_{11}G_1$

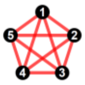

SI10. Fundamental Steric Constraint for otherwise unconstrained *N*-atom-connectivity species

For a set of atoms where the covalent radii are all within the same order of magnitude, it is not possible connect 5 or more atoms together simultaneously. In more formal mathematical language, while the complete graph K_4 is “realisable” as a molecule in $\mathbb{R}^3$, K_5 is not realisable as illustrated below.

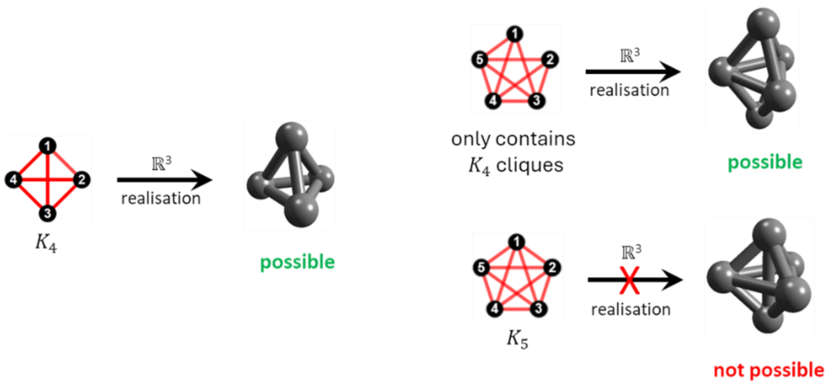

This represents the *fundamental steric constraint* for atom connectivity species.
It then follows that the number of species realisable in $\mathbb{R}^3$ are those species where the *clique number* (number of vertices of the largest *complete* subgraph contained within a graph) is ≤ 4. The graph repository website HouseofGraphs.com (accessed 11 Nov 2025) has a complete listing of simple graph isomorphisms up to $N = 11$. From this site, for each family all isomorphisms with a clique number ≤ 4 were downloaded and the number of $\mathbb{R}^3$ realisable species for each family obtained using:

$$number\ of\ \mathbb{R}^3\ realisable\ species = \sum_i \frac{N!}{|Aut(G_i)|}$$

Where G_i are the clique-number ≤ 4 genera, and $|Aut(G_i)|$ is group order of the automorphism group of G_i. The results are given the the following table.

N	Realisable species in $\mathbb{R}^3$	Non-realisable species in $\mathbb{R}^3$	$\frac{\text{Realisable}}{\text{Non}-\text{realisable}}$
1	1	0	1
2	2	0	1
3	8	0	1
4	64	0	1
5	1 023	1	0.999
6	32 596	1 620 746	0.995
7	476 406	253 308 949	0.227
8	15 126 507	254 517 513	0.056
9	36 671 979	68 682 804 757	0.001
10	1 334 407 550	35 183 037 681 282	3.793×10^{-5}
11	2 521 760 509	36 028 794 497 203 459	7.000×10^{-8}
*12**	*39 569 597 470*	*73 786 976 255 268 608 994*	5.363×10^{-10}

* for $N = 12$, the available data is incomplete hence, both the number and proportion of realisable species listed is an upper limit and the number of non-realisable species a lower limit.

Importantly, the reduction from a superexponetial scaling becomes more apparent with larger N. Indeed, the number of realisable species for $N = 10$ and $N = 11$ suggests a potentially linear scaling beyond this point. The following plots illustrate the trend.

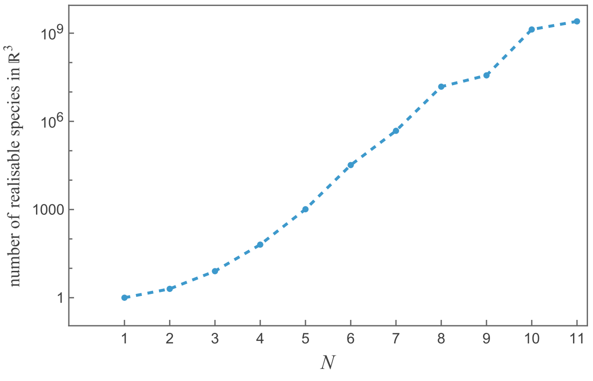

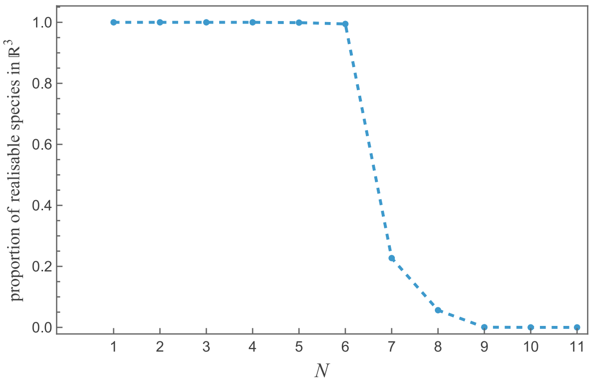

SI11. Compact descriptive symbols for naming species and relationship with chemical nomenclature

A compact descriptive symbol can be constructed by simply writing out the atom-connectivity configuration for each bonder using a “chemical nomenclature-like” form that relies on the IUPAC kappa notation[1] and the site atom locants. If the bonder has a single connection to atom locant *i*, then its description is κ*i*. If a bonder bridges two sites *i* and *j*, then it is written $\kappa^2 i,j$. If the bonder bridges three sites *i*, *j*, and *k* then it is written as $\kappa^3 i,j,k$, and so forth. As an example, the single bonder in species **7a**, being connected to site atoms 16 and 17, would be written as $\kappa^2$16,17. Multiple bonders are represented as colon separated bonder configurations.

Where dissociation is permitted and a bonder is *unbound*, then an underscore is used as a placeholder (i.e., in place of a locant number). With multiple bonders, the atom-connectivity configurations are colon separated. An alternative suggested convention for expressing the atom-connectivity configurations that does not rely on formatted text and the character “κ” is to write out comma-separated locant lists for each bonder in parentheses.

If the potential energy surface (PES) character of a species is known, this too can be incorporated into the descriptive symbol. Potential energy local minimum (LM), transition-state structure (TS), k^{th}-order saddle transition-state structure (*k*S), k^{th}-order inflexion points (*k*I)and non-critical points (NC) are the possibilities. It can be useful to describe NC points as *quasi*-TS (qTS), *quasi*-2S (q2S), etc. For example, modelling of **7a** shows that it represents a first-order transition-state structure and thus its compact descriptive symbol is TS$\kappa^2$16,17. Additional examples from the paper are also given below:

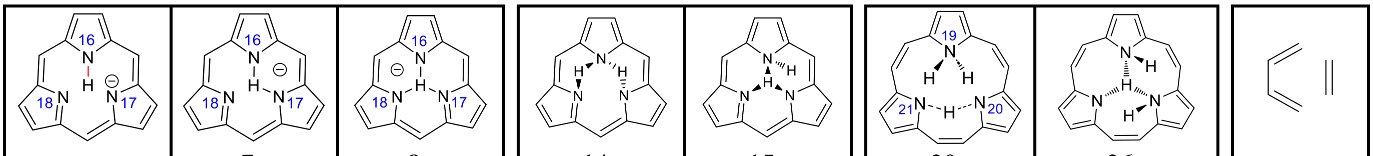

**6a**	**7a**	**8a**	**14a**	**15a**	**20a**	**26a**	**29a**
LMκ16	TS$\kappa^2$16,17	2S$\kappa^3$16,17,18	2S$\kappa^2$16,17:$\kappa^2$16,18	NC$\kappa^3$16,17,18:κ16	TSκ19:κ19:$\kappa^2$20,21	2S$\kappa^3$19,20,21:κ19:κ20	LM_:_

Human-readable symbols have utility beyond simply describing the species. By explicitly listing species’ atom-connectivity configurations, their inter-configuration *relationships* become evident. For example, species **6a** and **7a** are LMκ16 and TS$\kappa^2$16,17, respectively, showing that they only differ by a connection between the bonder and site atom-17. We refer to such a *single* connectivity-configuration difference as “first order” with the $R^{c}_{de}1$ process connecting these two species described as a first-order motion. Similarly, if the differences in atom-connectivity configuration involves two connections (either one bonder’s configuration changing by 2, or two bonders changing by 1 each), this is referred to as “second order”. These terms find application when referring to pathways on the corresponding PESs.

We observe that a first-order relationship generally corresponds to a change of ±1 negative eigenvalues of the species’ Hessian matrix where these values correspond to the associated bond stretches (e.g., LM ⇌ TS [**6a** ⇌ **7a**], or TS ⇌ 2S [**7a** ⇌ **8a**]). Similarly, a second-order relationship typically corresponds to ±2 negative bond-stretching Hessian eigenvalues (e.g., LM ⇌ 2S [**6a** ⇌ **8a**]). This simple correspondence though is not followed for some pericyclic reactions.

To demonstrate how our atom-connectivity descriptors relate to IUPAC systematic names, consider **7a** which has the bonder configuration $\kappa^2$16,17 and is a first-order transition-state structure.The systematic name for **7a** is:

triphyrin[1.1.1]-16,17-diido-$\kappa^2 N^{16,17}$-λ^2-hydronate

where the *bonder* is H^+, formally named hydron, and the *sites* are the three N-atoms.

On inclusion of potential energy surface character into the nomenclatural system, a suggested full name using the explicit assignment of coordination number (here, λ^2 for H) would be:

triphyrin[1.1.1]-16,17-diido-$\kappa^2 N^{16,17}$-λ^2-hydronate(1–)‡

or where the *coordination geometry* (and therefore *implicit* coordination number) of the central hydron is explicitly given:

triphyrin[1.1.1]-16,17-diido-$\kappa^2 N^{16,17}$-(*A*-2)-hydronate(1–)‡.

With the additional inclusion of partial bond order canonicalisation into the nomenclatural system, an alternative suggested full name would be:

desmo[*H*;16,*H*;17]-triphyrin[1.1.1]ate(1–)‡

where the *desmo* list indicates a “partial bond” (denoted by the semicolon) between the hydron locants 16 and 17.

There may exist incidences in which both *desmo* list information and coordination-number or coordination-geometry information are desired which, for this example, it would be:

desmo[*H*;16,*H*;17]-triphyrin[1.1.1]-16,17-diido-$\kappa^2 N^{16,17}$-λ^2-hydronate(1–)‡

desmo[*H*;16,*H*;17]-triphyrin[1.1.1]-16,17-diido-$\kappa^2 N^{16,17}$-(*A*-2)- hydronate(1–)‡

References: 1. N. G. Connelly, T. Damhus, R. Hartshorn and A. T. Hutton, *IUPAC Red Book*, RSC Publishing, London, 2005.

SI12. Modular Structure Theorem

The Modular Structure Theorem states that the reaction graph of configurations given a stated constraint is a reaction *subgraph* of configurations without that constraint. As the fully unconstrained application of the Polytope Formalism (for discretised phenomena) generates a complete graph K_n, the graphs arising from arbitrary constraints are all subgraphs (formally, *induced subgraphs*) of K_n. This is effectively a restatement of standard graph-theoretic definition.

Formal statement

Let $K_n = (V, E)$ be the simple complete graph on vertex set V with $|V| = n$, so that

$$E = \{\{u, v\} \subseteq V : u \neq v\}$$

Let $H = (V', E')$ be any graph obtained from K_n by finitely many operations of:

1. **Vertex deletion**, equivalent to the constraint that specified configurations are to be omitted from the analysis:

choose $x \in V$ and replace (V, E) by $\left(V\backslash\{x\}, E \cap \binom{V\backslash\{x\}}{2}\right)$.

2. **Edge deletion**, equivalent to the constraint that specified transformations are to be omitted from the analysis:

choose $\{u, v\} \in E$ and replace (V, E) by $\left(V, E\backslash\{\{u, v\}\}\right)$.

Then H is a subgraph of K_n; explicitly,

$$V(H) = V' \subseteq V \text{ and } E(H) = E' \subseteq E \cap \binom{V'}{2}.$$

Equivalently,

$$H \leq K_n \text{ where } \left(V(H) \subseteq V(K_n) \; and \; E(H) \subseteq E(K_n)\right).$$

Proof

Each vertex deletion produces a new vertex set $V' \subseteq V$ and restricts edges to pairs from V'; each edge deletion replaces E by a subset. Iterating, we maintain $V' \subseteq V$ and $E' \subseteq E \cap \binom{V'}{2}$. By the definition of subgraph, this is exactly the condition $H \leq K_n$.

References:
Bondy, J. A., & Murty, U. S. R. (1976). *Graph Theory with Applications*. Macmillan. ISBN 0-333-20173-9.
Diestel, R. (2017). *Graph Theory* (5th ed.). Springer. DOI: 10.1007/978-3-662-53622-3
Harary, F. (1969). *Graph Theory*. Addison-Wesley. ISBN 0-201-02787-6.

SI13. Enumeration of families and species under the for $\mathcal{S}_m\mathcal{B}_n$ partitioned approach

The number of species and families for $\mathbb{Z}_m$ bonding-site symmetric $\mathcal{S}_m\mathcal{B}_n$ partitioned systems under the constraint of non-dissociation are:

$$N_s = (2^m - 1)^n$$

and

$$N_f = \binom{m+n-1}{n} = \frac{(m+n-1)!}{(\mathrm{m}-1)!\ n!}$$

More generally, with bonder dissociation permitted, the total number of species N_s, and number of families N_f, are given by:

$$N_s = 2^{mn}$$

and

$$N_f = \binom{m+n}{n} = \frac{(m+n)!}{(\mathrm{m}-1)!\ n!}$$

SI14. Software to generate families, genera, and species for $\mathcal{S}_m\mathcal{B}_n$ with $m, n \leq 8$

Contained in the .zip file is the Fortran source code (authored Prof. Jeffrey R. Reimers, ORCID: 0000-0001-5157-7422) and associated PC executable (for any 32-bit Windows platform) for the determination of the number of families, genera (assuming *non-dissociating* and non-differentiable binders, and $\mathbb{Z}_m$ site symmetry), and species for $\mathcal{S}_m\mathcal{B}_n$ with m, $n \leq 8$. This restriction is caused by the 64-bit encoding used to store the structures internally. When both m and n are large, the requisite computer time and disk-space to store the results, increases superexponentially. Basics checks on the program's accuracy can be made by comparing the total number of families and species to the results from equations above. The program has three modes of use:

genus_direct <m> <n>

produces two ASCII text files named "genera_Dnh_SmBn.txt" listing the families, genera, and number of species per genera, as well as "species_SmBn.txt" containing the families, their genera, and the total number of species in each genus.

genus_direct <m> <n> n

as above, but only the file "species_SmBn.txt" is produced, saving disk space.

genus_direct <m> <n> g

as above but only the file "species_SmBn.txt" is produced, saving disk space, and the number of species per genus is not calculated, saving CPU time.

An abbreviated notation for genera and species is used compared to the IUPAC-consistent kappa notation described in the main text and an earlier SI Section. In this, the κ notation is not applied as this information is also included in the family specification. Again, the notation lists connections from the bonders, specified in order and separated by colons, to the numbered sites. Also, set complement notation flagged by "\" is used when the number of connections exceeds $m/2$. This "hole notation" can be very compact, e.g., the name for genus **17** is simply "\:\" (there are no connection holes as each bonder links to every site, hence no explicit site indices appear in the configuration).

The number of families $N_f = \frac{(m+n-1)!}{(m-1)!\ n!}$, of $\mathbb{Z}_m$ bonding-site symmetric $\mathcal{S}_m\mathcal{B}_n$ partitioned systems under the constraint of non-dissociation is shown in

Table SI.**1**.

Table SI.1 The number of families N_f, of $\mathbb{Z}_m$ bonding-site symmetric $\mathcal{S}_m\mathcal{B}_n$ partitioned systems under the constraint of non-dissociation.

m \ *n*	1	2	3	4	5	6	7	8
1	1	1	1	1	1	1	1	1
2	2	3	4	5	6	7	8	9
3	3	6	10	15	21	28	36	45
4	4	10	20	35	56	84	120	165
5	5	15	35	70	126	210	330	495
6	6	21	56	126	252	462	792	1 287
7	7	28	84	210	462	924	1 716	3 003
8	8	36	120	330	792	1 716	3 432	6 435

The number of genera N_g of $\mathbb{Z}_m$ bonding-site symmetric $\mathcal{S}_m\mathcal{B}_n$ partitioned systems under the constraint of non-dissociation, as determined using the software provided in .zip is shown in Table SI,2.

Table SI.2 The number of genera N_g of $\mathbb{Z}_m$ bonding-site symmetric $\mathcal{S}_m\mathcal{B}_n$ partitioned systems under the constraint of non-dissociation, as determined using the software provided.

m \ n	1	2	3	4	5	6	7	8
1	1	1	1	1	1	1	1	1
2	2	4	6	9	12	16	20	25
3	3	9	23	51	103	196	348	*590*
4	5	28	124	494	1 726	*5 476*	*15 876*	*42 696*
5	7	70	630	4 950	*33 474*	*197 778*		
6	12	224	3 969	*62 100*	*814 826*			
7	17	669	25 725	*817 037*				
8	29	2 269	*177 771*					

a: the number of species in each genus (except for those in blue italics), and their names are also included in the .zip file.

The number of species $N_s = n\,(2m - 1)$ of $\mathbb{Z}_m$ bonding-site symmetric $\mathcal{S}_m\mathcal{B}_n$ partitioned systems under the constraint of non-dissociation is shown in Table SI.3.

Table SI.3 The number of species N_s of $\mathbb{Z}_m$ bonding-site symmetric $\mathcal{S}_m\mathcal{B}_n$ partitioned systems under the constraint of non-dissociation.

m \ n	1	2	3	4	5	6	7	8
1	1	1	1	1	1	1	1	1
2	3	9	27	81	243	729	2 187	6 561
3	7	49	343	2 401	16 807	117 649	823 543	5 764 801
4	15	225	3 375	50 625	759 375	11 390 625	170 859 375	2 562 890 625
5	31	961	29 791	923 521	28 629 151	887 503 681	2.8×10^{10}	8.5×10^{11}
6	63	3969	250 047	15 752 961	992 436 543	6.3×10^{10}	3.9×10^{12}	2.5×10^{14}
7	127	16 129	2 048 383	260 144 641	3.3×10^{10}	4.2×10^{12}	5.3×10^{14}	6.8×10^{16}
8	255	65 025	16 581 375	4 228 250 625	1.1×10^{12}	2.7×10^{14}	7.0×10^{16}	1.8×10^{19}

Table SI.4 The number of families N_f of $\mathbb{Z}_m$ bonding-site symmetric $\mathcal{S}_m\mathcal{B}_n$ partitioned systems under the constraint of non-dissociation when the maximum number of bonds formed to any site is limited to 2.

m \ n	1	2	3	4	5	6	7	8
1	1	1	-	-	-	-	-	-
2	2	3	-	-	-	-	-	-
3	3	6	6	-	-	-	-	-
4	4	10	13	11	-	-	-	-
5	5	15	24	24	18	-	-	-
6	6	21	40	46	39	29	-	-
7	7	28	61	80				-
8	8	36	89					

Table SI.5 The number of genera N_g for $\mathbb{Z}_m$ bonding-site symmetric $\mathcal{S}_m\mathcal{B}_n$ partitioned systems under the constraint of non-dissociation when the maximum number of bonds formed to any site is limited to 2.[a]

m \ n	1	2	3	4	5	6	7	8
1	1	1	-	-	-	-	-	-
2	2	4	-	-	-	-	-	-
3	3	9	10	-	-	-	-	-
4	5	28	57	64	-	-	-	-
5	7	70	281	524	476	-	-	-
6	12	224	1 665	5 160	*7 455*	*5 907*	-	-
7	17	669	9 654	*50 468*				-
8	29	2 269	*59 548*					

a: the number of species in each genus (except for those in italics owing to size limitations), and their names, are also included in .zip.

Table SI.6 The number of species N_s for $\mathbb{Z}_m$ bonding-site symmetric $\mathcal{S}_m\mathcal{B}_n$ partitioned systems under the constraint of non-dissociation when the maximum number of bonds formed to any site is limited to 2.

m \ n	1	2	3	4	5	6	7	8
1	1	1	-	-	-	-	-	-
2	3	9	-	-	-	-	-	-
3	7	49	174	-	-	-	-	-
4	15	225	1 680	6 510	-	-	-	-
5	31	961	13 830	99 840	401 310	-	-	-
6	63	3 969	105 552	1 325 286	9 055 260	369 98 100	-	-
7	127	16 129	774 774	16 290 792				-
8	255	65 025	5 568 960					

Table SI.7 The number of families N_f for $\mathbb{Z}_m$ bonding-site symmetric $\mathcal{S}_m\mathcal{B}_n$ partitioned systems under the constraint of non-dissociation when the maximum number of bonds formed to any site is limited to 1.

m \ n	1	2	3	4	5	6	7	8
1	1	-	-	-	-	-	-	-
2	2	1	-	-	-	-	-	-
3	3	2	1	-	-	-	-	-
4	4	4	2	1	-	-	-	-
5	5	6	4	2	1	-	-	-
6	6	9	7	4	2	1	-	-
7	7	12	11	7	4	2	1	-
8	8	16	16	12	7	4	2	1

Table SI.8 The number of genera N_g for $\mathbb{Z}_m$ bonding-site symmetric $\mathcal{S}_m\mathcal{B}_n$ partitioned systems under the constraint of non-dissociation when the maximum number of bonds formed to any site is limited to 1.

m \ n	1	2	3	4	5	6	7	8
1	1	-	-	-	-	-	-	-
2	2	1	-	-	-	-	-	-
3	3	2	1	-	-	-	-	-
4	5	7	3	1	-	-	-	-
5	7	14	11	3	1	-	-	-
6	12	39	43	20	4	1	-	-
7	17	88	148	92	28	4	1	-
8	29	236	555	500	199	43	5	1

SI15. Formal definition for motions-order relationships between configurations

Within the Polytope Formalism of molecular constitution, the "motions order" relationship $mo_{\{i,j\}}$ for two configurations $\boldsymbol{c}_i = \{b_i^1, b_i^2, \dots, b_i^n\}$ and $\boldsymbol{c}_j = \{b_j^1, b_j^2, \dots, b_j^n\}$ where b_x^k is the connectivity list of bonder x, is defined as:

$$mo_{\{i,j\}} = \sum_{k=1}^{n} \left| b_f^k \setminus b_g^k \right|$$

where

$\{f,g\} \in \{i,j\}$, $\left|b_f^k\right| \geq \left|b_g^k\right|$, and under the *adjacency condition*:

$$\bigwedge_{k=1}^{n} \left(b_i^k \subseteq b_j^k\right) \vee \left(b_j^k \subseteq b_i^k\right)$$

A motions order of zero implies species identity, that is, if $\boldsymbol{c}_i \equiv \boldsymbol{c}_j$ then $mo_{\{i,j\}} = 0$.

The following examples illustrate various scenarios.

Example 1: Comparing the *adjacent* species **10a** (LMκ16:κ17) and **13a** (2Sκ^{2}16,17:κ^{2}16,17) we have $\boldsymbol{c}_{\mathbf{10a}} = \{\{16\},\{17\}\}$ and $\boldsymbol{c}_{\mathbf{13a}} = \{\{16,17\},\{16,17\}\}$, respectively.

Testing for adjacency:

$$\begin{aligned} &(\{16\} \subseteq \{16,17\}) \vee (\{16,17\} \subseteq \{16\}) \wedge (\{17\} \subseteq \{16,17\}) \vee (\{16,17\} \subseteq \{17\}) \\ &= (\text{True} \vee \text{False}) \wedge (\text{True} \vee \text{False}) \\ &= \text{True} \wedge \text{True} = \text{True} \end{aligned}$$

then,

$$\begin{aligned} mo_{\{\mathbf{10a},\mathbf{13a}\}} &= |\{16,17\}\setminus\{16\}| + |\{16,17\}\setminus\{17\}| \\ &= |\{17\}| + |\{16\}| \\ &= 1 + 1 \\ &= 2 \end{aligned}$$

Hence, **10a** and **13a** have a second-order relationship with each other and will *typically* exhibit ±2 negative bond-stretching-related eigenvalues of the Hessian matrix.

Example 2: Comparing the *unbonded* species **39a** (LM_:_) with **40a** (LMκ1:κ4) we have $\boldsymbol{c}_{\mathbf{39a}} = \{\{\},\{\}\}$ and $\boldsymbol{c}_{\mathbf{40a}} = \{\{1\},\{4\}\}$, respectively.

Testing for adjacency:

$$\begin{aligned} &(\{\} \subseteq \{1\}) \vee (\{1\} \subseteq \{\}) \wedge (\{\} \subseteq \{4\}) \vee (\{4\} \subseteq \{\}) \\ &= (\text{True} \vee \text{False}) \wedge (\text{True} \vee \text{False}) \\ &= \text{True} \wedge \text{True} = \text{True} \end{aligned}$$

then,

$$\begin{aligned} mo_{\{\mathbf{39a},\mathbf{40a}\}} &= |\{1\}\setminus\{\}| + |\{4\}\setminus\{\}| \\ &= |\{1\}| + |\{4\}| \\ &= 1 + 1 \\ &= 2 \end{aligned}$$

Example 3: Comparing *nonadjacent* species **27a** (LMκ19:κ19:κ20) and **30a** (TSκ^{2}19,20:κ19:κ21) we have $\boldsymbol{c}_{\mathbf{27a}} = \{\{19\},\{19\},\{20\}\}$ and $\boldsymbol{c}_{\mathbf{30a}} = \{\{\{19{,}20\},\{19\},\{21\}\}\}$, respectively.

Testing for adjacency:

$$\begin{aligned} &(\{19\} \subseteq \{19,20\}) \vee (\{19,20\} \subseteq \{19\}) \wedge (\{19\} \subseteq \{19\}) \vee (\{19\} \subseteq \{19\}) \wedge (\{20\} \subseteq \{21\}) \vee (\{21\} \subseteq \{20\}) \\ &= (\text{True} \vee \text{False}) \wedge (\text{True} \vee \text{True}) \wedge (\text{False} \vee \text{False}) \\ &= \text{True} \wedge \text{True} \wedge \text{False} = \text{False} \end{aligned}$$

Thus, *nonadjacent* species. Nonadjecency can be represented as the Boolean "False", the numerical value −1, or some indicative such as a dash "–".

The accompanying Mathematica graphing software implements this definition for first- and second-order motions graphs. The tabulation of all $mo_{\{i,j\}}$ values give the "motions order matrix" for the entire set of configurations. A Mathematica script ("motions order matrix.nb") for calculating, displaying, and saving such matrices is provided with this Supplementary Information. Outputs (colour-coded matrix plots and numerical matrices) for the $\mathcal{S}_3\mathcal{B}_1$ and $\mathcal{S}_3\mathcal{B}_2$ classes are shown below using the default site-locant numbers 0, 1, and 2:

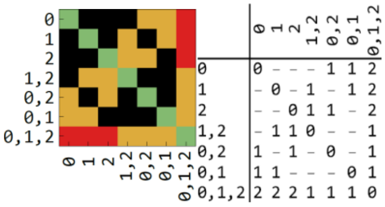

	0	1	2	1,2	0,2	0,1	0,1,2
0	0	-	-	-	1	1	2
1	-	0	-	1	-	1	2
2	-	-	0	1	1	-	2
1,2	-	1	1	0	-	-	1
0,2	1	-	1	-	0	-	1
0,1	1	1	-	-	-	0	1
0,1,2	2	2	2	1	1	1	0

0 1 2

Figure SI.3 The $\mathcal{S}_3\mathcal{B}_1$ class

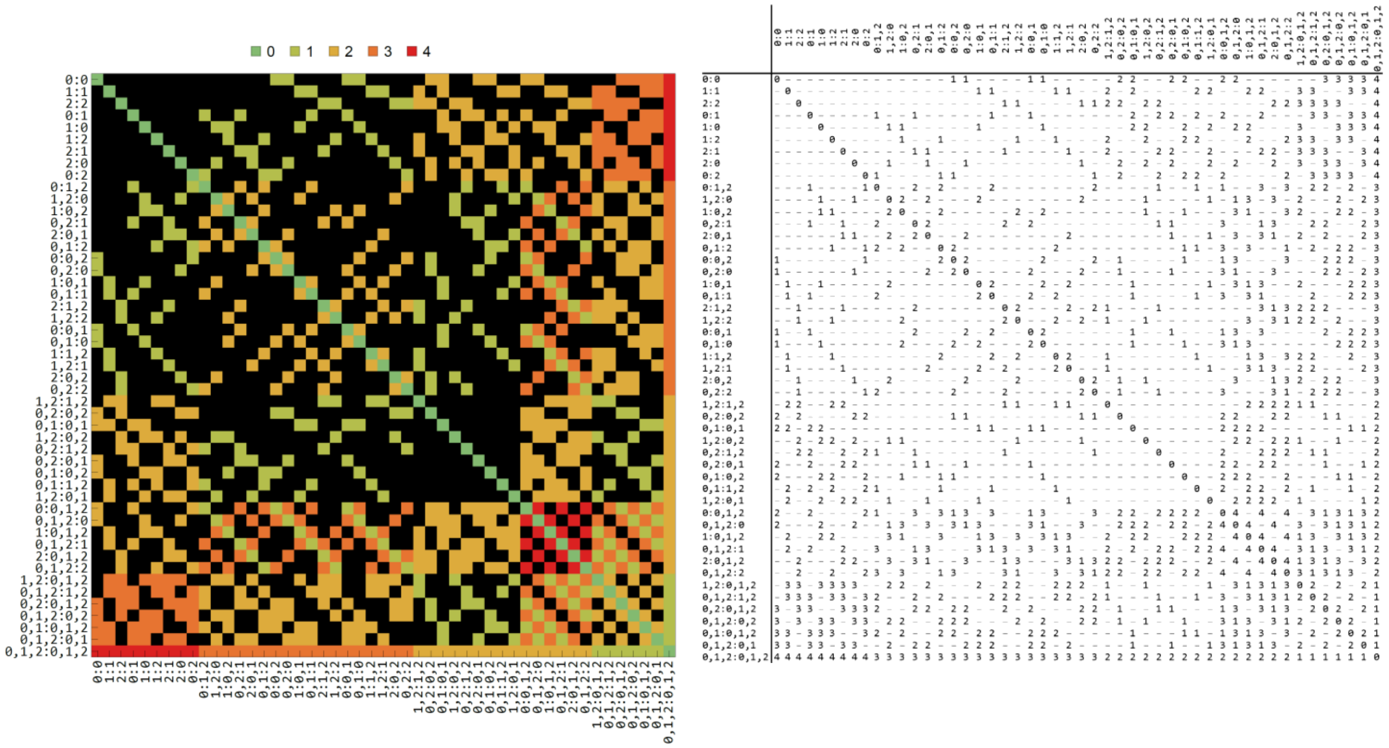

Figure SI.4 The motions-order matrix for the $\mathcal{S}_3\mathcal{B}_2$-partitioned class.

SI16. Species configurations – PES map for free-base subporphyrin partitioned as $\mathcal{S}_3\mathcal{B}_2$ using genera 11 – 16

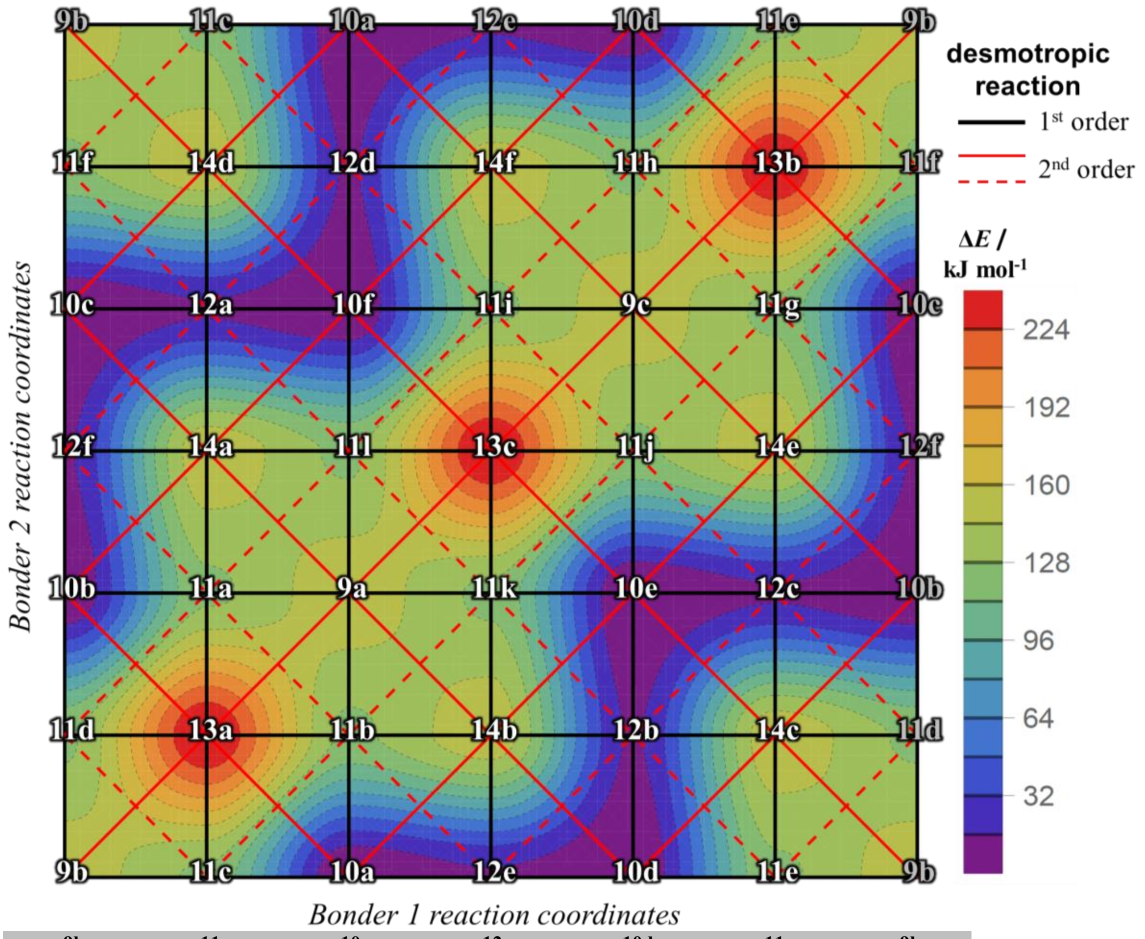

**9b**<br>κ17:κ17<br>*146*<br>**154**	**11c**<br>κ^{2}16,17:κ17<br>*113*<br>**125**	**10a**<br>κ16:κ17<br>*[0]*<br>**[0]**	**12e**<br>κ^{2}16,18:κ17<br>*3*<br>**14**	**10d**<br>κ18:κ17<br>*[0]*<br>**[0]**	**11e**<br>κ^{2}17,18:κ17<br>*113*<br>**125**	**9b**<br>κ17:κ17<br>*146*<br>**154**
**11f**<br>κ17:κ^{2}17,18<br>*113*<br>**125**	**14d**<br>κ^{2}16,17:κ^{2}17,18<br>*139*<br>**155**	**12d**<br>κ16:κ^{2}17,18<br>*3*<br>**14**	**14f**<br>κ^{2}16,18:κ^{2}17,18<br>*139*<br>**155**	**11h**<br>κ18:κ^{2}17,18<br>*113*<br>**125**	**13b**<br>κ^{2}17,18:κ^{2}17,18<br>*211*<br>**240**	**11f**<br>κ17:κ^{2}17,18<br>*113*<br>**125**
**10c**<br>κ17:κ18<br>*[0]*<br>**[0]**	**12a**<br>κ16,κ^{2}17:18<br>*3*<br>**14**	**10f**<br>κ16:κ18<br>*[0]*<br>**[0]**	**11i**<br>κ^{2}16,18:κ18<br>*113*<br>**125**	**9c**<br>κ18:κ18<br>*146*<br>**154**	**11g**<br>κ^{2}17,18:κ18<br>*113*<br>**125**	**10c**<br>κ17:κ18<br>*[0]*<br>**[0]**
**12f**<br>κ17:κ^{2}16,18<br>*3*<br>**14**	**14a**<br>κ^{2}16,17:κ^{2}16,18<br>*139*<br>**155**	**11l**<br>κ16:κ^{2}16,18<br>*113*<br>**125**	**13c**<br>κ^{2}16,18:κ^{2}16,18<br>*211*<br>**240**	**11j**<br>κ18:κ^{2}16,18<br>*113*<br>**125**	**14e**<br>κ^{2}17,18:κ^{2}16,18<br>*139*<br>**155**	**12f**<br>κ17:κ^{2}16,18<br>*3*<br>**14**
**10b**<br>κ17:κ16<br>*[0]*<br>**[0]**	**11a**<br>κ^{2}16,17:κ16<br>*113*<br>**125**	**9a**<br>κ16:κ16<br>*146*<br>**154**	**11k**<br>κ^{2}16,18:κ16<br>*113*<br>**125**	**10e**<br>κ18:κ16<br>*[0]*<br>**[0]**	**12c**<br>κ^{2}17,18:κ16<br>*3*<br>**14**	**10b**<br>κ17:κ16<br>*[0]*<br>**[0]**
**11d**<br>κ17:κ^{2}16,17<br>*113*<br>**125**	**13a**<br>κ^{2}16,17:κ^{2}16,17<br>*211*<br>**240**	**11b**<br>κ16:κ^{2}16,17<br>*113*<br>**125**	**14b**<br>κ^{2}16,18:κ^{2}16,17<br>*139*<br>**155**	**12b**<br>κ18:κ^{2}16,17<br>*3*<br>**14**	**14c**<br>κ^{2}17,18:κ^{2}16,17<br>*139*<br>**155**	**11d**<br>κ17:κ^{2}16,17<br>*113*<br>**125**
**9b**<br>κ17:κ17<br>*146*<br>**154**	**11c**<br>κ^{2}16,17:κ17<br>*113*<br>**125**	**10a**<br>κ16:κ17<br>*[0]*<br>**[0]**	**12e**<br>κ^{2}16,18:κ17<br>*3*<br>**14**	**10d**<br>κ18:κ17<br>*[0]*<br>**[0]**	**11e**<br>κ^{2}17,18:κ17<br>*113*<br>**125**	**9b**<br>κ17:κ17<br>*146*<br>**154**

key:
species
configuration
ΔG / kJ mol^{-1}
***ΔE* / kJ mol^{-1}**

SI17. All genera for tautomerism in two $\mathcal{S}_3\mathcal{B}_2$ systems

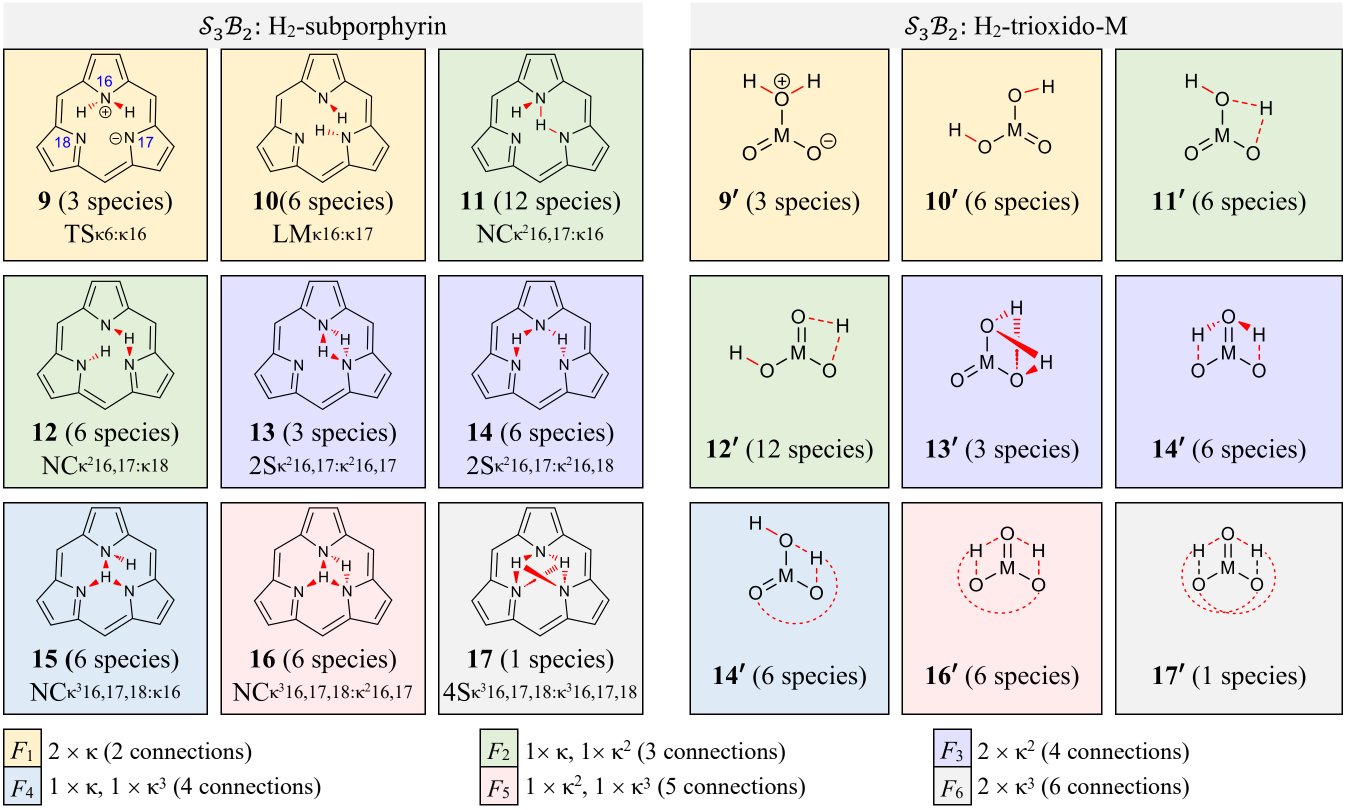

Figure SI.5 All genera for tautomerism in two $\mathcal{S}_3\mathcal{B}_2$ systems (free-base subporphyrin and a general trioxido complex) exhibiting $\mathbb{Z}_3$ $\mathcal{S}$-symmetry under the condition of nondissociation. For each, all N_g = 9 possible genera are shown as 2D chemical structures. Genera are color-coded into N_f = 6 families F_1 through to F_6, depicting different permuted connection totals and bonder linking patterns as described in the legend. Compact labels are given for a representative species of each free-base subporphyrin genus. Stereochemistries are only demonstrative.

SI18. Genera for $\mathcal{S}_2\mathcal{B}_2$ partitioned [dienophile + diene] system allowing for dissociation

The special cases of cycloadditions and chelotropic reactions.

For concerted reactions where (at least) two atom connections form/break simultaneously such is the case for cycloadditions and chelotropic reactions, the list of atom-connectivity configuration species generated by the Polytope Formalism of molecular constitution does not differentiate between the cyclised species and the TS of its formation. An illustrative example is given in Fig. 16a which shows the Diels-Alder reaction of [butadiene + ethene] **29a**, to give cyclohexene **30a**, via the TS **30a′**. The corresponding atom-connectivity picture of these three species are as shown in Fig. 16b. Both **30a′** (TSκ1:κ4) and **30** (LMκ1:κ4) have the same atom-connectivities.

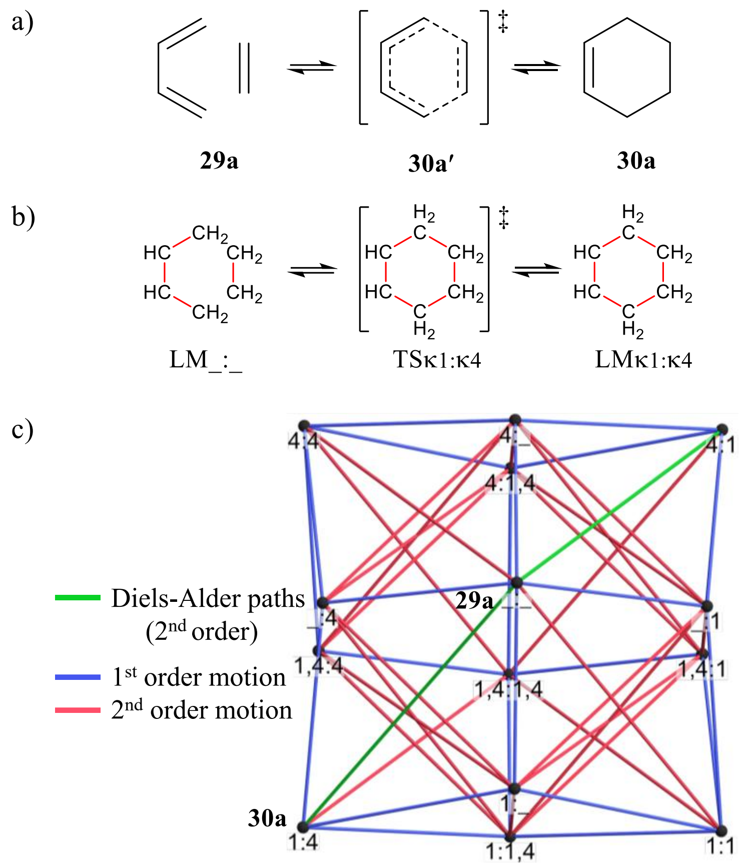

Figure SI.6 The [dienophile + diene] **29a** undergoing [1,4]-cycloaddition to the adduct **30a**. a) Traditional depiction of concerted process including transition-state structure **30a′**. b) Atom-connectivity picture of the reaction does not differentiate between species **30a′** (TSκ1:κ4) and **30a** (LMκ1:κ4). c) First (blue edges) and second (red edges) order motions reaction graph for the system. Second-order paths corresponding to the Diels-Alder reaction (to **30a** LMκ1:κ4, and LMκ4:κ1) are shown in green.

A consequence of this is that the formalism under-samples the PES from the perspective of defining the important species involved. This is not a fatal limitation as knowing of it allows one to look for those additional species of the same configuration but different PES character. For example, when generating initial representative structures, the bonds (atom connections) in question can be constrained by either bond order or an appropriate bond length.

For this chemical system partitioned as $\mathcal{S}_2\mathcal{B}_2$, carbon atoms 1 and 4 of the diene are assigned as the two sites and the carbon atoms (labelled a and b) are assigned as the two bonders. The 16 species are apportioned into the seven genera **SI1** – **SI7** shown, belonging to six families (Figure 2). All families are monogeneric except F_4 which is comprised of genera **SI4** and **39** (**SI5**).

$\mathcal{S}_2\mathcal{B}_2$: “dienophile + diene” system

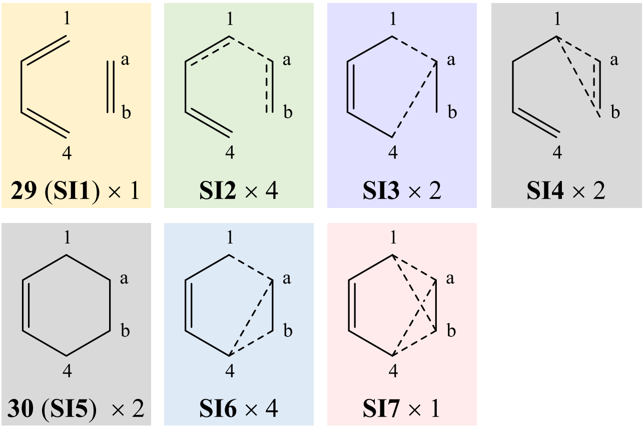

F_1	2 × dissociated	F_2	1 × κ, 1 × dissociated
F_3	1 × κ^2, 1 × dissociated	F_4	2 × κ
F_5	1 × κ^2, 1 × κ	F_6	2 × κ^2

Figure SI.6 All atom-connectivity configuration genera for the analysis of a “dienophile + diene” system partitioned as $\mathcal{S}_2\mathcal{B}_2$ and allowing for dissociation.

SI19. Optimised Cartesian coordinates with key calculation details, results, and systematic chemical names

Density-functional theory calculations were performed using the Gaussian16[1] package using the keywords accompanying the results. Free energies were determined using corrections derived from the simple-harmonic oscillator model at 298.15K.

The following data pages follow the format:

[Genus/structure number]

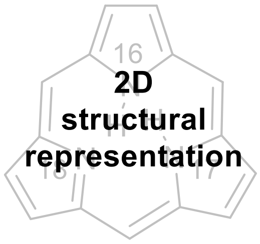

[Atomic number] [cartesian coordinates x1 y1 z1]
[Atomic number] [cartesian coordinates x2 y2 z2]
• • • •
• • • •
• • • •
• • • •
• • • •
• • • •
[Atomic number] [cartesian coordinates x*n* y*n* z*n*]

[Gaussian16 input keywords]

[Gaussian16 output: charge and spin multiplicity]

[Gaussian16 output: full point group]

[Gaussian16 output: final electronic energy]

[Gaussian16 output: frequency lowest energy vibrations]

[Gaussian16 output: sum of electronic and thermal free energies, harmonic approximation at 298.15 K]

Systematic chemical name(s). All non-local-minima structures (transition structures, *etc.*) utilise a proposed transition-structure nomenclature system based upon "canonical partial bond order". Typically, macrocycles are named as formally anionic ligands complexing to one or more hydron (H^+) centres[2], consistent with IUPAC inorganic nomenclature ("Red Book") recommendations.[3]

References:

1 Frisch, M. J.; Trucks, G. W.; Schlegel, H. B.; Scuseria, G. E.; Robb, M. A.; Cheeseman, J. R.; Scalmani, G.; Barone, V.; Petersson, G. A.; Nakatsuji, H.; Li, X.; Caricato, M.; Marenich, A. V.; Bloino, J.; Janesko, B. G.; Gomperts, R.; Mennucci, B.; Hratchian, H. P.; Ortiz, J. V.; Izmaylov, A. F.; Sonnenberg, J. L.; Williams; Ding, F.; Lipparini, F.; Egidi, F.; Goings, J.; Peng, B.; Petrone, A.; Henderson, T.; Ranasinghe, D.; Zakrzewski, V. G.; Gao, J.; Rega, N.; Zheng, G.; Liang, W.; Hada, M.; Ehara, M.; Toyota, K.; Fukuda, R.; Hasegawa, J.; Ishida, M.; Nakajima, T.; Honda, Y.; Kitao, O.; Nakai, H.; Vreven, T.; Throssell, K.; Montgomery Jr., J. A.; Peralta, J. E.; Ogliaro, F.; Bearpark, M. J.; Heyd, J. J.; Brothers, E. N.; Kudin, K. N.; Staroverov, V. N.; Keith, T. A.; Kobayashi, R.; Normand, J.; Raghavachari, K.; Rendell, A. P.; Burant, J. C.; Iyengar, S. S.; Tomasi, J.; Cossi, M.; Millam, J. M.; Klene, M.; Adamo, C.; Cammi, R.; Ochterski, J. W.; Martin, R. L.; Morokuma, K.; Farkas, O.; Foresman, J. B.; Fox, D. J. *Gaussian 16 Rev. C.01*, 2016.

6a

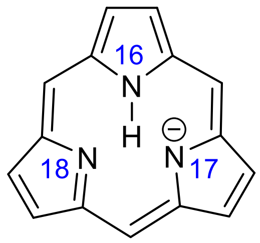

C	-1.9823	0.2505	1.2433
C	-2.2577	-0.4824	2.4822
C	-1.0743	-0.5239	3.1885
C	-0.0855	0.1863	2.3749
C	1.3310	0.0390	2.3496
C	2.0545	0.1642	1.1402
C	3.3042	-0.4102	0.6942
C	3.3042	-0.4102	-0.6942
C	2.0545	0.1642	-1.1402
C	1.3310	0.0390	-2.3496
C	-0.0855	0.1863	-2.3749
C	-1.0743	-0.5239	-3.1885
C	-2.2577	-0.4824	-2.4822
C	-1.9823	0.2505	-1.2433
C	-2.6605	0.1379	0.0000
N	-0.7156	0.7335	1.3062
N	1.4395	0.6131	0.0000
N	-0.7156	0.7335	-1.3062
H	-3.1857	-0.9821	2.7359
H	-0.8816	-1.0638	4.1083
H	4.0571	-0.8468	1.3380
H	4.0571	-0.8468	-1.3380
H	-0.8816	-1.0638	-4.1083
H	-3.1857	-0.9821	-2.7359
H	1.8349	-0.4103	3.2001
H	1.8349	-0.4103	-3.2001
H	-3.6456	-0.3227	0.0000
H	0.4224	0.8573	0.0000

#P b3lyp/6-31+g** empiricaldispersion=gd3bj symm=(veryloose,follow) integral=ultrafinegrid geom=check guess=read opt=(maxstep=3,modredundant,tight) freq scrf=(solvent=chloroform,read) scf=(xqc,maxconventionalcycles=40)

Charge = -1 Multiplicity = 1

Full point group CS NOp 2

E(RB3LYP) = -742.047761126

Frequencies -- 114.1096 119.5232 132.6605

Sum of electronic and thermal Free Energies= -741.873538

16*H*-triphyrin[1.1.1]ate

7a

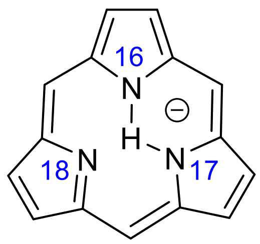

C	-0.2010	-0.0802	-2.3026
C	0.3845	-1.0550	-3.2105
C	0.4545	-2.2637	-2.5395
C	-0.0921	-2.0446	-1.2124
C	0.0604	-2.7330	0.0221
C	-0.0920	-2.0240	1.2450
C	0.4544	-2.2215	2.5755
C	0.3836	-1.0022	3.2270
C	-0.2023	-0.0424	2.3035
C	-0.1252	1.3721	2.2924
C	-0.2597	2.1112	1.0845
C	0.4546	3.3211	0.6628
C	0.4556	3.3093	-0.7159
C	-0.2582	2.0927	-1.1182
C	-0.1228	1.3342	-2.3138
N	-0.5738	-0.7675	-1.1845
N	-0.5742	-0.7477	1.1963
N	-0.7675	1.5032	-0.0122
H	0.7717	-0.8421	-4.1999
H	0.9053	-3.1798	-2.9014
H	0.9057	-3.1315	2.9516
H	0.7707	-0.7730	4.2127
H	0.9684	4.0235	1.3090
H	0.9709	4.0002	-1.3733
H	0.4883	-3.7304	0.0307
H	0.2912	1.8626	3.1686
H	0.2950	1.8107	-3.1970
H	-0.7260	-0.3994	0.0030

#P b3lyp/6-31+g** empiricaldispersion=gd3bj symm=(veryloose,follow) integral=ultrafinegrid geom=check guess=read opt=(ts,calcall,maxstep=7,tight) freq scrf=(solvent=chloroform,read) scf=(save,intrep,xqc,maxconventionalcycles=50)

Charge = -1 Multiplicity = 1

Full point group C1 NOp 1

E(RB3LYP) = -742.047761126

Frequencies -- -1148.3611 113.3209 129.4563

Sum of electronic and thermal Free Energies= -741.871203

---

triphyrin[1.1.1]-16,17-diido-κ^2*N*^{16,17}-λ^2-hydronate(1–)‡

triphyrin[1.1.1]-16,17-diido-κ^2*N*^{16,17}-(*A*-2)-hydronate(1–)‡

desmo[*H*;16,*H*;17]-triphyrin[1.1.1]ate(1–)‡

desmo[*H*;16,*H*;17]-triphyrin[1.1.1]ate(1–)‡

8a

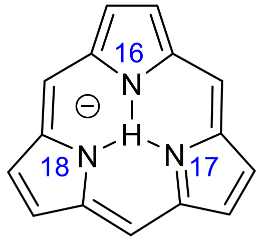

C	-0.0655	-0.1716	2.3476
C	-1.0704	0.3965	3.2379
C	-2.2679	0.3966	2.5462
C	-1.9993	-0.1709	1.2304
C	-2.6969	-0.0534	0.0000
C	-1.9993	-0.1709	-1.2304
C	-2.2679	0.3966	-2.5462
C	-1.0704	0.3965	-3.2379
C	-0.0655	-0.1716	-2.3476
C	1.3491	-0.0552	-2.3364
C	2.0659	-0.1703	-1.1169
C	3.3353	0.4065	-0.6915
C	3.3353	0.4065	0.6915
C	2.0659	-0.1703	1.1169
C	1.3491	-0.0552	2.3364
N	-0.7066	-0.5942	1.2249
N	-0.7066	-0.5942	-1.2249
N	1.4169	-0.5980	0.0000
H	-0.8899	0.8151	4.2211
H	-3.2098	0.8144	2.8819
H	-3.2098	0.8144	-2.8819
H	-0.8899	0.8151	-4.2211
H	4.0923	0.8327	-1.3395
H	4.0923	0.8327	1.3395
H	-3.7087	0.3412	0.0000
H	1.8552	0.3384	-3.2130
H	1.8552	0.3384	3.2130
H	0.0034	-0.6954	0.0000

#P b3lyp/6-31+g** empiricaldispersion=gd3bj symm=(veryloose,follow) integral=ultrafinegrid geom=check guess=read opt=(ts,calcfc,saddle=2,maxstep=2,tight) freq scrf=(solvent=chloroform,read) scf=(xqc,maxconventionalcycles=40)

Charge = -1 Multiplicity = 1

Full point group CS NOp 2

SCF Done: E(RB3LYP) = -742.032503022 A.U. after 5 cycles

Frequencies -- -1399.2028 -1384.8909 115.9554

Sum of electronic and thermal Free Energies= -741.865297

---

triphyrin[1.1.1]-16,17-diido-$\kappa^3N^{16,17,18}$-λ^3-hydronate(1–)$‡^2$

triphyrin[1.1.1]-16,17-diido-$\kappa^3N^{16,17,18}$-(*TP*-3)-hydronate(1–)$‡^2$

desmo[*H*;16,*H*;17,*H*;18]-triphyrin[1.1.1]ate(1–)$‡^2$

desmo[*H*;16,*H*;17,*H*;18]-triphyrin[1.1.1]ate(1–)$‡^2$

trivial geometric deviation from C_{3v} symmetry due to the solvent cavity

9

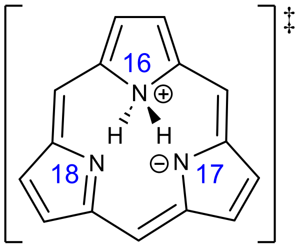

C	0.1914	-1.1854	2.0708
C	0.1122	-0.6926	3.4009
C	-0.1122	0.6926	3.4009
C	-0.1914	1.1854	2.0708
C	-0.3951	2.3646	1.3504
C	-0.4597	2.3287	-0.0886
C	-0.1068	3.3046	-1.1174
C	0.0000	2.6097	-2.3013
C	-0.2616	1.2045	-1.9944
C	0.0000	0.0000	-2.7009
C	0.2616	-1.2045	-1.9944
C	0.0000	-2.6097	-2.3013
C	0.1068	-3.3046	-1.1174
C	0.4597	-2.3287	-0.0886
C	0.3951	-2.3646	1.3504
N	0.0000	0.0000	1.2737
N	-0.6358	1.1356	-0.6920
N	0.6358	-1.1356	-0.6920
H	0.2234	-1.2966	4.2919
H	-0.2234	1.2966	4.2919
H	0.0924	4.3574	-0.9608
H	0.3093	3.0067	-3.2601
H	-0.3093	-3.0067	-3.2601
H	-0.0924	-4.3574	-0.9608
H	0.7415	0.0052	0.5112
H	-0.7415	-0.0052	0.5112
H	-0.4240	3.3118	1.8787
H	0.0000	0.0000	-3.7883
H	0.4240	-3.3118	1.8787

#P b3lyp/6-31g* empiricaldispersion=gd3bj symm=(veryloose,follow) integral=ultrafinegrid geom=check guess=read opt=(ts,calcall,tight) freq scrf=(solvent=chloroform,read) scf=(xqc,maxconventionalcycles=40)

Charge = 0 Multiplicity = 1

Full point group C2 NOp 2

E(RB3LYP) = -742.441287398

Frequencies -- -326.3840 99.0656 111.3405

Sum of electronic and thermal Free Energies= -742.254162

16*H*,16*H*-triphyrin[1.1.1]‡

10

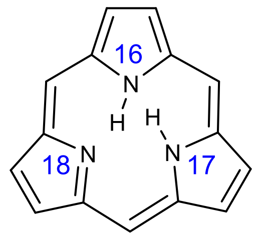

C	1.8582	-1.2087	-0.8786
C	2.5476	-0.9461	-2.1201
C	1.6502	-0.3694	-3.0023
C	0.3753	-0.2422	-2.3351
C	-0.7912	0.5211	-2.5670
C	-1.5800	0.9632	-1.4715
C	-2.9679	1.2704	-1.2437
C	-3.1784	1.3693	0.1263
C	-1.9308	1.1201	0.7980
C	-1.5152	0.8305	2.1228
C	-0.3417	0.0611	2.3335
C	0.6338	0.0070	3.4214
C	1.7424	-0.6400	2.9287
C	1.4413	-1.0090	1.5445
C	2.2816	-1.3852	0.4587
N	0.5479	-0.9029	-1.1435
N	-1.0248	1.0150	-0.2242
N	0.1606	-0.6528	1.2932
H	3.6054	-1.0978	-2.2886
H	1.8762	0.0211	-3.9853
H	-3.7245	1.3280	-2.0144
H	-4.1308	1.5137	0.6182
H	0.5320	0.4732	4.3935
H	2.6809	-0.8039	3.4440
H	-0.0947	-0.9335	-0.3396
H	-0.0521	0.8503	-0.0308
H	-1.0645	0.7983	-3.5792
H	-2.0868	1.2124	2.9624
H	3.3217	-1.6294	0.6462

#P b3lyp/6-31g* empiricaldispersion=gd3bj symm=(veryloose,follow) integral=ultrafinegrid geom=check guess=read opt=(calcall,maxstep=2,tight) freq scrf=(solvent=chloroform,read) scf=(xqc,maxconventionalcycles=40)

Charge = 0 Multiplicity = 1

Full point group C1 NOp 1

SCF Done: E(RB3LYP) = -742.500044526 A.U. after 6 cycles

Frequencies -- 89.2699 116.0102 139.3767

Sum of electronic and thermal Free Energies= -742.309585

16*H*,17*H*-triphyrin[1.1.1]

11

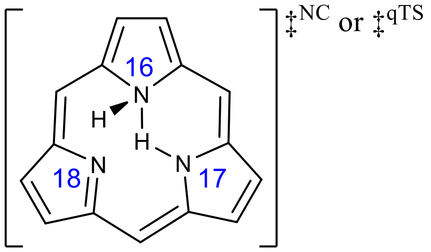

C	0.0573	-2.3362	0.1455
C	1.0803	-3.1495	-0.4118
C	2.2957	-2.4644	-0.4280
C	2.1286	-1.1695	0.1261
C	2.7274	0.0766	-0.0504
C	1.9880	1.2933	0.0958
C	2.1777	2.6086	-0.4913
C	0.9518	3.2391	-0.4343
C	0.0164	2.3120	0.1878
C	-1.3998	2.2606	0.1170
C	-2.1229	1.0551	0.2735
C	-3.3275	0.6501	-0.4631
C	-3.3070	-0.7182	-0.5107
C	-2.0975	-1.1288	0.2066
C	-1.3289	-2.3186	0.0294
N	0.7728	-1.1856	0.6924
N	0.7230	1.2155	0.5738
N	-1.5113	-0.0518	0.7588
H	0.9008	-4.1059	-0.8832
H	3.1920	-2.8049	-0.9256
H	3.0839	2.9851	-0.9466
H	0.7078	4.2172	-0.8352
H	-4.0315	1.3154	-0.9535
H	-3.9888	-1.3698	-1.0453
H	0.8094	-1.2563	1.7167
H	3.7108	0.1061	-0.5073
H	-1.9116	3.1172	-0.3150
H	-1.7909	-3.1813	-0.4386
H	0.3883	-0.1313	0.6053

#P b3lyp/6-31g* empiricaldispersion=gd3bj symm=(veryloose,follow) integral=ultrafinegrid guess=read freq scrf=(solvent=chloroform,read) scf=(save,intrep,xqc,maxconventionalcycles=50)

Charge = 0 Multiplicity = 1

Full point group C1 NOp 1

SCF Done: E(RB3LYP) = -742.452437104 A.U. after 12 cycles

Frequencies -- -580.1492 115.1427 128.1582

Sum of electronic and thermal Free Energies= -742.266557

---

triphyrin[1.1.1]-16,17-diido-1κ$^2N^{16,17}$,2κN^{16}-1λ^2-dihydron‡^{NC}

triphyrin[1.1.1]-16,17-diido-1κ$^2N^{16,17}$,2κN^{16}-1λ^2-dihydron‡^{qTS}

triphyrin[1.1.1]-16,17-diido-1κ$^2N^{16,17}$,2κN^{16}-1-(*A*-2)-dihydron‡^{NC}

triphyrin[1.1.1]-16,17-diido-1κ$^2N^{16,17}$,2κN^{16}-1-(*A*-2)-dihydron‡^{qTS}

16*H*,*desmo*[*H*^{1};16,*H*^{1};17]-triphyrin[1.1.1]‡^{NC}

16*H*,*desmo*[*H*^{1};16,*H*^{1};17]-triphyrin[1.1.1]‡^{qTS}

16*H*,*desmo*[*H*^{1};16,*H*^{1};17]-triphyrin[1.1.1]‡^{NC}

16*H*,*desmo*[*H*^{1};16,*H*^{1};17]-triphyrin[1.1.1]‡^{qTS}

non-critical point on the PES - has some of the character of a transition-state structure (a quasi-transition-state structure) but is not a true first-order saddle point

12

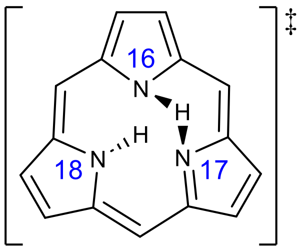

C	-0.1810	1.1280	2.0932
C	0.2390	0.6584	3.3855
C	0.2392	-0.7315	3.3702
C	-0.1808	-1.1727	2.0680
C	-0.1041	-2.3807	1.3287
C	0.0467	-2.3344	-0.0773
C	-0.2611	-3.2597	-1.1556
C	-0.1602	-2.5670	-2.3444
C	0.2328	-1.2057	-2.0244
C	0.2169	0.0294	-2.7254
C	0.2328	1.2490	-1.9976
C	-0.1602	2.6169	-2.2881
C	-0.2610	3.2838	-1.0846
C	0.0467	2.3355	-0.0266
C	-0.1039	2.3517	1.3803
N	-0.5381	-0.0153	1.4283
N	0.4327	-1.1729	-0.6780
N	0.4327	1.1872	-0.6522
H	0.5941	1.2899	4.1883
H	0.5949	-1.3805	4.1587
H	-0.5896	-4.2835	-1.0306
H	-0.3810	-2.9474	-3.3336
H	-0.3812	3.0183	-3.2688
H	-0.5902	4.3044	-0.9376
H	-0.9466	-0.0053	0.5103
H	-0.1442	-3.3350	1.8427
H	0.0422	0.0411	-3.7952
H	-0.1433	3.2948	1.9148
H	0.6027	0.0037	-0.3479

#P b3lyp/6-31g* empiricaldispersion=gd3bj symm=(veryloose,follow) integral=ultrafinegrid guess=read opt=(ts,calcfc,maxstep=16,tight) freq scrf=solvent=chloroform,read) scf=(save,intrep,xqc,maxconventionalcycles=50)

Charge = 0 Multiplicity = 1

Full point group C1 NOp 1

SCF Done: E(RB3LYP) = -742.494883789 A.U. after 3 cycles

Frequencies -- -1099.7336 92.2744 128.0847

Sum of electronic and thermal Free Energies= -742.308486

---

triphyrin[1.1.1]-16,17-diido-1κ^2*N*^{16,17},2κ*N*^{18}-1λ^2-dihydron‡

triphyrin[1.1.1]-16,17-diido-1κ^2*N*^{16,17},2κ*N*^{18}-1-(*A*-2)-dihydron‡

desmo[*H*^{1};16,*H*^{1};17],18*H*-triphyrin[1.1.1]‡

desmo[*H*^{1};16,*H*^{1};17],18*H*-triphyrin[1.1.1]‡

13

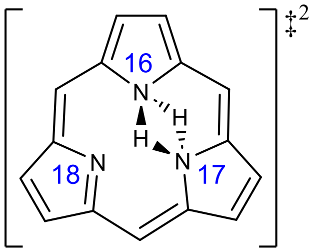

C	-0.1106	-0.0342	-2.3231
C	0.1178	-1.0771	-3.2879
C	0.1833	-2.3004	-2.6282
C	0.0025	-2.0646	-1.2227
C	0.0824	-2.7775	0.0000
C	0.0025	-2.0646	1.2227
C	0.1833	-2.3004	2.6282
C	0.1178	-1.0771	3.2879
C	-0.1106	-0.0342	2.3231
C	-0.0779	1.3745	2.3245
C	-0.1679	2.1103	1.1072
C	0.3413	3.4214	0.6879
C	0.3413	3.4214	-0.6879
C	-0.1679	2.1103	-1.1072
C	-0.0779	1.3745	-2.3245
N	-0.2103	-0.6952	-1.1060
N	-0.2103	-0.6952	1.1060
N	-0.5318	1.4360	0.0000
H	0.2511	-0.9281	-4.3521
H	0.3774	-3.2597	-3.0906
H	0.3774	-3.2597	3.0906
H	0.2511	-0.9281	4.3521
H	0.7090	4.2076	1.3357
H	0.7090	4.2076	-1.3357
H	0.2629	-3.8458	0.0000
H	0.1481	1.8871	3.2546
H	0.1481	1.8871	-3.2546
H	-0.8865	-0.4488	0.0000
H	0.3624	-0.3326	0.0000

#P b3lyp/6-31g* empiricaldispersion=gd3bj symm=(veryloose,follow) integral=ultrafinegrid geom=check guess=read opt=(ts,saddle=2,calcfc,tight) freq scrf=(solvent=chloroform,read) scf=(save,intrep,xqc,maxconventionalcycles=50)

Charge = 0 Multiplicity = 1

Full point group CS NOp 2

SCF Done: E(RB3LYP) = -742.408589457 A.U. after 1 cycles

Frequencies -- -2359.9704 -1272.0489 58.0208

Sum of electronic and thermal Free Energies= -742.229093

---

triphyrin[1.1.1]-16,17-diido-1κ^2*N*^{16,17},2κ^2*N*^{16,17}-1λ^2,2λ^2-dihydron‡^{2}

triphyrin[1.1.1]-16,17-diido-1κ^2*N*^{16,17},2κ^2*N*^{16,17}-1(*A*-2),2(*A*-2)-dihydron‡^{2}

desmo[*H*^{1};16,*H*^{1};17],*desmo*[*H*^{2};16,*H*^{2};17]-triphyrin[1.1.1]‡^{2}

desmo[*H*^{1};16,*H*^{1};17],*desmo*[*H*^{2};16,*H*^{2};17]-triphyrin[1.1.1]‡^{2}

14

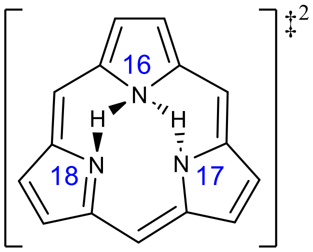

C	-0.1104	-1.1865	2.0618
C	-0.0644	-0.6982	3.3958
C	0.0644	0.6982	3.3958
C	0.1104	1.1865	2.0618
C	0.2701	2.3820	1.3521
C	0.3658	2.3528	-0.0816
C	0.0000	3.3102	-1.1199
C	-0.0722	2.6062	-2.3016
C	0.2264	1.2113	-1.9877
C	0.0000	0.0000	-2.6928
C	-0.2264	-1.2113	-1.9877
C	0.0722	-2.6062	-2.3016
C	0.0000	-3.3102	-1.1199
C	-0.3658	-2.3528	-0.0816
C	-0.2701	-2.3820	1.3521
N	0.0000	0.0000	1.2686
N	0.5914	1.1633	-0.6812
N	-0.5914	-1.1633	-0.6812
H	-0.1383	-1.3129	4.2835
H	0.1383	1.3129	4.2835
H	-0.2350	4.3565	-0.9705
H	-0.3805	2.9892	-3.2663
H	0.3805	-2.9892	-3.2663
H	0.2350	-4.3565	-0.9705
H	0.2481	3.3262	1.8850
H	0.0000	0.0000	-3.7800
H	-0.2481	-3.3262	1.8850
H	0.7225	0.1064	0.4517
H	-0.7225	-0.1064	0.4517

#P b3lyp/6-31g* empiricaldispersion=gd3bj symm=(veryloose,follow) integral=ultrafinegrid geom=check guess=read opt=(ts,saddle=2,calcall,tight) freq scrf=(solvent=chloroform,read) scf=(save,intrep,xqc,maxconventionalcycles=50)

Charge = 0 Multiplicity = 1

Full point group C2 NOp 2

SCF Done: E(RB3LYP) = -742.441167418 A.U. after 5 cycles

Frequencies -- -601.7030 -427.4084 105.2877

Sum of electronic and thermal Free Energies= -742.256540

---

triphyrin[1.1.1]-16,17-diido-$1\kappa^2N^{16,17}$,$2\kappa^2N^{16,18}$-$1\lambda^2$,$2\lambda^2$-dihydron‡^{2}

triphyrin[1.1.1]-16,17-diido-$1\kappa^2N^{16,17}$,$2\kappa^2N^{16,18}$-1(*A*-2),2(*A*-2)-dihydron‡^{2}

desmo[H^1;16,H^1;17],*desmo*[H^2;16,H^2;18]-triphyrin[1.1.1]‡^{2}

desmo[H^1;16,H^1;17],*desmo*[H^2;16,H^2;18]-triphyrin[1.1.1]‡^{2}

15

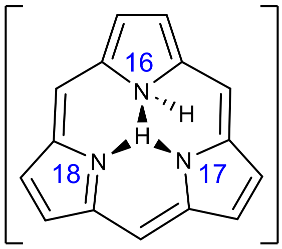

‡^{NC} or ‡^{q2S}

C	-2.0674	0.1590	1.1722
C	-3.2469	-0.4794	0.6965
C	-3.2469	-0.4794	-0.6965
C	-2.0674	0.1590	-1.1722
C	-1.3365	0.0467	-2.3614
C	0.0796	0.1786	-2.3791
C	1.0847	-0.4208	-3.2472
C	2.2653	-0.4247	-2.5384
C	1.9862	0.1706	-1.2368
C	2.6623	0.0390	0.0000
C	1.9862	0.1706	1.2368
C	2.2653	-0.4247	2.5384
C	1.0847	-0.4208	3.2472
C	0.0796	0.1786	2.3791
C	-1.3365	0.0467	2.3614
N	-1.4240	0.6710	0.0000
N	0.7030	0.6186	-1.2629
N	0.7030	0.6186	1.2629
H	-3.9477	-1.0164	1.3224
H	-3.9477	-1.0164	-1.3224
H	0.9102	-0.8472	-4.2271
H	3.2089	-0.8551	-2.8506
H	3.2089	-0.8551	2.8506
H	0.9102	-0.8472	4.2271
H	-1.2804	1.6822	0.0000
H	-1.8362	-0.3826	-3.2238
H	3.6601	-0.3893	0.0000
H	-1.8362	-0.3826	3.2238
H	-0.0788	0.5572	0.0000

#P b3lyp/6-31g* empiricaldispersion=gd3bj symm=(veryloose,follow) integral=ultrafinegrid guess=read opt=(tight,modredundant,maxstep=13) freq scrf=(solvent=chloroform,read) scf=(save,intrep,xqc,maxconventionalcycles=50)

Charge = 0 Multiplicity = 1

Full point group CS NOp 2

SCF Done: E(RB3LYP) = -742.455256939 A.U. after 2 cycles

Frequencies -- -1296.5265 -1282.6869 118.2921

Sum of electronic and thermal Free Energies= -742.272758

---

triphyrin[1.1.1]-16,17-diido-1κ^3*N*^{16,17,18},2κ*N*^{16}-1λ^3-dihydron‡^{NC}

triphyrin[1.1.1]-16,17-diido-1κ^3*N*^{16,17,18},2κ*N*^{16}-1λ^3-dihydron‡^{q2S}

triphyrin[1.1.1]-16,17-diido-1κ^3*N*^{16,17,18},2κ*N*^{16}-1(*TPY*-3)-dihydron‡^{NC}

triphyrin[1.1.1]-16,17-diido-1κ^3*N*^{16,17,18},2κ*N*^{16}-1(*TPY*-3)-dihydron‡^{q2S}

desmo[*H*^{1};16,*H*^{1};17,*H*^{1};18],16*H*-triphyrin[1.1.1]‡^{NC}

desmo[*H*^{1};16,*H*^{1};17,*H*^{1};18],16*H*-triphyrin[1.1.1]‡^{q2S}

desmo[*H*^{1};16,*H*^{1};17,*H*^{1};18],16*H*-triphyrin[1.1.1]‡^{NC}

desmo[*H*^{1};16,*H*^{1};17,*H*^{1};18],16*H*-triphyrin[1.1.1]‡^{q2S}

Structure obtained by constraining the bond lengths to the κ3 hydron. Structure is a non-critical point on the PES - has some of the character of a second-order transition-state structure but is not a true second-order saddle point (is a quasi-second-order transition-state structure).

16

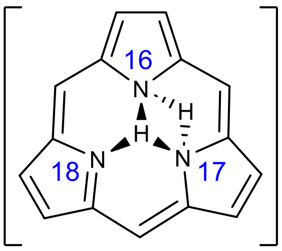

‡^{NC} or ‡^{q3S}

C	-0.1573	-0.0400	2.3131
C	0.1416	-1.0756	3.2666
C	0.2287	-2.2936	2.6121
C	-0.0103	-2.0566	1.2126
C	0.0964	-2.7773	0.0000
C	-0.0103	-2.0566	-1.2126
C	0.2287	-2.2936	-2.6121
C	0.1416	-1.0756	-3.2666
C	-0.1573	-0.0400	-2.3131
C	-0.0583	1.3647	-2.3368
C	-0.0748	2.0956	-1.1135
C	0.2964	3.4778	-0.6899
C	0.2964	3.4778	0.6899
C	-0.0748	2.0956	1.1135
C	-0.0583	1.3647	2.3368
N	-0.3052	-0.7064	1.0901
N	-0.3052	-0.7064	-1.0901
N	-0.3347	1.3576	0.0000
H	0.3380	-0.9125	4.3161
H	0.4922	-3.2482	3.0534
H	0.4922	-3.2482	-3.0534
H	0.3380	-0.9125	-4.3161
H	0.5529	4.3388	-1.3266
H	0.5529	4.3388	1.3266
H	0.3467	-3.8267	0.0000
H	0.1558	1.8559	-3.2821
H	0.1558	1.8559	3.2821
H	-1.0609	-0.6548	0.0000
H	-0.0230	-0.1699	0.0000

#P b3lyp/6-31g* empiricaldispersion=gd3bj symm=(veryloose,follow) integral=ultrafinegrid guess=read freq scrf=(solvent=chloroform,read) scf=(save,intrep,xqc,maxconventionalcycles=50)

Charge = 0 Multiplicity = 1

Full point group CS NOp 2

SCF Done: E(RB3LYP) = -742.394084746 A.U. after 12 cycles

Frequencies -- -2411.0409 -955.4014 -882.4539
Frequencies -- 123.4600 147.9788 151.1270

Sum of electronic and thermal Free Energies= -742.215843

---

triphyrin[1.1.1]-16,17-diido-1$\kappa^3N^{16,17,18}$,2$\kappa^2N^{16,17}$-1λ^3,2λ^2-dihydron‡^{NC}

triphyrin[1.1.1]-16,17-diido-1$\kappa^3N^{16,17,18}$,2$\kappa^2N^{16,17}$-1λ^3,2λ^2-dihydron‡^{q3S}

triphyrin[1.1.1]-16,17-diido-1$\kappa^3N^{16,17,18}$,2$\kappa^2N^{16,17}$-1(*TPY*-3),2(*A*-2)-dihydron‡^{NC}

triphyrin[1.1.1]-16,17-diido-1$\kappa^3N^{16,17,18}$,2$\kappa^2N^{16,17}$-1(*TPY*-3),2(*A*-2)-dihydron‡^{q3S}

desmo[H^1;16,H^1;17,H^1;18],*desmo*[H^2;16,H^2;17]-triphyrin[1.1.1] ‡^{NC}

desmo[H^1;16,H^1;17,H^1;18],*desmo*[H^2;16,H^2;17]-triphyrin[1.1.1] ‡^{q3S}

desmo[H^1;16,H^1;17,H^1;18],*desmo*[H^2;16,H^2;17]-triphyrin[1.1.1] ‡^{NC}

desmo[H^1;16,H^1;17,H^1;18],*desmo*[H^2;16,H^2;17]-triphyrin[1.1.1] ‡^{q3S}

Structure is a non-critical point on the PES - has some of the character of a third-order transition-state structure but is not a true third-order saddle point (is a quasi-third-order transition-state structure).

17

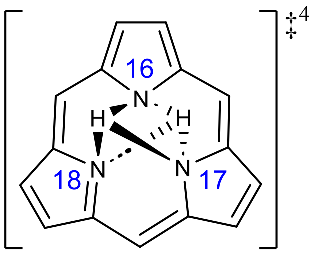

C	-2.3681	0.0511	0.0366
C	-3.3333	1.1241	-0.0862
C	-2.6402	2.3247	-0.0862
C	-1.2284	2.0253	0.0366
C	0.0000	2.7352	0.0051
C	1.2284	2.0253	0.0366
C	2.6402	2.3247	-0.0862
C	3.3333	1.1241	-0.0862
C	2.3681	0.0511	0.0366
C	2.3687	-1.3676	0.0051
C	1.1398	-2.0764	0.0366
C	0.6931	-3.4488	-0.0862
C	-0.6931	-3.4488	-0.0862
C	-1.1398	-2.0764	0.0366
C	-2.3687	-1.3676	0.0051
N	-1.1508	0.6644	0.1314
N	1.1508	0.6644	0.1314
N	0.0000	-1.3288	0.1314
H	-4.4058	1.0092	-0.1821
H	-3.0769	3.3109	-0.1821
H	3.0769	3.3109	-0.1821
H	4.4058	1.0092	-0.1821
H	1.3289	-4.3202	-0.1821
H	-1.3289	-4.3202	-0.1821
H	0.0000	3.8159	-0.0856
H	3.3047	-1.9080	-0.0856
H	-3.3047	-1.9080	-0.0856
H	0.0000	0.0000	0.7595
H	0.0000	0.0000	-0.4748

#P b3lyp/6-31g* empiricaldispersion=gd3bj symm=(veryloose,follow) integral=ultrafinegrid geom=check guess=read freq scrf=(solvent=chloroform,read)scf=(save,intrep,xqc,maxconventionalcycles=50)

Charge = 0 Multiplicity = 1

Full point group C3V NOp 6

SCF Done: E(RB3LYP) = -742.394613555 A.U. after 1 cycles

Frequencies -- -2002.9336 -2000.7258 -1066.7840
Frequencies -- -1063.1053 36.8434 136.4476

Sum of electronic and thermal Free Energies= -742.219738

---

triphyrin[1.1.1]-16,17-diido-1κ^3$N^{16,17,18}$,2κ^3$N^{16,17,18}$-1λ^3,2λ^3-dihydron‡^{4}

triphyrin[1.1.1]-16,17-diido-1κ^3$N^{16,17,18}$,2κ^3$N^{16,17,18}$-1(*TPY*-3),2(*TPY*-3)-dihydron‡^{4}

desmo[H^1;16,H^1;17,H^1;18],*desmo*[H^2;16,H^2;17,H^2;18]-triphyrin[1.1.1] ‡^{4}

desmo[H^1;16,H^1;17,H^1;18],*desmo*[H^2;16,H^2;17,H^2;18]-triphyrin[1.1.1] ‡^{4}

18

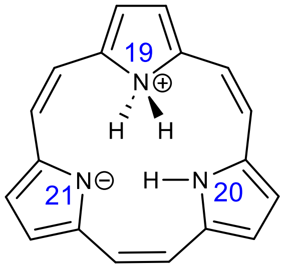

C	0.8300	-2.8909	0.0000
C	2.0731	-3.5010	0.0000
C	3.0960	-2.5425	0.0000
C	2.5842	-1.2533	0.0000
C	3.2885	-0.0454	0.0000
C	2.9848	1.3020	0.0000
C	1.8244	2.1042	0.0000
C	1.8981	3.5234	0.0000
C	0.6141	4.0335	0.0000
C	-0.2826	2.9390	0.0000
C	-1.6879	3.0149	0.0000
C	-2.6551	2.0366	0.0000
C	-2.6590	0.6147	0.0000
C	-3.9347	-0.0556	0.0000
C	-3.6747	-1.3945	0.0000
C	-2.2413	-1.5381	0.0000
C	-1.7136	-2.8496	0.0000
C	-0.4541	-3.4243	0.0000
N	1.0946	-1.4116	0.0000
N	0.4751	1.7856	0.0000
N	-1.6262	-0.2967	0.0000
H	2.2072	-4.5738	0.0000
H	4.1572	-2.7528	0.0000
H	4.3576	-0.2477	0.0000
H	3.8724	1.9289	0.0000
H	2.8288	4.0750	0.0000
H	0.3118	5.0718	0.0000
H	-2.0553	4.0380	0.0000
H	-3.6683	2.4328	0.0000
H	-4.8961	0.4428	0.0000
H	-4.3774	-2.2182	0.0000
H	-2.5091	-3.5909	0.0000
H	-0.4380	-4.5118	0.0000
H	0.6312	-0.9760	0.8087
H	0.6312	-0.9760	-0.8087
H	0.0000	0.8787	0.0000

#P b3lyp/6-31g* empiricaldispersion=gd3bj symm=(veryloose,follow) integral=ultrafinegrid geom=check guess=read opt=tight freq scrf=(solvent=chloroform,read) scf=(save,intrep,xqc,maxconventionalcycles=50)

Charge = 0 Multiplicity = 1

Full point group CS NOp 2

SCF Done: E(RB3LYP) = -859.227088214 A.U. after 7 cycles

Frequencies -- 7.1295 43.9093 102.2640

Sum of electronic and thermal Free Energies= -858.979784

---

triphyrin[2.2.2]-19,20,21-triido-1κN^{19},2κN^{19},3κN^{20}-trihydron

19*H*,19*H*,20*H*-triphyrin[2.2.2]

19

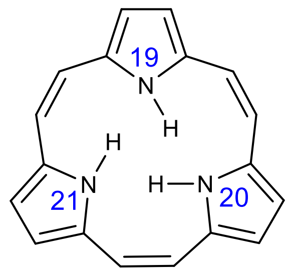

C	2.6819	-0.9227	-0.2555
C	3.9829	-0.3842	-0.1879
C	3.8807	0.9588	0.1624
C	2.5135	1.2822	0.2832
C	2.0145	2.5919	0.5253
C	0.7869	3.1931	0.4208
C	-0.5278	2.7512	0.0945
C	-1.5398	3.5828	-0.4053
C	-2.6867	2.8127	-0.6106
C	-2.4096	1.4947	-0.2238
C	-3.3589	0.4384	-0.1568
C	-3.2612	-0.9067	0.0861
C	-2.1697	-1.8134	0.1830
C	-2.2673	-3.1531	0.5793
C	-1.0186	-3.7560	0.4133
C	-0.1213	-2.7964	-0.0743
C	1.2471	-3.0575	-0.3731
C	2.3805	-2.2939	-0.4794
N	1.8160	0.1169	0.0265
N	-1.0643	1.4596	0.1611
N	-0.8314	-1.5908	-0.1700
H	4.8865	-0.9535	-0.3593
H	4.6866	1.6685	0.2929
H	2.8251	3.2783	0.7573
H	0.8144	4.2738	0.5412
H	-1.4080	4.6329	-0.6284
H	-3.6361	3.1451	-1.0089
H	-4.3736	0.8033	-0.2956
H	-4.2142	-1.4172	0.2038
H	-3.1706	-3.6127	0.9571
H	-0.7476	-4.7755	0.6529
H	1.4241	-4.1256	-0.4752
H	3.2804	-2.8617	-0.7027
H	0.8159	0.0232	0.0735
H	-0.7708	0.8038	0.8742
H	-0.6167	-0.9138	-0.8923

#P b3lyp/6-31g* empiricaldispersion=gd3bj symm=(veryloose,follow) integral=ultrafinegrid geom=check guess=read opt=(maxstep=3,tight) freq scrf=(solvent=chloroform,read) scf=(save,intrep,xqc,maxconventionalcycles=50)

Charge = 0 Multiplicity = 1

Full point group C1 NOp 1

SCF Done: E(RB3LYP) = -859.277311826 A.U. after 6 cycles

Frequencies -- 36.2215 54.6810 79.9726

Sum of electronic and thermal Free Energies= -859.028607

---

triphyrin[2.2.2]-19,20,21-triido-1κN^{19},2κN^{20},3κN^{21}-trihydron

19*H*,20*H*,21*H*-triphyrin[2.2.2]

20

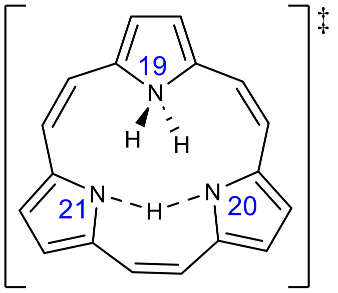

C	0.0000	1.1936	2.7607
C	0.0000	0.7022	4.0545
C	0.0000	-0.7022	4.0545
C	0.0000	-1.1936	2.7607
C	0.0000	-2.4905	2.2642
C	0.0000	-3.0082	0.9844
C	0.0000	-2.5583	-0.3603
C	0.0000	-3.5900	-1.3548
C	0.0000	-2.9946	-2.5857
C	0.0000	-1.5909	-2.3610
C	0.0000	-0.6844	-3.4402
C	0.0000	0.6844	-3.4402
C	0.0000	1.5909	-2.3610
C	0.0000	2.9946	-2.5857
C	0.0000	3.5900	-1.3548
C	0.0000	2.5583	-0.3603
C	0.0000	3.0082	0.9844
C	0.0000	2.4905	2.2642
N	0.0000	0.0000	1.8598
N	0.0000	-1.3146	-1.0022
N	0.0000	1.3146	-1.0022
H	0.0000	1.3383	4.9291
H	0.0000	-1.3383	4.9291
H	0.0000	-3.2307	3.0605
H	0.0000	-4.0946	0.9982
H	0.0000	-4.6481	-1.1258
H	0.0000	-3.4608	-3.5621
H	0.0000	-1.1694	-4.4128
H	0.0000	1.1694	-4.4128
H	0.0000	3.4608	-3.5621
H	0.0000	4.6481	-1.1258
H	0.0000	4.0946	0.9982
H	0.0000	3.2307	3.0605
H	0.8172	0.0000	1.2371
H	-0.8172	0.0000	1.2371
H	0.0000	0.0000	-0.7793

#P b3lyp/6-31g* empiricaldispersion=gd3bj symm=(veryloose,follow) integral=ultrafinegrid guess=read opt=(ts,readfc,recalcfc=3,tight) freq scrf=(solvent=chloroform,read) scf=(save,intrep,xqc,maxconventionalcycles=50)

Charge = 0 Multiplicity = 1

Full point group C2V NOp 4

SCF Done: E(RB3LYP) = -859.207206234 A.U. after 1 cycles

Frequencies -- -1766.8541 30.2806 41.8663

Sum of electronic and thermal Free Energies= -858.961853

---

triphyrin[2.2.2]-19,20,21-triido-1κ*N*[19],2κ*N*[19],3κ[2]*N*[20,21]-3λ[2]-trihydron‡

triphyrin[2.2.2]-19,20,21-triido-1κ*N*[19],2κ*N*[19],3κ[2]*N*[20,21]-3(*A*-2)-trihydron‡

19*H*,19*H*-*desmo*[*H*[3];20,*H*[3];21]-triphyrin[2.2.2]

21

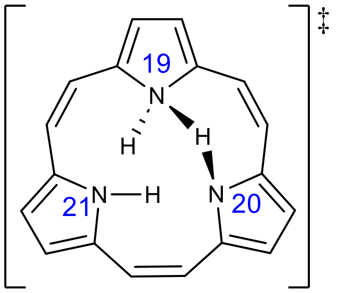

C	0.1957	-2.5338	-0.3278
C	1.0993	-3.6364	-0.1703
C	2.2983	-3.1307	0.2587
C	2.1520	-1.7129	0.2945
C	3.2739	-0.8883	0.5407
C	3.4617	0.4663	0.3929
C	2.5340	1.4647	0.0723
C	2.7510	2.7234	-0.4495
C	1.5257	3.3724	-0.6481
C	0.4615	2.5772	-0.2488
C	-0.8544	3.0689	-0.0925
C	-2.0984	2.5629	0.2089
C	-2.5984	1.2493	0.2676
C	-3.9325	0.8621	0.5314
C	-4.0416	-0.5048	0.3045
C	-2.7704	-0.9830	-0.0824
C	-2.4027	-2.2937	-0.4462
C	-1.1675	-2.8771	-0.5894
N	0.8619	-1.3475	-0.0605
N	1.0629	1.2934	0.2400
N	-1.9155	0.0957	-0.0627
H	0.8244	-4.6742	-0.3098
H	3.2101	-3.6658	0.4914
H	4.1659	-1.4532	0.7989
H	4.4827	0.8323	0.4612
H	3.7305	3.1117	-0.6939
H	1.3954	4.3733	-1.0381
H	-0.8542	4.1459	-0.2469
H	-2.8701	3.3139	0.3550
H	-4.7131	1.5418	0.8456
H	-4.9214	-1.1238	0.4142
H	-3.2522	-2.9547	-0.5972
H	-1.2346	-3.9273	-0.8647
H	0.7885	0.1959	-0.0880
H	0.8636	1.2215	1.2444
H	-1.0046	0.0526	-0.4898

#P b3lyp/6-31g* empiricaldispersion=gd3bj symm=(veryloose,follow) integral=ultrafinegrid guess=read opt=(ts,readfc,recalc=3,tight) freq scrf=(solvent=chloroform,read) scf=(save,intrep,xqc,maxconventionalcycles=50)

Charge = 0 Multiplicity = 1

Full point group C1 NOp 1

SCF Done: E(RB3LYP) = -859.223316973 A.U. after 5 cycles

Frequencies -- -670.5419 36.1037 48.1890

Sum of electronic and thermal Free Energies= -858.978412

---

triphyrin[2.2.2]-19,20,21-triido-1κN^{19},2κN^{21},3κ$^2N^{19,20}$-3λ^2-trihydron‡

triphyrin[2.2.2]-19,20,21-triido-1κN^{19},2κN^{21},3κ$^2N^{19,20}$-3(*A*-2)-trihydron‡

19*H*,21*H*,*desmo*[*H*^{3};19,*H*^{3};20]-triphyrin[2.2.2]

22

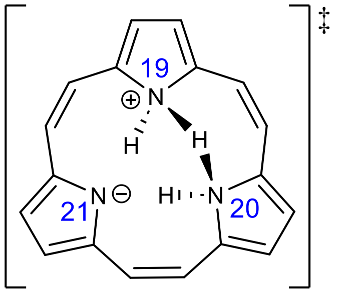

C	0.0000	2.5568	-0.2553
C	-0.6642	3.4232	-1.1176
C	-0.8893	2.8067	-2.3481
C	-0.3395	1.5322	-2.3348
C	-0.2146	0.6539	-3.4191
C	0.2146	-0.6539	-3.4191
C	0.3395	-1.5322	-2.3348
C	0.8893	-2.8067	-2.3481
C	0.6642	-3.4232	-1.1176
C	0.0000	-2.5568	-0.2553
C	-0.4885	-2.9567	1.0042
C	-0.7379	-2.3104	2.1960
C	-0.4160	-0.9977	2.5852
C	-0.2874	-0.6203	3.9784
C	0.2874	0.6203	3.9784
C	0.4160	0.9977	2.5852
C	0.7379	2.3104	2.1960
C	0.4885	2.9567	1.0042
N	0.2219	1.2889	-0.9973
N	-0.2219	-1.2889	-0.9973
N	0.0000	0.0000	1.7578
H	-0.9705	4.4213	-0.8338
H	-1.4022	3.2325	-3.2005
H	-0.4478	1.1029	-4.3809
H	0.4478	-1.1029	-4.3809
H	1.4022	-3.2325	-3.2005
H	0.9705	-4.4213	-0.8338
H	-0.5619	-4.0417	1.0602
H	-1.0790	-2.9526	3.0051
H	-0.5508	-1.2416	4.8258
H	0.5508	1.2416	4.8258
H	1.0790	2.9526	3.0051
H	0.5619	4.0417	1.0602
H	-1.2359	-1.1583	-1.0749
H	1.2359	1.1583	-1.0749
H	0.0000	0.0000	-0.6130

#P b3lyp/6-31g* empiricaldispersion=gd3bj symm=(veryloose,follow) integral=ultrafinegrid guess=read opt=(ts,readfc,recalc=4,tight) freq scrf=(solvent=chloroform,read) scf=(save,intrep,xqc,maxconventionalcycles=50)

Charge = 0 Multiplicity = 1

Full point group C2 NOp 2

SCF Done: E(RB3LYP) = -859.179797614 A.U. after 6 cycles

Frequencies -- -1628.2406 34.0837 49.4341

Sum of electronic and thermal Free Energies= -858.936456

---

triphyrin[2.2.2]-19,20,21-triido-1κN^{19},2κN^{20},3κ$^{2}N^{19,20}$-3λ^{2}-trihydron‡

triphyrin[2.2.2]-19,20,21-triido-1κN^{19},2κN^{20},3κ$^{2}N^{19,20}$-3(*A*-2)-trihydron‡

19*H*,20*H*,*desmo*[H^{3};20,H^{3};21]-triphyrin[2.2.2]

23

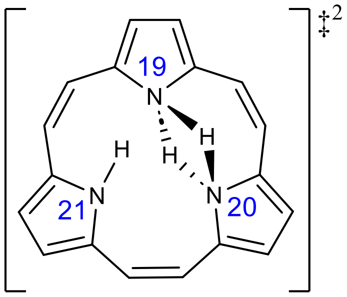

C	0.0000	2.4737	-0.3391
C	0.0000	3.4919	-1.3090
C	0.0000	2.9438	-2.5789
C	0.0000	1.5605	-2.4399
C	0.0000	0.6841	-3.5329
C	0.0000	-0.6841	-3.5329
C	0.0000	-1.5605	-2.4399
C	0.0000	-2.9438	-2.5789
C	0.0000	-3.4919	-1.3090
C	0.0000	-2.4737	-0.3391
C	0.0000	-2.9212	1.0201
C	0.0000	-2.4419	2.3039
C	0.0000	-1.1299	2.8083
C	0.0000	-0.6967	4.1506
C	0.0000	0.6967	4.1506
C	0.0000	1.1299	2.8083
C	0.0000	2.4419	2.3039
C	0.0000	2.9212	1.0201
N	0.0000	1.2083	-1.0332
N	0.0000	-1.2083	-1.0332
N	0.0000	0.0000	2.0295
H	0.0000	4.5456	-1.0631
H	0.0000	3.4645	-3.5267
H	0.0000	1.1854	-4.4961
H	0.0000	-1.1854	-4.4961
H	0.0000	-3.4645	-3.5267
H	0.0000	-4.5456	-1.0631
H	0.0000	-4.0079	1.0067
H	0.0000	-3.2043	3.0780
H	0.0000	-1.3577	5.0065
H	0.0000	1.3577	5.0065
H	0.0000	3.2043	3.0780
H	0.0000	4.0079	1.0067
H	-0.6526	0.0000	-0.8875
H	0.6526	0.0000	-0.8875
H	0.0000	0.0000	1.0260

#P b3lyp/6-31g* empiricaldispersion=gd3bj symm=(veryloose,follow) integral=ultrafinegrid guess=read opt=(ts,saddle=2,readfc,tight) freq scrf=(solvent=chloroform,read) scf=(save,intrep,xqc,maxconventionalcycles=50)

Charge = 0 Multiplicity = 1

Full point group C2V NOp 4

SCF Done: E(RB3LYP) = -859.156607788 A.U. after 1 cycles

Frequencies -- -2369.3588 -1503.6222 39.4783

Sum of electronic and thermal Free Energies= -858.917186

---

triphyrin[2.2.2]-19,20,21-triido-1κ$^2N^{19,20}$,2κ$^2N^{19,20}$,3κN^{21}-1λ^2,2λ^2-trihydron‡^{2}

triphyrin[2.2.2]-19,20,21-triido-1κ$^2N^{19,20}$,2κ$^2N^{19,20}$,3κN^{21}-1(*A*-2),2(*A*-2)-trihydron‡^{2}

desmo[H^1;19,H^1;20],*desmo*[H^2;19,H^2;20],21*H*-triphyrin[2.2.2]

24

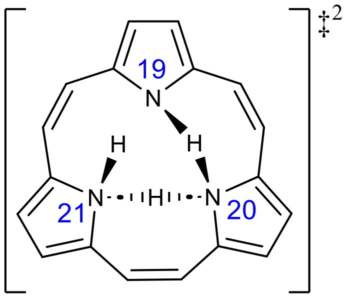

C	1.9383	-1.7093	-0.5787
C	3.3589	-1.8149	-0.7341
C	3.8993	-0.6115	-0.3325
C	2.8008	0.2240	0.0200
C	2.9075	1.4801	0.6620
C	1.9110	2.3581	0.9977
C	0.5422	2.4058	0.6163
C	-0.0805	3.6328	0.3869
C	-1.1978	3.4301	-0.4180
C	-1.3685	2.0615	-0.6247
C	-2.6431	1.5652	-1.0111
C	-3.3180	0.4229	-0.6552
C	-2.7862	-0.7022	-0.0079
C	-3.3672	-1.7420	0.6898
C	-2.3756	-2.6916	1.0081
C	-1.1502	-2.2866	0.4916
C	-0.0581	-3.1226	0.1762
C	1.1585	-2.8849	-0.4220
N	1.6080	-0.4297	-0.1896
N	-0.2673	1.3695	0.0223
N	-1.3448	-0.9476	-0.0425
H	3.8863	-2.7211	-1.0058
H	4.9445	-0.3395	-0.2560
H	3.9201	1.7749	0.9259
H	2.2274	3.2811	1.4773
H	0.3308	4.5838	0.6986
H	-1.8998	4.1819	-0.7547
H	-3.2403	2.3309	-1.4987
H	-4.3866	0.3933	-0.8456
H	-4.4203	-1.8064	0.9284
H	-2.5384	-3.6439	1.4956
H	-0.2758	-4.1667	0.3875
H	1.7107	-3.7866	-0.6786
H	0.4990	0.5960	-0.3940
H	-0.7697	0.1961	0.4033
H	-0.9870	-0.9084	-1.0007

#P b3lyp/6-31g* empiricaldispersion=gd3bj symm=(veryloose,follow) integral=ultrafinegrid guess=read opt=(ts,saddle=2,readfc,tight) freq scrf=(solvent=chloroform,read) scf=(save,intrep,xqc,maxconventionalcycles=50)

Charge = 0 Multiplicity = 1

Full point group C1 NOp 1

SCF Done: E(RB3LYP) = -859.173236111 A.U. after 6 cycles

Frequencies -- -1745.5432 -829.6877 53.2237

Sum of electronic and thermal Free Energies= -858.932066

---

triphyrin[2.2.2]-19,20,21-triido-1κ$^{2}N^{19,20}$,2κ$^{2}N^{20,21}$,3κN^{21}-1λ^{2},2λ^{2}-trihydron‡^{2}

triphyrin[2.2.2]-19,20,21-triido-1κ$^{2}N^{19,20}$,2κ$^{2}N^{20,21}$,3κN^{21}-1(*A*-2),2(*A*-2)-trihydron‡^{2}

desmo[H^{1};19,H^{1};20],*desmo*[H^{2};20,H^{2};21],21*H*-triphyrin[2.2.2]

25

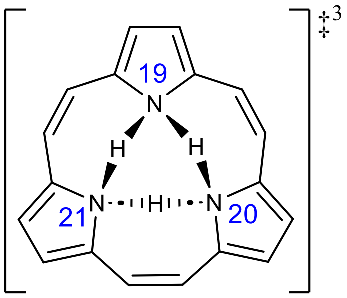

C	-2.1659	1.2886	-0.6425
C	-2.4430	2.5802	-1.1055
C	-1.2341	3.1914	-1.4368
C	-0.1882	2.3130	-1.1479
C	1.1709	2.5802	-1.4210
C	2.3047	1.9763	-0.9535
C	2.4183	1.0067	0.0690
C	3.5375	0.9178	0.9098
C	3.1825	0.1399	2.0079
C	1.8775	-0.3281	1.7962
C	1.3186	-1.3972	2.5329
C	0.4044	-2.3231	2.1140
C	-0.3460	-2.3934	0.9203
C	-1.0019	-3.5420	0.4739
C	-1.7017	-3.2387	-0.6936
C	-1.5235	-1.8774	-0.9671
C	-2.6371	-1.0885	-1.3989
C	-2.9876	0.1932	-1.0592
N	-0.7508	1.1530	-0.5047
N	1.3701	0.2161	0.5941
N	-0.5462	-1.3599	-0.0633
H	-3.4346	2.9397	-1.3489
H	-1.0956	4.1658	-1.8864
H	1.3064	3.4444	-2.0657
H	3.2530	2.3995	-1.2732
H	4.4682	1.4467	0.7501
H	3.8082	-0.1451	2.8439
H	1.8119	-1.5897	3.4813
H	0.2515	-3.1859	2.7568
H	-0.9840	-4.4879	0.9989
H	-2.4244	-3.8708	-1.1937
H	-3.4012	-1.6718	-1.9055
H	-3.9959	0.4972	-1.3264
H	0.1069	0.9168	0.3838
H	0.5863	-0.8092	-0.0860
H	-0.6810	-0.1086	-0.3014

#P b3lyp/6-31g* empiricaldispersion=gd3bj symm=(veryloose,follow) integral=ultrafinegrid guess=read opt=(ts,saddle=3,readfc,tight) freq scrf=(solvent=chloroform,read) scf=(save,intrep,xqc,maxconventionalcycles=50)

Charge = 0 Multiplicity = 1

Full point group C1 NOp 1

SCF Done: E(RB3LYP) = -859.108633679 A.U. after 6 cycles

Frequencies -- -2095.3437 -1436.2492 -1409.8897
Frequencies -- 55.8341 103.6881 115.9796

Sum of electronic and thermal Free Energies= -858.872855

---

triphyrin[2.2.2]-19,20,21-triido-$1\kappa^2N^{19,20}$,$2\kappa^2N^{20,21}$,$3\kappa^2N^{19,21}$-$1\lambda^2$,$2\lambda^2$,$3\lambda^2$-trihydron$‡^3$

triphyrin[2.2.2]-19,20,21-triido-$1\kappa^2N^{19,20}$,$2\kappa^2N^{20,21}$,$3\kappa^2N^{19,21}$-1(*A*-2),2(*A*-2),3(*A*-2)-trihydron$‡^3$

desmo[H^1;19,H^1;20],*desmo*[H^2;20,H^2;21],*desmo*[H^2;19,H^2;21]-triphyrin[2.2.2]

26

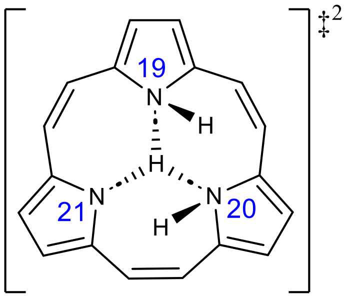

C	0.4945	-0.2835	2.6037
C	0.8009	-1.3880	3.3869
C	0.2623	-2.5394	2.7958
C	-0.3345	-2.1978	1.5956
C	-0.8928	-3.1183	0.6847
C	-0.8928	-3.1183	-0.6847
C	-0.3345	-2.1978	-1.5956
C	0.2623	-2.5394	-2.7958
C	0.8009	-1.3880	-3.3869
C	0.4945	-0.2835	-2.6037
C	0.5573	1.0506	-3.0475
C	0.1366	2.2044	-2.4416
C	-0.1989	2.4739	-1.0940
C	-0.5650	3.7971	-0.6860
C	-0.5650	3.7971	0.6860
C	-0.1989	2.4739	1.0940
C	0.1366	2.2044	2.4416
C	0.5573	1.0506	3.0475
N	-0.1729	-0.7580	1.4020
N	-0.1729	-0.7580	-1.4020
N	-0.0220	1.6537	0.0000
H	1.3222	-1.3306	4.3332
H	0.3332	-3.5526	3.1692
H	-1.1954	-4.0501	1.1559
H	-1.1954	-4.0501	-1.1559
H	0.3332	-3.5526	-3.1692
H	1.3222	-1.3306	-4.3332
H	0.9054	1.1356	-4.0737
H	0.1164	3.0871	-3.0766
H	-0.7539	4.6235	-1.3602
H	-0.7539	4.6235	1.3602
H	0.1164	3.0871	3.0766
H	0.9054	1.1356	4.0737
H	0.1584	-0.1840	0.0000
H	-1.0827	-0.2965	1.3202
H	-1.0827	-0.2965	-1.3202

#P b3lyp/6-31g* empiricaldispersion=gd3bj symm=(veryloose,follow) integral=ultrafinegrid guess=read opt=(ts,saddle=2,readfc,tight) freq scrf=(solvent=chloroform,read) scf=(save,intrep,xqc,maxconventionalcycles=50)

Charge = 0 Multiplicity = 1

Full point group CS NOp 2

SCF Done: E(RB3LYP) = -859.171302622 A.U. after 1 cycles

Frequencies -- -1475.6879 -1058.7935 28.7944

Sum of electronic and thermal Free Energies= -858.930529

---

triphyrin[2.2.2]-19,20,21-triido-1κ^3*N*^{19,20,21},2κ*N*^{19},3κ*N*^{20}-1λ^3-trihydron‡^{2}

triphyrin[2.2.2]-19,20,21-triido-1κ^3*N*^{19,20,21},2κ*N*^{19},3κ*N*^{20}-1(*TPY*-3)-trihydron‡^{2}

desmo[*H*^{1};19,*H*^{1};20,*H*^{1};21],19*H*,20*H*-triphyrin[2.2.2]

27

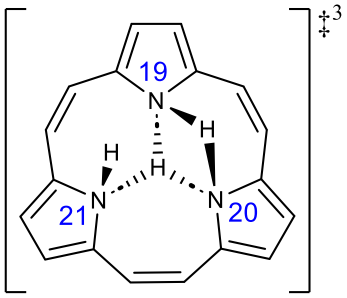

C	-0.6592	-0.3188	2.4397
C	-0.6565	-1.3363	3.4082
C	0.0138	-2.4360	2.8841
C	0.3537	-2.1357	1.5619
C	0.9403	-3.0756	0.6816
C	0.9403	-3.0756	-0.6816
C	0.3537	-2.1357	-1.5619
C	0.0138	-2.4360	-2.8841
C	-0.6565	-1.3363	-3.4082
C	-0.6592	-0.3188	-2.4397
C	-0.8086	1.0279	-2.8833
C	-0.2725	2.1978	-2.4216
C	0.2735	2.4652	-1.1476
C	0.9964	3.5626	-0.7054
C	0.9964	3.5626	0.7054
C	0.2735	2.4652	1.1476
C	-0.2725	2.1978	2.4216
C	-0.8086	1.0279	2.8833
N	-0.0958	-0.8257	1.2423
N	-0.0958	-0.8257	-1.2423
N	-0.0772	1.6889	0.0000
H	-1.0116	-1.2099	4.4231
H	0.2217	-3.3788	3.3730
H	1.3447	-3.9519	1.1808
H	1.3447	-3.9519	-1.1808
H	0.2217	-3.3788	-3.3730
H	-1.0116	-1.2099	-4.4231
H	-1.2515	1.0787	-3.8748
H	-0.2791	3.0462	-3.1002
H	1.4424	4.3065	-1.3519
H	1.4424	4.3065	1.3519
H	-0.2791	3.0462	3.1002
H	-1.2515	1.0787	3.8748
H	0.3911	0.0484	0.0000
H	-0.5618	-0.8970	0.0000
H	-1.0519	1.3921	0.0000

#P b3lyp/6-31g* empiricaldispersion=gd3bj symm=(veryloose,follow) integral=ultrafinegrid guess=read opt=(ts,saddle=3,readfc,tight) freq scrf=(solvent=chloroform,read) scf=(save,intrep,xqc,maxconventionalcycles=50)

Charge = 0 Multiplicity = 1

Full point group CS NOp 2

SCF Done: E(RB3LYP) = -859.136853775 A.U. after 1 cycles

Frequencies -- -2206.2074 -1201.6642 -1195.7486
Frequencies -- 51.4402 75.6983 106.4342

Sum of electronic and thermal Free Energies= -858.899416

---

triphyrin[2.2.2]-19,20,21-triido-1κ^3*N*^{19,20,21},2κ^2*N*^{19,20},3κ*N*^{21}-1λ^3,2λ^2-trihydron‡^{3}

triphyrin[2.2.2]-19,20,21-triido-1κ^3*N*^{19,20,21},2κ^2*N*^{19,20},3κ*N*^{21}-1(*TPY*-3),2(*A*-2)-trihydron‡^{3}

desmo[*H*^{1};19,*H*^{1};20,*H*^{1};21],*desmo*[*H*^{2};19,*H*^{2};20],20*H*-triphyrin[2.2.2]
