# Supplementary material for: The Polytope Formalism: application to molecular constitution and the prospect of a complete description of Chemical Space
Source: Chem Sci. 2026 Jan 8;17(4):2102–18. doi: 10.1039/d5sc08813e (PMC12780917; doi:10.1039/d5sc08813e)
Supplement: SC-017-D5SC08813E-s001 [file S2B5 motions order table.pdf › publication files/motions order outputs/S2B5 motions order table.pdf]

	0:0:0:0:0	1:1:1:1:1	0:0:0:0:1	0:0:0:1:0	0:0:1:0:0	0:1:0:0:0	1:0:0:0:0	1:1:1:1:0	1:1:1:0:1	1:1:0:1:1	1:0:1:1:1	0:1:1:1:1	0:0:0:1:1	0:0:1:0:1	0:0:1:1:0	0:1:0:0:1	0:1:0:1:0	0:1:1:0:0	1:0:0:0:1	1:0:0:1:0	1:0:1:0:0	1:1:0:0:0	1:1:1:0:0	1:1:0:1:0	1:1:0:0:1	1:0:1:1:0	1:0:1:0:1	1:0:0:1:1	0:1:1:1:0	[illegible]
0:0:0:0:0	0	–	–	–	–	–	–	–	–	–	–	–	–	–	–	–	–	–	–	–	–	–	–	–	–	–	–	–	–	[illegible]
1:1:1:1:1	–	0	–	–	–	–	–	–	–	–	–	–	–	–	–	–	–	–	–	–	–	–	–	–	–	–	–	–	–	[illegible]
0:0:0:0:1	–	–	0	–	–	–	–	–	–	–	–	–	–	–	–	–	–	–	–	–	–	–	–	–	–	–	–	–	–	[illegible]
0:0:0:1:0	–	–	–	0	–	–	–	–	–	–	–	–	–	–	–	–	–	–	–	–	–	–	–	–	–	–	–	–	–	[illegible]
0:0:1:0:0	–	–	–	–	0	–	–	–	–	–	–	–	–	–	–	–	–	–	–	–	–	–	–	–	–	–	–	–	–	[illegible]
0:1:0:0:0	–	–	–	–	–	0	–	–	–	–	–	–	–	–	–	–	–	–	–	–	–	–	–	–	–	–	–	–	–	[illegible]
1:0:0:0:0	–	–	–	–	–	–	0	–	–	–	–	–	–	–	–	–	–	–	–	–	–	–	–	–	–	–	–	–	–	[illegible]
1:1:1:1:0	–	–	–	–	–	–	–	0	–	–	–	–	–	–	–	–	–	–	–	–	–	–	–	–	–	–	–	–	–	[illegible]
1:1:1:0:1	–	–	–	–	–	–	–	–	0	–	–	–	–	–	–	–	–	–	–	–	–	–	–	–	–	–	–	–	–	[illegible]
1:1:0:1:1	–	–	–	–	–	–	–	–	–	0	–	–	–	–	–	–	–	–	–	–	–	–	–	–	–	–	–	–	–	[illegible]
1:0:1:1:1	–	–	–	–	–	–	–	–	–	–	0	–	–	–	–	–	–	–	–	–	–	–	–	–	–	–	–	–	–	[illegible]
0:1:1:1:1	–	–	–	–	–	–	–	–	–	–	–	0	–	–	–	–	–	–	–	–	–	–	–	–	–	–	–	–	–	[illegible]
0:0:0:1:1	–	–	–	–	–	–	–	–	–	–	–	–	0	–	–	–	–	–	–	–	–	–	–	–	–	–	–	–	–	[illegible]
0:0:1:0:1	–	–	–	–	–	–	–	–	–	–	–	–	–	0	–	–	–	–	–	–	–	–	–	–	–	–	–	–	–	[illegible]
0:0:1:1:0	–	–	–	–	–	–	–	–	–	–	–	–	–	–	0	–	–	–	–	–	–	–	–	–	–	–	–	–	–	[illegible]
0:1:0:0:1	–	–	–	–	–	–	–	–	–	–	–	–	–	–	–	0	–	–	–	–	–	–	–	–	–	–	–	–	–	[illegible]
0:1:0:1:0	–	–	–	–	–	–	–	–	–	–	–	–	–	–	–	–	0	–	–	–	–	–	–	–	–	–	–	–	–	[illegible]
0:1:1:0:0	–	–	–	–	–	–	–	–	–	–	–	–	–	–	–	–	–	0	–	–	–	–	–	–	–	–	–	–	–	[illegible]
1:0:0:0:1	–	–	–	–	–	–	–	–	–	–	–	–	–	–	–	–	–	–	0	–	–	–	–	–	–	–	–	–	–	[illegible]
1:0:0:1:0	–	–	–	–	–	–	–	–	–	–	–	–	–	–	–	–	–	–	–	0	–	–	–	–	–	–	–	–	–	[illegible]
1:0:1:0:0	–	–	–	–	–	–	–	–	–	–	–	–	–	–	–	–	–	–	–	–	0	–	–	–	–	–	–	–	–	[illegible]
1:1:0:0:0	–	–	–	–	–	–	–	–	–	–	–	–	–	–	–	–	–	–	–	–	–	0	–	–	–	–	–	–	–	[illegible]
1:1:1:0:0	–	–	–	–	–	–	–	–	–	–	–	–	–	–	–	–	–	–	–	–	–	–	0	–	–	–	–	–	–	[illegible]
1:1:0:1:0	–	–	–	–	–	–	–	–	–	–	–	–	–	–	–	–	–	–	–	–	–	–	–	0	–	–	–	–	–	[illegible]
1:1:0:0:1	–	–	–	–	–	–	–	–	–	–	–	–	–	–	–	–	–	–	–	–	–	–	–	–	0	–	–	–	–	[illegible]
1:0:1:1:0	–	–	–	–	–	–	–	–	–	–	–	–	–	–	–	–	–	–	–	–	–	–	–	–	–	0	–	–	–	[illegible]
1:0:1:0:1	–	–	–	–	–	–	–	–	–	–	–	–	–	–	–	–	–	–	–	–	–	–	–	–	–	–	0	–	–	[illegible]
1:0:0:1:1	–	–	–	–	–	–	–	–	–	–	–	–	–	–	–	–	–	–	–	–	–	–	–	–	–	–	–	0	–	[illegible]
0:1:1:1:0	–	–	–	–	–	–	–	–	–	–	–	–	–	–	–	–	–	–	–	–	–	–	–	–	–	–	–	–	0	[illegible]
0:1:1:0:1	–	–	–	–	–	–	–	–	–	–	–	–	–	–	–	–	–	–	–	–	–	–	–	–	–	–	–	–	–	[illegible]
0:1:0:1:1	–	–	–	–	–	–	–	–	–	–	–	–	–	–	–	–	–	–	–	–	–	–	–	–	–	–	–	–	–	[illegible]
0:0:1:1:1	–	–	–	–	–	–	–	–	–	–	–	–	–	–	–	–	–	–	–	–	–	–	–	–	–	–	–	–	–	[illegible]
0:0:0:0:0,1,2	2	–	2	–	–	–	–	–	–	–	–	–	–	–	–	–	–	–	–	–	–	–	–	–	–	–	–	–	–	[illegible]
0:0:0:0,1,2:0	2	–	–	2	–	–	–	–	–	–	–	–	–	–	–	–	–	–	–	–	–	–	–	–	–	–	–	–	–	[illegible]
0:0:0,1,2:0:0	2	–	–	–	2	–	–	–	–	–	–	–	–	–	–	–	–	–	–	–	–	–	–	–	–	–	–	–	–	[illegible]
0:0,1,2:0:0:0	2	–	–	–	–	2	–	–	–	–	–	–	–	–	–	–	–	–	–	–	–	–	–	–	–	–	–	–	–	[illegible]
[illegible]	[illegible]	[illegible]	[illegible]	[illegible]	[illegible]	[illegible]	[illegible]	[illegible]	[illegible]	[illegible]	[illegible]	[illegible]	[illegible]	[illegible]	[illegible]	[illegible]	[illegible]	[illegible]	[illegible]	[illegible]	[illegible]	[illegible]	[illegible]	[illegible]	[illegible]	[illegible]	[illegible]	[illegible]	[illegible]	[illegible]
